# Tandem gene duplications drive divergent evolution of caffeine and crocin biosynthetic pathways in plants

**DOI:** 10.1186/s12915-020-00795-3

**Published:** 2020-06-18

**Authors:** Zhichao Xu, Xiangdong Pu, Ranran Gao, Olivia Costantina Demurtas, Steven J. Fleck, Michaela Richter, Chunnian He, Aijia Ji, Wei Sun, Jianqiang Kong, Kaizhi Hu, Fengming Ren, Jiejie Song, Zhe Wang, Ting Gao, Chao Xiong, Haoying Yu, Tianyi Xin, Victor A. Albert, Giovanni Giuliano, Shilin Chen, Jingyuan Song

**Affiliations:** 1 Key Lab of Chinese Medicine Resources Conservation, State Administration of Traditional Chinese Medicine of the People’s Republic of China, Institute of Medicinal Plant Development, Chinese Academy of Medical Sciences & Peking Union Medical College, Beijing, 100193 China; 2 Engineering Research Center of Chinese Medicine Resource, Ministry of Education, Beijing, 100193 China; 3 Italian National Agency for New Technologies, Energy and Sustainable Economic Development (ENEA), Casaccia Res. Ctr, 00123 Rome, Italy; 4 Department of Biological Sciences, University at Buffalo, Buffalo, NY 14260 USA; 5 Institute of Chinese Materia Medica, China Academy of Chinese Medical Sciences, Beijing, 100700 China; 6 Institute of Materia Medica, Chinese Academy of Medical Sciences & Peking Union Medical College, Beijing, 100050 China; 7 Chongqing Institute of Medicinal Plant Cultivation, Chongqing, 408435 China; 8 College of Life Sciences, Qingdao Agricultural University, Qingdao, 266109 China; 9 School of Biological Sciences, Nanyang Technological University, Singapore, 637551 Singapore; 10 Yunnan Branch, Institute of Medicinal Plant Development, Chinese Academy of Medical Sciences & Peking Union Medical College, Jinghong, 666100 China

**Keywords:** Crocin biosynthesis, Caffeine biosynthesis, *Gardenia jasminoides*, *Coffea canephora*, Genomics, Carotenoid cleavage dioxygenases, Aldehyde dehydrogenases, UDP-glucosyltransferases, *N*-Methyltransferases

## Abstract

**Background:**

Plants have evolved a panoply of specialized metabolites that increase their environmental fitness. Two examples are caffeine, a purine psychotropic alkaloid, and crocins, a group of glycosylated apocarotenoid pigments. Both classes of compounds are found in a handful of distantly related plant genera (*Coffea*, *Camellia*, *Paullinia*, and *Ilex* for caffeine; *Crocus*, *Buddleja*, and *Gardenia* for crocins) wherein they presumably evolved through convergent evolution. The closely related *Coffea* and *Gardenia* genera belong to the Rubiaceae family and synthesize, respectively, caffeine and crocins in their fruits.

**Results:**

Here, we report a chromosomal-level genome assembly of *Gardenia jasminoides*, a crocin-producing species, obtained using Oxford Nanopore sequencing and Hi-C technology. Through genomic and functional assays, we completely deciphered for the first time in any plant the dedicated pathway of crocin biosynthesis. Through comparative analyses with *Coffea canephora* and other eudicot genomes, we show that *Coffea* caffeine synthases and the first dedicated gene in the *Gardenia* crocin pathway, *GjCCD4a*, evolved through recent tandem gene duplications in the two different genera, respectively. In contrast, genes encoding later steps of the *Gardenia* crocin pathway, ALDH and UGT, evolved through more ancient gene duplications and were presumably recruited into the crocin biosynthetic pathway only after the evolution of the *GjCCD4a* gene.

**Conclusions:**

This study shows duplication-based divergent evolution within the coffee family (Rubiaceae) of two characteristic secondary metabolic pathways, caffeine and crocin biosynthesis, from a common ancestor that possessed neither complete pathway. These findings provide significant insights on the role of tandem duplications in the evolution of plant specialized metabolism.

## Background

Flowering plants have evolved a diverse array of secondary metabolites to repel pathogens and predators, attract pollinators, and drive ecosystem functions. In many cases, the genomic context for the evolution of specialized plant compounds involves tightly linked clusters of genes, usually containing nonhomologous gene families, that together control novel biosynthetic pathways [1–3]. A few important metabolic clusters involve only tandem duplicates within single gene families, such as the *N*-methyltransferase (*NMT*) genes that control caffeine biosynthesis in the coffee plant [4], and the cytochrome p450 genes encoding the 2,4-dihydroxy-7-methoxy-1,4-benzoxazin-3-one (DIMBOA) metabolic cluster of maize, which produces an important defense compound [5, 6]. Tandem gene duplicate clusters originally arise as copy number variants (CNVs) in populations that later become fixed within species by evolving split or novel functions [7]. Given the ongoing nature of CNV production during evolution, genome sequencing of closely related plants harboring distinct secondary metabolite profiles holds great promise for understanding the stepwise evolution of important tandem duplicate clusters.

**Fig. 1:**
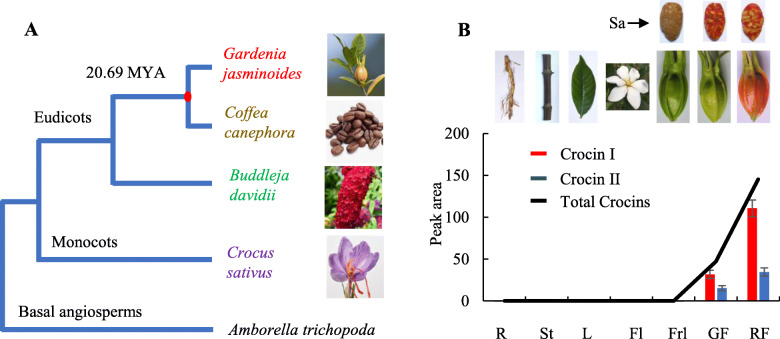
Crocin biosynthesis. **a** Simplified angiosperm phylogenetic tree. Crocin-synthesizing genera are shown in red (*Gardenia*), green (*Buddleja*), and purple (*Crocus*), and caffeine-synthesizing genera (*Coffea*) in brown. The red dot marks the divergence of *Coffea* and *Gardenia*. The divergence time between *G. jasminoides* and *C. canephora* was estimated at approximately 20.69 MYA. **b** Crocin accumulation in different *G. jasminoides* organs. R, root; St, stem; L, leaf; Fl, flower; Frl, fruitlet; GF, green fruit; RF, red fruit; Sa, sarcocarp

The *Gardenia* genus, which is among the most commonly grown horticultural plants worldwide and is valued for the strong, sweet fragrance of its flowers, belongs to the family Rubiaceae. In this large family of angiosperms, only the *Coffea canephora* (robusta coffee) genome has been sequenced to date [4]. The Chinese species *Gardenia jasminoides* (gardenia) has been cultivated for at least 1000 years and was introduced to Europe and America in the mid-eighteenth century. The fruits of *G. jasminoides*, whose major bioactive constituents are genipin and crocins, were used as an imperial dye for royal costumes during the Qin and Han dynasties in China and are recorded in the Chinese Pharmacopoeia [8, 9]. Unlike coffee, gardenia does not accumulate caffeine. However, in a pattern similar to the scattered instances of convergent caffeine biosynthesis among several angiosperm families [4, 10], crocins are found in the flowers of the distantly related plants *Buddleja davidii* (Buddlejaceae) and in the stigmas of *Crocus sativus* (saffron) (Iridaceae) (Fig. 1a).

Apocarotenoids, derived from carotenoids by oxidative cleavage [11–13], play crucial roles in plants as signaling molecules, flower and fruit pigments, and regulators of membrane fluidity [14]. They have been reported to have anticancer, anti-inflammatory, antioxidant, and anti-diabetic activities and to be beneficial in the treatment of central nervous system and cardiovascular diseases [15, 16]. Crocin biosynthesis in saffron stigmas is initiated by carotenoid cleavage dioxygenase 2 (CsCCD2), which cleaves zeaxanthin to produce crocetin dialdehyde [17]. The aldehyde dehydrogenase CsALDH3I1 and the UDP-glucosyltransferase CsUGT74AD1 perform, respectively, the dehydrogenation of crocetin dialdehyde to crocetin and its glycosylation to crocins 1 and 2′ [18]. The UGT mediating the formation of more highly glycosylated crocins is still uncharacterized [18]. In *Buddleja* flowers, only the zeaxanthin cleavage step has been characterized, and it is mediated by BdCCD4.1 and BdCCD4.3 [19]. Thus, it appears that crocin biosynthesis in eudicots (*Buddleja*) and monocots (*Crocus*) has evolved through the convergent evolution of different CCD subclasses (CCD2 and CCD4, respectively) that have acquired the capacity to cleave zeaxanthin at the at the 7/8,7′/8′ positions to produce crocetin dialdehyde.

In *G. jasminoides*, crocins are accumulated in green and red fruits (Fig. 1b). The *Gardenia* crocin biosynthesis pathway has not yet been elucidated, in spite of the availability of transcriptome data [20]. Two *G. jasminoides* UGTs, GjUGT94E5 and GjUGT75L6, are able to catalyze the two-step conversion of crocetin into crocins in vitro [21]. However, the expression profiles of the corresponding genes are not consistent with their proposed role in crocin biosynthesis in *G. jasminoides* fruits [20]. Additionally, the lack of genomic information for crocin-producing species hampers the elucidation of the mechanisms underlying the molecular evolution of crocin biosynthesis. Recently, sequencing of the *C. canephora* (Rubiaceae) and *Camellia sinensis* (Theaceae) genomes has shown that the synthesis of caffeine, a purine alkaloid, has evolved independently in the two genera through tandem duplication and neofunctionalization of different *N*-methyltransferase (NMT) ancestral genes [4].

Here, we report a chromosome-level assembly of the highly heterozygous *G. jasminoides* genome, using a combination of Illumina short reads, Oxford Nanopore (ONT) long reads, and Hi-C scaffolding. The genes involved in the crocin biosynthesis pathway were identified through functional assays, and the molecular evolution of crocin and caffeine biosynthesis in the Rubiaceae family was clarified through comparative genomic studies.

## Results

### Chromosome-level assembly of the *G. jasminoides* genome (Additional file 1)

The size of the *G. jasminoides* genome was predicted to be 550.6 ± 9 Mb (± SD) based on flow cytometry and 547.5 Mb based on 17 *k*-mer distribution analysis and to show a very high level (about 2.2%) of heterozygosity (Additional file 2: Fig. S1). A highly collapsed and fragmented genome was assembled by ALLPATHS-LG using Illumina shotgun reads (293× coverage) (Additional file 2: Table S1). This assembly was 635.6 Mb in size (28% of N bases) and was composed of 58,859 scaffolds (N50, 60.6 kb) (Additional file 2: Table S3).

To improve the contiguity of the assembly, we generated 2,675,530 Oxford Nanopore (ONT) long reads with an N50 of 21.6 kb. The longest reads were 366.8 kb, and the genome coverage was about 60× (Additional file 2: Table S2). After testing different de novo assembly pipelines, we identified a package (Canu-SMARTdenovo-3×Pilon) that yielded satisfactory results (Additional file 2: Table S3). A contiguous assembly of 677.9 Mb with a contig N50 of 703.1 kb was produced, and the longest contig in the assembly was 11.7 Mb. Since the assembly size was larger than predicted genome size, we ran Purge Haplotigs to collapse the highly heterozygous regions. The purged assembly was 534.1 Mb long with a contig N50 of 1.0 Mb (Additional file 2: Table S3). The completeness of the genome assembly was assessed with the BUSCO pipeline, which found 95.0% complete BUSCOs, of which 92.7% were single-copy and 2.3% duplicated (Additional file 2: Table S3).

**Fig. 2:**
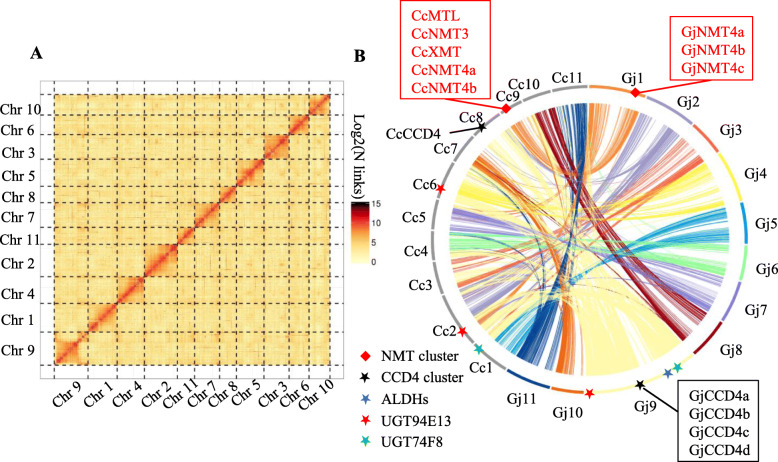
The *G. jasminoides* genome. **a** Chromosome-level assembly of the *G. jasminoides* genome using Hi-C technology. **b** Synteny between *C. canephora* and *G. jasminoides* chromosomes. The positions of *NMT* genes, catalyzing caffeine biosynthesis in *Coffea*, and of *CCD*, *ALDH*, and *UGT* genes, catalyzing crocin biosynthesis in *Gardenia*, are marked

The contiguity of the assembly was further improved using Hi-C scaffolding. There were 56,933,122 paired-end reads representing valid interactions were used to scaffold 99.5% of the assembly into 11 chromosomes using the Lachesis package [22] (Fig. 2a). The final corrected chromosome-level genome was 535 Mb in size, 531 of which were assembled in the 11 chromosomes (Table 1). The *G. jasminoides* chromosomes showed a significant level of synteny with their *C. canephora* counterparts, with a limited number of translocations between the two genomes (Fig. 2b, Additional file 2: Fig. S2). In addition, a 154,919-bp chloroplast genome and 640,334-bp mitochondrial genome of *G. jasminoides* were assembled and identified.

**Table 1:** Metrics of the final assembly and annotation of the *G. jasminoides* genome

**Assembly size**	535 Mb
**N50**	44 Mb
**No. of chromosomes**	11
**Assembly in chromosomes**	531 Mb
**Assembly in unanchored contigs**	4 Mb
**BUSCOs in assembly**	95.8% (S, 92.7%; D, 2.3%; F, 0.8%)
**No. of genes**	35,967
**No. of genes in chromosomes**	35,779
**No. of genes in unanchored contigs**	188
**BUSCOs in annotation**	96.5% (S, 87.9%; D, 2.1%; F, 6.5%)

### Genome annotation and phylogenetic analysis

The *G. jasminoides* genome comprises 35,967 protein-coding genes (Table 1). Consistent with the assessment of genome assembly quality, orthologs of 96.5% of eukaryotic BUSCOs were identified in the *G. jasminoides* gene sets (Table 1). Transposable elements (TEs) account for approximately 54.0% (288,723,343 nt) of the *G. jasminoides* genome (Additional file 2: Table S4, 5, 6), and 62.2% of these TEs are long terminal repeat (LTR) elements. We identified 1798 full-length LTR elements including 709 Gypsy and 403 Copia elements. These elements have an average insertion time of 1.4 million years ago (MYA) assuming a mutation rate of *μ* = 1.3 × 10^−8^ (per bp per year) [23, 24] (Additional file 2: Fig. S3), and *Gypsy* elements are much younger than *Copia* elements (1.2 MYA vs 1.7 MYA, *P* < 0.05). Most 5s rRNAs are tandemly arrayed in Chr 11 of the *G. jasminoides* genome, and the tandem repeats of 18s rRNAs (average size of 1.8 kb), 5.8s rRNA (average size of 154 bp), and 28s rRNAs (average size of 6.3 kb) were clustered in Gardenia 10, spanning 65.9 kb (Additional file 2: Table S7, 8).

34,662 orthologous gene groups were found for *G. jasminoides* and 10 additional angiosperms, covering 335,254 genes in total. Among these, 155,220 genes clustering into 6671 groups were conserved in all plants examined, and 1248 gene families containing 5123 genes appeared to be unique to *G. jasminoides* (Additional file 2: Fig. S4A). Eight hundred ninety-one gene families were expanded, and 2666 gene families were contracted in the *G. jasminoides* lineage (Additional file 2: Fig. S4A). *G. jasminoides*-specific and expanded gene families comprised glycoside hydrolases (PF00232) and UDP-glucosyl/glucuronosyl transferases (PF00201), which might be related to the metabolism of bioactive compounds such as crocins and geniposides (Additional file 2: Table S9). Based on phylogenomic analysis of 121 single-copy genes, the divergence time between *G. jasminoides* and *C. canephora* was estimated at approximately 20.7 MYA (Fig. 1a, Additional file 2: Fig. S4B).

### Identification of *G. jasminoides* candidate crocin biosynthetic genes (Additional file 3)

The mature fruit of *G. jasminoides* exhibits a visible reddish yellow or brown color, due to the presence of crocins, crocetin, and geniposides [21, 25, 26], which are found in the mature pericarp and sarcocarp of the fruit (Fig. 1b). We collected samples from three different fruit developmental stages: fruitlet with a green pericarp and immature sarcocarp, green fruit with a green pericarp and red sarcocarp, and red fruit with red pericarp and red sarcocarp. Analysis of different organs and tissues, including the root, stem, leaf, flower, fruitlet, green fruit, and red fruit, indicated that crocins are specifically localized in green and red fruits, whose sarcocarps have matured (Fig. 1b, Additional file 2: Fig. S5-S6). Transcriptome reads from seven *G. jasminoides* organs were mapped to the assembled genome and annotated gene loci to calculate gene expression (fragments per kilobase of exon per million reads mapped, FPKM). Genome-wide analysis of the *G. jasminoides* assembly identified fourteen *CCD* genes that might be involved in the tailoring of carotenoids for apocarotenoid formation (Additional file 2: Fig. S7, Additional file 2: Table S10). The *GjCCD4* and *GjCCD8* gene subfamilies were expanded in the *G. jasminoides* lineage (Additional file 2: Table S10). *GjCCD4a* was highly expressed in green and red fruits (FPKM > 1000), in accordance with the distribution of crocins, while *GjCCD4a*, *GjCCD4c*, and *GjCCD4d* were highly expressed in flowers (Additional file 2: Fig. S10, Additional file 2: Table S11). GjCCD4a (ARU08109.1) showed high amino acid sequence similarity to GjCCD4b (81%), GjCCD4c (81%), and GjCCD4d (77%), and the four genes were located on a single gene cluster on chr 9 (see below).

Eighteen *G. jasminoides* genes with similarity to aldehyde dehydrogenases (ALDHs), which catalyze the oxidation of aldehydes [27], were identified and classified into 10 distinct subfamilies (Additional file 2: Fig. S8, Additional file 2: Table S12). In *C. sativus*, CsALDH3I1 is known to mediate with high efficiency the dehydrogenation of crocetin dialdehyde to crocetin [18]; however, expression of *GjALDH3* genes was low in the fruit and thus inconsistent with the crocin distribution in *G. jasminoides* organs. Instead, *GjALDH2C3* (KY631926.1) was highly expressed in green and red fruits and flowers (Additional file 2: Fig. S10, Additional file 2: Table S12).

Two hundred thirty-seven *UGT* genes were identified in the *G. jasminoides* genome and were classified into 19 subfamilies (Additional file 2: Fig. S9, Additional file 2: Table S13). More than 50% are members of the *UGT79*, *UGT73*, *UGT85*, and *UGT94* groups. Eleven *UGT* genes were significantly expressed in green and red fruits (Fig. S10, Additional file 2: Table S13).

### Elucidation of the *G. jasminoides* crocin biosynthetic pathway

**Fig. 3:**
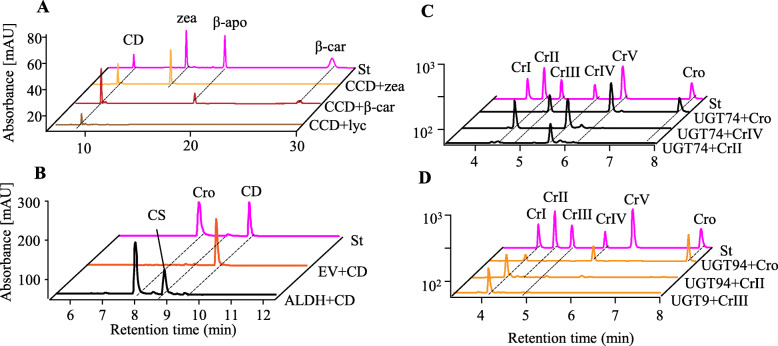
Elucidation of the crocin biosynthesis pathway in *G. jasminoides.***a** UPLC-DAD chromatograms (abs at 440 nm) of *E. coli* extracts expressing GjCCD4a. **b**–**d** UPLC-DAD chromatograms (abs at 440 nm) of in vitro reactions catalyzed by GjALDH2C3 (**b**), GjUGT74F8 (**c**), and GjUGT94E13 (**d**). St, standards; EV, empty vector; lyc, lycopene; β-car, β-carotene; zea, zeaxanthin; cro, crocetin; CD, crocetin dialdehyde; CS, crocetin semialdehyde; CrI–V, crocins I–V

We used expression in *E. coli* to test the activity of the candidate crocin biosynthetic genes identified by expression analysis. A strain co-transformed with pET32a-*CCD4a* and the zeaxanthin accumulation plasmid pACCAR25ΔcrtX showed discoloration, and a new product with a retention time and characteristic fragment ions ([M + H]^+^: *m/z* 297.1939) identical to that of the crocetin dialdehyde standard was observed (Fig. 3a, Additional file 2: Fig. S11).

In both *Crocus* and *Buddleja*, the substrate for crocetin dialdehyde formation is zeaxanthin [17, 19, 28] (Fig. 1b). Since lycopene and β-carotene are also possible substrates, we expressed GjCCD4a in *E. coli* strains accumulating these carotenoids. In both cases, we detected the formation of crocetin dialdehyde (Fig. 3a) and, in the case of β-carotene, also of the intermediate product 8′-apo-β-carotenal (Fig. 3a, Additional file 2: Fig. S12, Additional file 2: Table S14). In contrast, none of the other *Gardenia* or *Coffea* CCD4 proteins was able to cleave zeaxanthin or β-carotene (Additional file 2: Fig. S13). Protein structure modeling and docking analysis of GjCCD4a suggested the capacity to bind all three substrates, in accordance with its catalytic activity (Additional file 2: Fig. S14).

Next, we tested the ability of the purified GjALDH2C3 protein to catalyze in vitro the oxidation of crocetin dialdehyde. Incubation of crocetin dialdehyde with GjALDH2C3 yielded two new peaks, and the retention time (7.84 min) and characteristic fragment ions ([M + H]^+^: *m/z* 327.1605) of the most abundant peak were consistent with those of the crocetin standard. In addition, the intermediate product crocetin semialdehyde (8.68 min and [M + H]^+^: *m/z* 311.1686) was also detected (Fig. 3b, Additional file 2: Fig. S15).

As noted above, a large number of *UGT* genes are expressed in *Gardenia* fruits, and these are likely involved in the synthesis of several classes of glycosylated compounds in these organs. The enzymatic activities of twelve candidate UGTs were characterized in vitro using crocetin as a substrate (marked with an asterisk in Additional file 2: Table S13). Three UGTs, namely GjUGT74F8, GjUGT75L6, and GjUGT94E13, were able to glycosylate crocetin and/or crocins (Fig. 3c, d, Additional file 2: Fig. S16-S21). Of these, only two (GjUGT74F8 and GjUGT94E13) were expressed at high levels in fruits, suggesting that they are involved in crocin production, while the third, described previously [21], was not significantly expressed in these organs (Additional file 2: Fig. S10).

GjUGT74F8, which is the most highly expressed among all UGTs (Additional file 2: Fig. S10, Additional file 2: Table S13), is similar to CsUGT74AD1, which is responsible for crocin primary glycosylation in *C. sativus* [18, 29] (Additional file 2: Fig. S22). Upon in vitro incubation of GjUGT74F8 with crocetin, two new peaks with retention times of 4.96 and 6.30 min were detected. The newly formed products had the same fragmentation patterns as crocin III ([M + Na]^+^, 675.2620) and crocin V ([M + Na]^+^, 513.2090), respectively, demonstrating that GjUGT74F8 has the ability to add one or two β-d-glucoses to the carboxyl groups of crocetin (primary glycosylation) (Fig. 3c, Additional file 2: Fig. S20). In addition, reversible conversion of crocin II to crocin IV and vice versa was observed, suggesting that GjUGT74F8 possesses also a hydrolytic activity, able to remove a single β-d-glucose (Fig. 3c, Additional file 2: Fig. S20).

In vitro incubation of crocetin with purified GjUGT94E13 resulted in the generation of two new products with retention times of 4.22 and 5.75 min, respectively. These two compounds had mass spectra with [M + Na]^+^ peaks at *m/z* 999.3699 and 675.2610, respectively, consistent with those of crocin I and crocin IV (Fig. 3d, Additional file 2: Fig. S16). We also found that crocin IV was gradually converted to crocin I by the extension of the incubation time with GjUGT94E13 (Fig. 3d, Additional file 2: Fig. S16) and that GjUGT94E13 could efficiently catalyze the glycosylation of crocins II and III to crocin I (Fig. 3d, Additional file 2: Fig. S17, S18). In no case was generation of a crocin with a single β-d-glucosyl ester detected under different reaction conditions, suggesting that GjUGT94E13 could catalyze either the addition of a second glucosyl group to a pre-existing one (secondary glycosylation) or the sequential addition of two glucosyl groups to a carboxyl group (primary and secondary glycosylation).

**Fig. 4:**
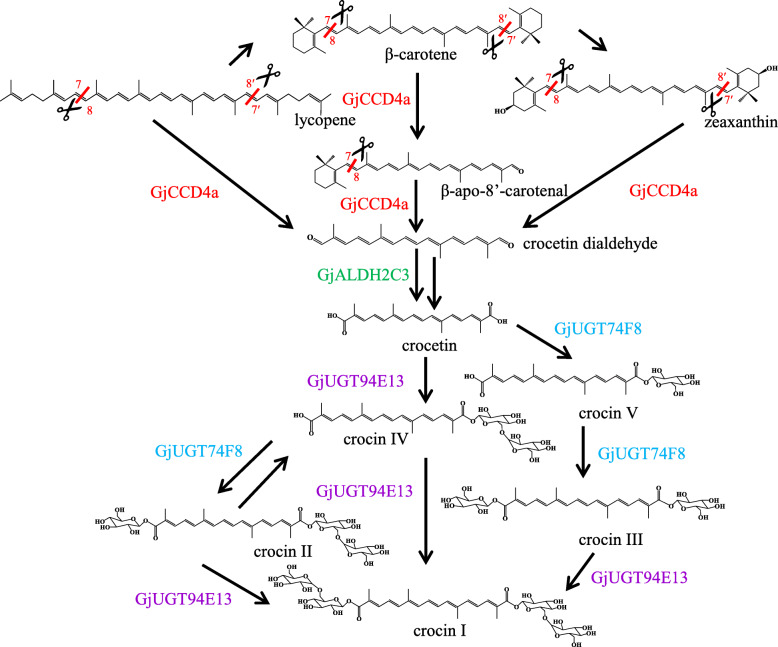
The crocin biosynthesis pathway in *G. jasminoides*

Based on these results, the complete crocin biosynthesis pathway in *G. jasminoides* is depicted in Fig. 4. Lycopene, β-carotene, and zeaxanthin are cleaved at the 7/8,7′/8′ positions by GjCCD4a, yielding crocetin dialdehyde, which is then converted to crocetin by GjADH2C3. Crocetin is the substrate of two UGTs, GJUGT74F8 and GjUGT94E13, adding respectively one or two glucose esters to the two carboxylic groups.

### Evolution of crocin and caffeine biosynthesis genes in the Rubiaceae

**Fig. 5:**
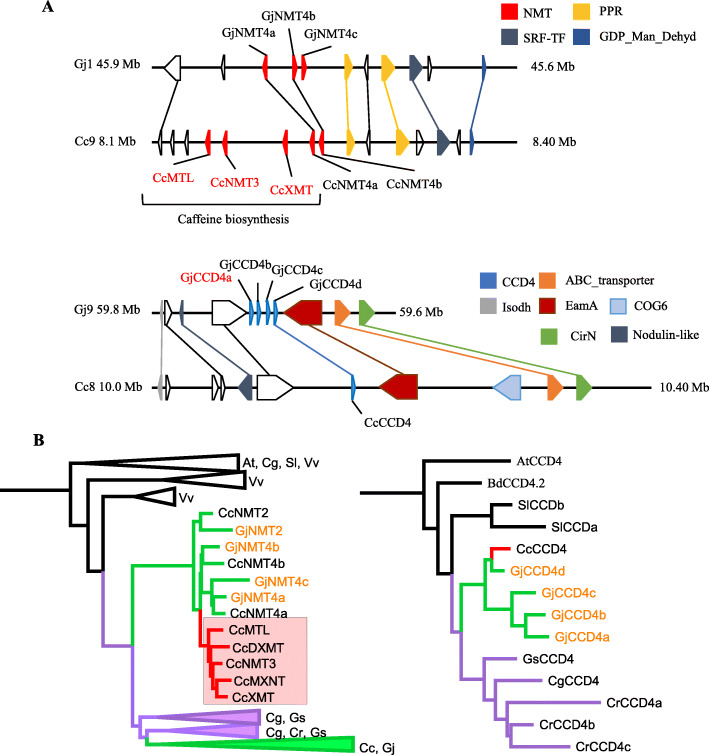
*Coffea* caffeine synthases and *Gardenia* crocin biosynthesis dioxygenase arose through genus-specific tandem duplications. **a** Microsynteny between *Coffea* and *Gardenia* around the caffeine synthases of the former and the first dedicated gene in crocin biosynthesis in *Gardenia*, *GjCCD4a*. **b** Simplified phylogenies (pruned from the trees provided in Figs. S27-S28) depicting the evolution of caffeine and crocin biosynthetic genes in Gentianales. Purple branches indicate clades that are Gentianales-specific; green, Rubiaceae-specific; red, *Coffea*-specific. The gene IDs used for the phylogenetic trees of NMTs and CCD are the following: CcNMT2 (Cc02_g09350), CcNMT4b (Cc09_g07000), CcNMT4a (Cc09_g06990), CcMTL (Cc09_g06950), CcNMT3 (Cc09_g06960), CcXMT (Cc09_g06970), CcDXMT (Cc01_g00720), CcMXMT (Cc00_g24720), GjNMT2 (Gj9A1032T108), GjNMT4a (Gj1A458T26), GjNMT4b (Gj1X458T25), GjNMT4c (Gj1A458T28), SlCCD4a (Solyc08g075480.2.1), SlCCDb (Solyc08g075490.2.1), CcCCD4 (Cc08_g05610), GjCCD4a (Gj9A597T69), GjCCD4b (Gj9A597T68), GjCCD4c (Gj9A597T67), GjCCD4d (Gj9P597T6), GsCCD4 (Gs_scaff312_1.38), CrCCD4a (CRO_T140281), CrCCD4b (CRO_T140282), CrCCD4c (CRO_T140277), and CgCCD4 (cal_g006885.t1). Gene IDs for those with the biochemical activities in question are shown in red. *Gardenia* gene IDs are shown in orange. All other gene IDs are shown in black. For the collapsed tree branches in the pruned trees, species abbreviations are as follows: At, *Arabidopsis thaliana*; Vv, *Vitis vinifera*; Sl, *Solanum lycopersicum*; Gs, *Gelsemium sempervirens*; Cr, *Catharanthus roseus*; Cg, *Calotropis gigantea*; Cc, *Coffea canephora*; Gj, *Gardenia jasminoides*; Bd, *Buddleja davidii*

Synteny analysis demonstrated clearly that *Coffea* caffeine synthases and *Gardenia CCD4a* are localized, respectively, in tandemly repeated arrays that are unique to each of the two species, suggesting the two gene duplication series occurred after the *Coffea*-*Gardenia* divergence (Fig. 5a, Additional file 2: Fig. S23-24). The *Coffea* caffeine synthase gene cluster is syntenic to non-caffeine-producing NMTs in the *Gardenia* genome (Fig. 5a, Additional file 2: Fig. S23), while the close Gentianales relative *Gelsemium* (Gelsemiaceae) does not even contain NMTs in the homologous chromosomal regions. The latter observation suggests that the common ancestral genomic block in all gentianalean species lacked an *NMT* gene and that the ancestor of the future caffeine synthase cluster translocated there during Rubiaceae evolution, where it duplicated in *Coffea* only after the divergence of *Coffea* and *Gardenia* (Fig. 5b, Additional file 2: Fig. S24). Conversely, the *Gardenia* gene cluster comprising *GjCCD4a-b-c-d* is syntenic to a unique *CCD4* gene in the coffee genome (Fig. 5a, Additional file 2: Table S15), yielding an inverted evolutionary scenario wherein local duplications in *Gardenia* that occurred after *Coffea*-*Gardenia* common ancestry led to the production of distinct bioactive compounds. In contrast, the downstream crocin biosynthesis genes *GjALDH2C3*, *GjUGT74F8*, and *GjUGT94E5* are localized in gene clusters that appear conserved between *Coffea* and *Gardenia* (Additional file 2: Fig. S24), suggesting that they may have been generated before the split of the two genera.

Phylogenetic analyses of NMTs related to caffeine synthases and CCDs related to crocin synthase confirmed this evidence based on synteny. Caffeine biosynthetic enzymes belong to a lineage of NMTs that are specific to Rubiaceae, however, ones that duplicated and specifically evolved caffeine biosynthetic function only in *Coffea* (Fig. 5b, Additional file 2: Fig. S26, Additional file 4: Fig. S27). Similarly, the CCD4 subfamily wherein *Gardenia*’s crocin synthase resides is Rubiaceae-specific, but not tandemly duplicated in *Coffea* as it is in *Gardenia* (Fig. 5b, Additional file 5: Fig. S28). The *UGT74* and *UGT94* gene subfamilies in both Rubiaceae species are contained within distinct subclades in a large UGT family tree, each grouping with related genes from other Gentianales in sublineages likely to be of ancient origin (Additional file 6: Fig. S29). Similarly, the ALDH2 genes of *Coffea* and *Gardenia* are close relatives in a subclade also comprising genes from other Gentianales species (Additional file 7: Fig. S30).

## Discussion

### Chromosome-level assembly of a highly heterozygous genome

Many plants including medicinal ones such as *Salvia miltiorrhiza* [30], *Panax ginseng* [31], *Panax notoginseng* [32, 33], and *Glycyrrhiza uralensis* [34] are highly heterozygous, resulting in fragmented genome assemblies when using traditional short read methods. *G. jasminoides*, the second plant of Rubiaceae with a sequenced genome, is self-incompatible with high (2.2%) heterozygosity. We developed an efficient method to produce chromosome-level assemblies for heterozygous plant genomes using a combination of short Illumina and long ONT reads and Hi-C scaffolding [22, 35, 36]. The assemblies constructed using short reads were extremely fragmented, while the combination of short and ONT reads with a Canu-SMARTdenovo assembly pipeline produced a *G. jasminoides* assembly with the highest contiguity. The significant difference in the number of annotated repeat sequences between the ONT (53.97%) and Illumina (36.51%)-based assemblies indicates the importance of genome contiguity for annotation of repetitive elements (Additional file 2: Table S3). However, the ONT-based assembly size was much larger than the genome size predicted by *k*-mer distribution and flow cytometry, suggesting that highly divergent haplotypes were assembled separately. This was confirmed and corrected by haplotig purging, whereafter the assembly was scaffolded into chromosomal pseudomolecules using Hi-C. Given the low cost of ONT long reads, the pipeline described here provides an extremely cost-effective method for producing chromosome-level assemblies of highly heterozygous plant genomes.

### Characterization of the *Gardenia* crocin biosynthetic pathway

In a previous transcriptomic study [20], we identified several *G. jasminoides* unigenes bearing high sequence similarity to candidate genes for crocin biosynthesis (Additional file 2: Table S16). However, the low number of tissues analyzed by RNA-Seq (3 versus 7 in the present study) and the high level of fragmentation of the unigenes prevented the unambiguous identification of bona fide candidates for all the steps through co-expression analysis, as well as the expression in *E. coli* of the corresponding full-length proteins for functional assays. Based on genome-wide candidate gene identification, expression studies, and in vitro functional studies, three of the 7 previously identified unigenes (*GjCCD4a*, *GjALDH2C3*, and *UGT94E13*), but also a novel *UGT74F8* gene, which went undetected in the previous transcriptome analysis, were shown to be involved in crocin biosynthesis in *G. jasminoides*.

In *C. sativus*, zeaxanthin is cleaved symmetrically at the 7/8,7′/8′ positions by CsCCD2 to produce crocetin dialdehyde [17, 28], while, in *B. davidii*, the same reaction is carried out by BdCCD4.1 and BdCCD4.3 [19]; no activity of CsCCD2 and/or BdCCD4.1/BdCCD4.3 on other carotenoids, including β-carotene and lycopene, has been reported. GjCCD4a from *G. jasminoides* shares only 31% identity with CsCCD2, but 56 and 59% identity with BdCCD4.1 and BdCCD4.3, respectively, and can catalyze the symmetric 7/8,7′/8′ cleavage of zeaxanthin, β-carotene, and lycopene to produce crocetin dialdehyde. Thus, crocetin biosynthesis evolved in different plant taxa through convergent evolution, since the carotenoid cleavage step is catalyzed by a CCD2 in *Crocus* [17], but by CCD4 enzymes in *Buddleja* [19] and *Gardenia* (this paper). Convergent evolution also underlies the second step of crocin biosynthesis, which is mediated by an ALDH3 in *C. sativus* [18] and by an ALDH2 in *G. jasminoides* (this paper).

Two *Gardenia* UGTs, GjUGT75L6 and GjUGT94E5, were previously shown to catalyze the conversion of crocetin to crocins in vitro [21]. However, their low expression in fruits suggests that they are not the primary enzymes involved in crocin biosynthesis in vivo. We identified two novel *UGT* genes, *GjUGT74F8* and *GjUGT94E13*, closely related to *GjUGT75L6* and *GjUGT94E5* that are highly expressed in fruits and show co-expression with *GjCCD4a*. *GjUGT74F8* was the most highly expressed *UGT* gene in mature fruit, and the protein product catalyzed the primary glycosylation of crocetin, the same reaction catalyzed by GjUGT75L6 [21] and by *Crocus* UGT74AD1 [18]. Similar to the latter, GjUGT74F8 did not exhibit a secondary glycosylation activity. GjUGT74F8 was also found to catalyze de-glycosylation reactions. Reversible glycosylation has been previously described in other plant UGTs [37]. GjUGT94E13 catalyzed primary and secondary glycosylation of crocetin to form crocins IV and I, presumably via the sequential addition of two β-d-glucosyl esters. Crocins V and II were undetectable when crocetin was incubated with GjUGT94E13, suggesting that the secondary glycosylation step occurred much faster than the primary glycosylation one. Incubation of crocin II or crocin III with GjUGT94E13 resulted in the complete conversion to crocin I, confirming that GjUGT94E13 is able to add a second glucosyl moiety to a mono-glycosyl group (secondary glycosylation).

### Molecular evolution of caffeine and crocin biosynthesis in the Rubiaceae

The chromosome-level *Gardenia* genome assembly reported here and its comparative analysis with the closely related *Coffea* genome [4] allowed a detailed reconstruction of the molecular events that led to the evolution of the caffeine and crocin pathways in the two genera. The *NMT* gene cluster involved in caffeine biosynthesis in *C. canephora* shows synteny to a region in *G. jasminoides* that also contains NMTs, but ones that predate the evolution of the caffeine synthase cluster characteristic of coffee. Conversely, the first dedicated gene in crocin biosynthesis, *GjCCD4a*, is part of a 4-gene cluster for which, in coffee, there is only one ortholog (*Cc08g05610*), showing the highest identity (80.8%) to *GjCCD4c*. The two genes are highly expressed in flowers in the two species, suggesting that they may be responsible for carotenoid cleavage in the white flowers of *G. jasminoides* and *C. canephora*, followed by volatile apocarotenoid formation [38]. Additionally, neither the CcCCD4 nor the GjCCD4b-c proteins exhibit 7/8,7′/8′ cleavage activity against any carotenoid tested. These findings suggest that caffeine and crocetin biosynthesis in coffee and *Gardenia* evolved, respectively, through tandem duplications and functional specialization of *NMT* and *CCD4* genes, and that these tandem duplications occurred after the separation of the two genera. Besides giving rise to novel metabolic functions, tandem gene duplications play several additional roles in genome evolution; for instance, in the birch and avocado genomes, tandem duplicates are enriched for pathogen responses [7, 39], while in the Australian carnivorous pitcher plant, tandem duplicates are enriched in enzymatic functions related to the acquisition of carnivory [40]. The production of tandem duplicates as copy number variants in populations, and their subsequent species-level fixation, provides a general evolutionary substrate for novel secondary metabolic activities in the recent adaptive landscapes of plant genomes.

## Conclusion

This study sequenced the genome of *G. jasminoides*, a crocin-producing species, and dissected the complete crocin biosynthetic pathway through genomic and functional assays. Comparative analyses with *C. canephora* revealed that the caffeine biosynthetic genes (NMTs) in *Coffea* and the first dedicated crocin biosynthetic gene (GjCCD4a) in *Gardenia* evolved through recent tandem gene duplications in the two different genera, respectively. This study highlighted the divergent evolution of caffeine and crocin biosynthesis within the coffee family, providing significant insights on the role of tandem duplications in the evolution of plant specialized metabolism.

## Methods

### Plant materials

An individual *G. jasminoides* (line 1–9) plant, which was asexually propagated by cutting, was obtained from Nanchuan District (29° N and 107° E), Chongqing City, China. Seven independent organs from *G. jasminoides*, including the root, stem, leaf, flower, fruitlet, green fruit, and red fruit, were collected. The fruitlet, green fruit, and red fruit represented different maturity. In total, 21 samples including three biological replicates for each organ were gathered. All samples were divided into two portions, which were used for the measurement of crocin content and RNA sequencing. The pooled young leaves were used to DNA extraction for Illumina and ONT sequencing.

### ONT sequencing and assembly

Following the methods for megabase-size DNA preparation, we extracted the high molecular weight (HMW) genomic DNA of *G. jasminoides*, which was used to construct paired-end, mate-pair, and ONT libraries. The HMW gDNA was randomly fragmented using a Megaruptor; then, the large fragments were selected and purified using BluePippin and AMPure beads. After end-prep, ligation of sequencing adapters, and tether attachment, the fragments were sequenced on the ONT GridION X5 platform with 6 nanopore flow cells (v9.4.1). Base calling was performed using the Oxford Nanopore base caller Guppy (v1.8.5). Canu (v1.7) was used to correct, trim, and assemble the ONT raw reads with the default parameters [41]. The correction-free approach, named Minimap/miniasm [42], was also independently performed with the recommended parameters. The assembler SMARTdenovo was also used for assembly with the corrected and trimmed ONT reads as input [43]. The Canu-SMARTdenovo contigs were polished three times with Pilon (v1.22) using Illumina short reads [44]. The final scaffolds were constructed with the polished contigs and corrected ONT reads using SSPACE-LongRead (version 1.1) [45], and heterozygous sequences were removed using Purge Haplotigs [46]. The quality of the genome assembly was estimated by searching for Benchmarking Universal Single-Copy Orthologs (BUSCO v4.0, embryophyte profile) with Embryophya *odb* 10 dataset [47]. Illumina sequences from the *G. jasminoides* DNA and RNA libraries were mapped to evaluate the quality of the assembled genome using BWA (Burrows-Wheeler Aligner) [48].

### Chromosome construction using Hi-C

Fresh tissue of *G. jasminoides* was used to construct a Hi-C sequencing library. Steps included chromatin crosslinking, chromatin digestion with Hind III, biotin labeling and end repair, DNA purification, streptavidin pull-down of labeled Hi-C ligation products, and construction of an Illumina sequencing library. The clean sequences were mapped to the draft genome, and valid Hi-C reads were used to correct the draft assembly. Then, the draft genome of *G. jasminoides* was assembled into chromosomes (2n = 22) using Lachesis [22].

### Genome annotation and RNA-Seq analysis

Annotation of structural repeats in the *G. jasminoides* genome was performed using the RepeatModeler (http://www.repeatmasker.org/RepeatModeler/; v1.0.9) package, which combines RECON and RepeatScout to identify and classify the repeat elements. The long terminal repeat retrotransposons (LTR-RTs) in *G. jasminoides* were identified using LTR_Finder (v1.0.6) and LTR_retriever with the default parameters [49]. The repeat sequences were masked by RepeatMasker (v4.0.6) (http://www.repeatmasker.org/).

RNA-Seq on the HiSeq 4000 platform was performed for 21 samples. The short reads were assembled de novo using Trinity (v 2.2.0) [50], and peptide sequences were predicted with TransDecoder (v2.1.0) (https://github.com/TransDecoder). The masked *G. jasminoides* genome annotation was ab initio predicted using the MAKER (v2.31.9) [51] annotation pipeline, integrating the assembled transcripts of *G. jasminoides* and protein sequences from *G. jasminoides*, *C. canephora*, and *A. thaliana*. Noncoding RNAs were annotated by aligning to the Rfam database using INFERNAL (v1.1.2) [52], and miRNAs were further analyzed by performing BLASTN searches against the miRNA database. The RNA-Seq reads from different *G. jasminoides* organs were aligned to the masked genome using HiSAT2 (v2.0.5), and the FPKM values of annotated genes in the reference genome were calculated using Cufflinks (v2.2.1) [53].

The amino acid sequences of proteins from *G. jasminoides* and nine other angiosperms were clustered into orthologous groups using OrthoMCL (version 2.0.9) [54]. Phylogenetic analyses of single-copy orthologous genes were performed using the RAxML package (version 8.1.13) using the JTT+G+I substitution model for amino acid sequences with 1000 bootstrap replicates [55]. Divergence times were directly estimated based on the divergence times of *P. trichocarpa*-*G. max* (94–127 MYA) and *B. distachyon*-*Z. mays* (40–53 MYA) obtained from TimeTree (http://www.timetree.org). The Markov Cluster Algorithm (MCL) was used to identify species-specific gene groups [56]. CAFÉ (version 3.1) was used to predict gene family expansion and contraction [57]. Genome synteny analyses were performed using the CoGe web suite, www.genomevolution.org, according to methods described elsewhere [58–60].

### Identification of gene families related to crocin biosynthesis

Protein sequences of the CCD, ALDH, and UGT family members in *A. thaliana* were downloaded from the TAIR database, then were used as queries in BLASTP searches against the *G. jasminoides* protein sequences to identify homologous sequences. Full-length protein sequences were corrected and aligned with ClustalW2 [61]. Phylogenetic trees were constructed using the maximum likelihood method with the Jones-Taylor-Thornton (JTT) model and 1000 Bootstrap replicates [62]. Further analyses incorporated blast searches (using *Gardenia* proteins as queries) of a number of other genomes to identify more CCD, ALDH, and UGT genes. For NMTs, the *Coffea canephora* XMT protein was used as a query (NCBI accession ABD90685.1). Species considered were *Gardenia jasminoides* (CoGe genome ID 53980), *Coffea canephora* (CoGe genome ID 19443), *Arabidopsis thaliana* (CoGe genome ID 16911), *Calotropis gigantea* (CoGe genome ID 36623), *Catharanthus roseus* (CoGe genome ID 36703), *Vitis vinifera* (CoGe genome ID 19990), *Gelsemium sempervirens* (CoGe genome ID 53941), and *Solanum lycopersicum* (CoGe genome ID 12289). Gene model IDs from the respective CoGe-uploaded genomes were retained as leaf IDs for phylogenetic analysis, with the exception that “:” when it appeared in a gene model ID was replaced by “_”. Several additional anchoring protein sequences from NCBI were incorporated in the NMT tree (MTL,_AFV60456.1; DXMT,_ABD90686.1; MXMT,_AFV60445.1; XMT,_ABD90685.1). Searches were run on the CoGe platform using default parameters and saving 100 Blast HSPs per species. Unique translated sequences were then downloaded, duplicates were excluded using BBedit, sequences with internal stop codons were excluded, and then trees were run using PASTA [63] with MAFFT [64] to align the protein sequences and FastTree [65] to create an approximately maximum likelihood tree. Trees were visualized and edited using FigTree (http://tree.bio.ed.ac.uk/software/figtree/) (Additional file 4: Fig. S27, Additional file 5: Fig. S28, Additional file 6: Fig. S29, Additional file 7: Fig. S30). To interpret the supplemental figures, pink branches represent gentianalean clades, green branches represent Rubiaceae clades, and orange gene model IDs represent *Gardenia* genes. Coffee-specific clades are shown in red. In the NMT supplemental tree (Additional file 4: Fig. S27), the anchoring protein sequences are shown in red.

### Enzymatic activity assays and LC/LC-MS analyses

The cDNAs of candidate genes from the *CCD*, *ALDH*, and *UGT* families were de novo synthesized and cloned into expression vectors via digestion and ligation (Additional file 2: Fig. S25). The GjALDH2C3, GjUGT94E13, and GjUGT74F8 proteins were purified from *E. coli* using affinity chromatography to a purity > 95% (Additional file 2: Fig. S15, S16, S19). The *in bacterio* and in vitro activity assays and detailed reaction mixtures are described in the Supplemental information. Crocetin dialdehyde (trans-crocetin dialdehyde) and crocetin (trans-crocetin) were purchased from Sigma-Aldrich (USA) and CFW Laboratories (Germany), respectively. Crocin I and crocin II were purchased from Meilunbio (China). All the chemical reagents used here were of analytical grade.

Samples were analyzed using a Thermo Ultimate 3000 system equipped with an Acquity UPLC® BEH C18 column (1.7 μm, 100 × 2.1 mm). A gradient elution procedure was applied, using the mobile phases acetonitrile containing 0.1% formic acid (A) and water containing 0.1% formic acid (B). The following gradient elution program was used at a flow rate 0.3 mL/min: 0–5 min, 10% A linearly increased to 50% A; 5–8 min, 50% A linearly increased to 90% A; 8–10 min, 90% A linearly increased to 100% A and sustained for 20 min; and 30–31 min, back to 10% A.

Qualitative analysis of each compound was carried out using liquid chromatography-mass spectrometry (Agilent Technologies 1290 Infinity II LC System and 6545 Q-TOF, with Dual Agilent Jet Stream Electrospray Ionization sources). The drying gas was set at 325 °C with a flow rate of 6 L/min, and the sheath gas was set at 350 °C, with a flow rate of 12.0 L/min. The nebulizer was set at 45 psig, and the VCap was set at 4000 V. The data were analyzed using MassHunter (version B.07.00). The detailed information of mentioned compounds was listed in Additional file 2: Table S17, S18.

Nuclear magnetic resonance (NMR) experiments were performed on Bruker AV III 600 NMR spectrometer (600 MHz for ^1^H NMR and 150 MHz for ^13^C NMR) in CDCl_3_ (Sigma-Aldrich, USA), and the chemical shifts were given in *δ* (ppm) with TMS as the internal standard.

## Supplementary information

**Additional file 1.** Detailed methods and results for genome sequencing.

**Additional file 2: Figures S1-S26**; **Tables S1-S18**.

**Additional file 3.** Detailed methods and results for crocin biosynthesis.

**Additional file 4: Figure S27.** Pasta phylogenetic tree of NMTs from different species.

**Additional file 5: Figure S28.** Pasta phylogenetic tree of CCDs from different species.

**Additional file 6: Figure S29.** Pasta phylogenetic tree of UGTs from different species.

**Additional file 7: Figure S30.** Pasta phylogenetic tree of ALDHs from different species.

## Data Availability

*G. jasminoides* line 1–9 and the constructs used in this work can be obtained by writing to K.H. (email: 402632144@qq.com). Genome sequence and assembly data were submitted to the Sequence Read Archive from the NCBI under the accession number PRJNA477438 [66]. The assembled genomes including nuclear genome, chloroplast genome, and mitochondrial genome for *G. jasminoides* were submitted to the NCBI genome resource with accession no. VZDL00000000 [67], and the CoGe with id53980 [68], id55476 [69], and id55477 [70], respectively.
