# Supplementary material for: Tandem gene duplications drive divergent evolution of caffeine and crocin biosynthetic pathways in plants
Source: BMC Biol. 2020 Jun 18;18:63. doi: 10.1186/s12915-020-00795-3 (PMC7302004; doi:10.1186/s12915-020-00795-3)
Supplement: Supplementary file 1 — Additional file 1. Detailed methods and results for genome sequencing. [file 12915_2020_795_MOESM1_ESM.docx]

**Additional file 1: Detailed methods and results for genome sequencing**

*Plant materials.* An individual *G. jasminoides* (line 1-9) plant, which was asexually propagated by cutting, was obtained from Nanchuan District (29°N and 107°E), Chongqing City, China. Seven independent organs from *G. jasminoides*, including the root, stem, leaf, flower, fruitlet, green fruit, and red fruit, were collected. The fruitlet, green fruit, and red fruit represented different maturity. In total, 21 samples including three biological replicates for each organ were gathered. All samples were divided into two portions, which were used for the measurement of crocin content and RNA sequencing. The pooled young leaves were used to DNA extraction for Illumina and ONT sequencing.

*ONT sequencing and assembly.* Following the methods for megabase-size DNA preparation, we extracted the high-molecular-weight (HMW) genomic DNA of *G. jasminoides*, which was used to construct paired-end, mate-pair and ONT libraries. The HMW gDNA was randomly fragmented using a Megaruptor, then the large fragments were selected and purified using BluePippin and AMPure beads. After end-prep, ligation of sequencing adapters, and tether attachment, the fragments were sequenced on the ONT GridION X5 platform with 6 nanopore flow cells (v9.4.1). Base calling was performed using the Oxford Nanopore base caller Guppy (v1.8.5). Canu (v1.7) was used to correct, trim and assemble the ONT raw reads with the default parameters. The correction-free approach, named Minimap/miniasm, was also independently performed with the recommended parameters. The assembler SMARTdenovo was also used for assembly with the corrected and trimmed ONT reads as input. The Canu-SMARTdenovo contigs were polished three times with Pilon (v1.22) using Illumina short reads. The final scaffolds were constructed with the polished contigs and corrected ONT reads using SSPACE-LongRead (version 1.1) and heterozygous sequences were removed using Purge Haplotigs. The quality of the genome assembly was estimated by searching for Benchmarking Universal Single-Copy Orthologs (BUSCO v4.0, embryophyte profile) with Embryophya *odb* 10 dataset. Illumina sequences from the *G. jasminoides* DNA and RNA libraries were mapped to evaluate the quality of the assembled genome using BWA (Burrows-Wheeler Aligner).

*Chromosome construction using Hi-C.* Fresh tissue of *G. jasminoides* was used to construct a Hi-C sequencing library. Steps included chromatin crosslinking, chromatin digestion with Hind III, biotin labeling and end repair, DNA purification, streptavidin pull-down of labeled Hi-C ligation products, and construction of an Illumina sequencing library. The clean sequences were mapped to the draft genome, and valid Hi-C reads were used to correct the draft assembly. Then, the draft genome of *G. jasminoides* was assembled into chromosomes (2n = 22) using Lachesis.

*Genome annotation and RNA-Seq analysis*. Annotation of structural repeats in the *G. jasminoides* genome was performed using the RepeatModeler (http://www.repeatmasker.org/RepeatModeler/; v1.0.9) package, which combines RECON and RepeatScout to identify and classify the repeat elements. The long terminal repeat retrotransposons (LTR-RTs) in *G. jasminoides* were identified using LTR_Finder (v1.0.6) and LTR_retriever with the default parameters. The repeat sequences were masked by RepeatMasker (v4.0.6) (http://www.repeatmasker.org/).

RNA-Seq on the HiSeq 4000 platform was performed for 21 samples. The short reads were assembled *de novo* using Trinity (v 2.2.0), and peptide sequences were predicted with TransDecoder (v2.1.0) (https://github.com/TransDecoder). The masked *G. jasminoides* genome annotation was *ab initio* predicted using the MAKER (v2.31.9) annotation pipeline, integrating the assembled transcripts of *G. jasminoides* and protein sequences from *G. jasminoides,* *C. canephora,* and *A. thaliana*. Noncoding RNAs were annotated by aligning to the Rfam database using INFERNAL (v1.1.2), and miRNAs were further analyzed by performing BLASTN searches against the miRNA database. The RNA-Seq reads from different *G. jasminoides* organs were aligned to the masked genome using HiSAT2 (v2.0.5), and the FPKM values of annotated genes in the reference genome were calculated using Cufflinks (v2.2.1).

The amino acid sequences of proteins from *G. jasminoides* and nine other angiosperms were clustered into orthologous groups using OrthoMCL (version 2.0.9). Phylogenetic analyses of single-copy orthologous genes were performed using the RAxML package (version 8.1.13) using the JTT+G+I substitution model for amino acid sequences with 1000 bootstrap replicates. Divergence times were directly estimated based on the divergence times of *P. trichocarpa* - *G. max* (94-127 MYA) and *B. distachyon* - *Z. mays* (40-53 MYA) obtained from TimeTree (http://www.timetree.org). The Markov Cluster Algorithm (MCL) was used to identify species-specific gene groups. CAFÉ (version 3.1) was used to predict gene family expansion and contraction. Genome synteny analyses were performed using the CoGe web suite, [www.genomevolution.org](http://www.genomevolution.org), according to methods described elsewhere.

*Genome sequencing and assembly*. The genome size of *G. jasminoides* was preliminarily estimated by flow cytometry (BD FACSCalibur, USA). The OTTO 1 nucleus extracting solution was used to extract nuclei, and propidium iodide was used as fluorochrome. The *G. jasminoides* genome size was predicted to be 550.6 ± 9 Mb (±SD) when *Populus trichocarpa* (480 Mb) and *Glycine max* (1100 Mb) were used as internal standards.

A genome survey using Illumina paired-end sequencing data was also performed. The genome size (547.5 Mb) and high heterozygosity (2.2%) were calculated using Kmerfreq and GCE based on a 17 *k*-mer distribution (Fig. S1).

Short-insert paired-end libraries (250 bp and 500 bp) and large-insert mate-pair libraries (2 kb, 5 kb, 10 kb and 20 kb) were prepared for 2 × 125 bp sequencing on the Illumina HiSeq 4000 platform (Table S1). Raw reads were filtered to remove adapters and low-quality reads prior to *de novo* assembly using Trimmomatic (v0.36). In total, 161,025,738,612 bp of sequence was produced and assembled using ALLPATHS-LG (v 52488) with the short reads of two fragment libraries and four jumping libraries as input. The assembly was 635.6 Mb (28% N bases) in size and composed of 58,859 scaffolds (N50, 60.6 kb) (Table S3).

In order to improve the heterozygous genome assembly, we performed Oxford Nanopore Technology (ONT) sequencing on the ONT GridION X5 platform with 6 flow cells and generated 32.4 Gb of sequence with an average 5.4 Gb per flow cell (Table S2). The average length and N50 of pass reads were 15.4 kb and 21.6 kb, respectively. The filtered data with Q score > 7 were corrected using the Canu correction packages, yielding 25.2 Gb corrected data with N50 of 22.4 kb. The corrected reads were assembled using SMARTdenovo with kmer of 17. We also generated assemblies using different software including Canu and minimap/miniasm, and the assembly results are shown in Table S3. Three polishing steps using 160 Gb of Illumina sequencing data were performed to improve the assembled genome, and a contiguous assembly 677.9 Mb in size with a contig N50 of 700.1 kb produced (Table S3). Because of the high heterozygosity, we used Purge Haplotigs to remove the heterozygous sequences, and the purged genome was assembled into chromosomes using Hi-C Pro and LACHESIS. The detailed correction, assembly and polishing parameters were as follows:

1. Canu correction

canu -correct -d asm -p gardenia -fast genomeSize=635m -nanopore-raw Gardenia_nanopore_raw.fastq

1. SMARTdenovo assembly

perl smartdenovo.pl -c 1 -t 36 -k 17 -p gardenia_smartdenovo asm.correctedReads.fasta > smartdenovo.make

make -f smartdenovo.make

1. 3 × Pilon

bwa index gardenia.smratdenovo.fasta

bwa mem -t 48 gardenia.smartdenovo.fasta 250_R1_clean.fastq 250_R2_clean.fastq >250_pilon.sam

samtools view -bhS -o 250_pilon.bam 250_pilon.sam

samtools sort 250_pilon.bam 250_sorted_pilon

samtools index 250_sorted_pilon.bam

…..

java -Xmx500G -jar pilon-1.22.jar --genome gardenia.smartdenovo.fasta --frags 250_sorted_pilon1.bam --frags … --jumps… --output gardenia.smratdenovo_polish_pilon1.fas --threads 48

This step was repeated three times.

1. Purge Haplotigs

bwa index gardenia.smratdenovo_polish_pilon3.fas

bwa mem -t 48 gardenia.smratdenovo_polish_pilon3.fas 250_R1_clean.fastq 250_R2_clean.fastq >250.sam

samtools view -bhS -o 250.bam 250.sam

samtools sort 250.bam 250_sorted.bam

purge_haplotigs readhist -b 250_sorted.bam.bam -g purge.fasta -t 32

purge_haplotigs contigcov -i 250_sorted.bam.bam.gencov -l 30 -m 80 -h 145 -o coverage_stats.csv -j 80 -s 80

purge_haplotigs purge -g purge.fasta -c coverage_stats.csv -o purge2 -t 24

1. Hi-C Pro + LACHESIS

Hi-C Pro was used to normalize the Hi-C raw data for the use of LACHESIS according to the instructions:

Hi-C Pro: https://github.com/nservant/HiC-Pro

LACHESIS: https://github.com/shendurelab/LACHESIS.
