# Supplementary material for: Tandem gene duplications drive divergent evolution of caffeine and crocin biosynthetic pathways in plants
Source: BMC Biol. 2020 Jun 18;18:63. doi: 10.1186/s12915-020-00795-3 (PMC7302004; doi:10.1186/s12915-020-00795-3)
Supplement: Supplementary file 2 — Additional file 2: Figures S1-S26; Tables S1-S18. [file 12915_2020_795_MOESM2_ESM.docx]

**Additional file 2: Figures S1-S26; Tables S1-S18**


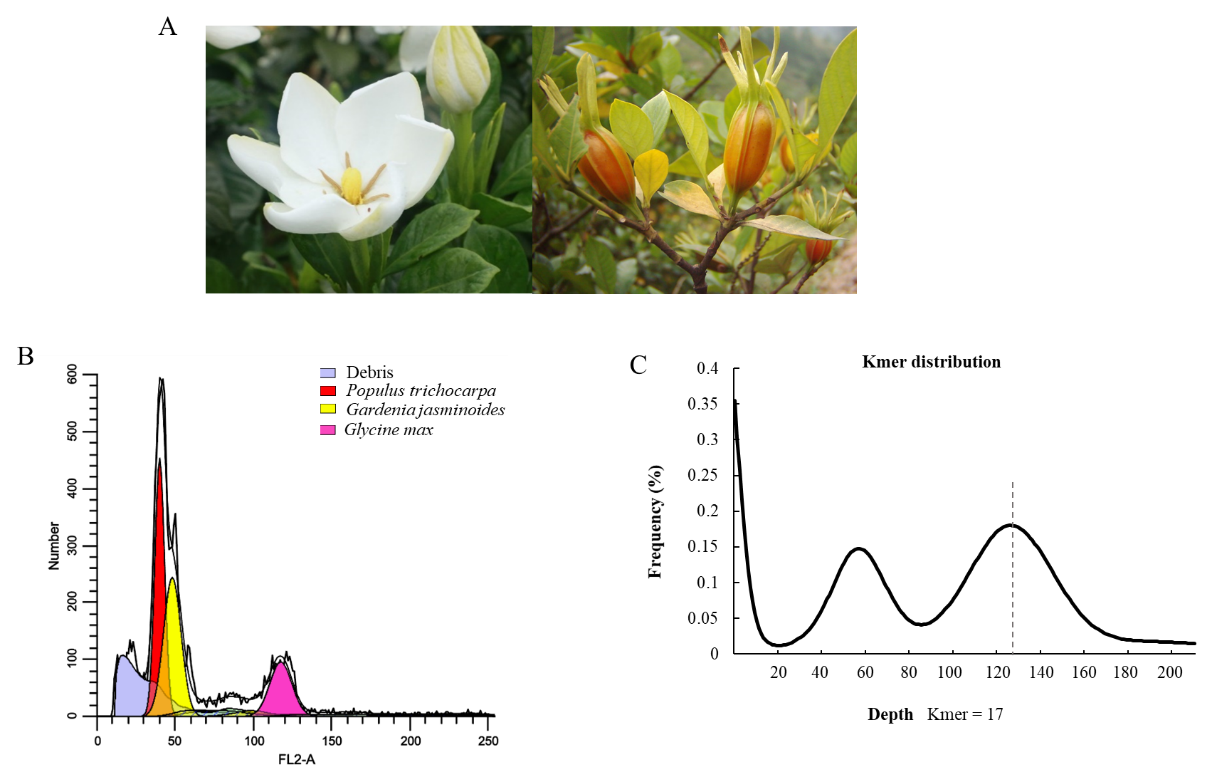


Additional file 2: Figure S1. G. *jasminoides* genome size estimation by flow cytometry and 17 *k*-mer distribution. A. *G. jasminoides* blooming flowers and mature fruits. B. Flow cytometry analysis using *Populus trichocarpa* (480 Mb) and *Glycine max* (1100 Mb) as internal standards. C. The 17 k-mer distribution of Illumina short reads reveals a high heterozygosity (2.2 %).


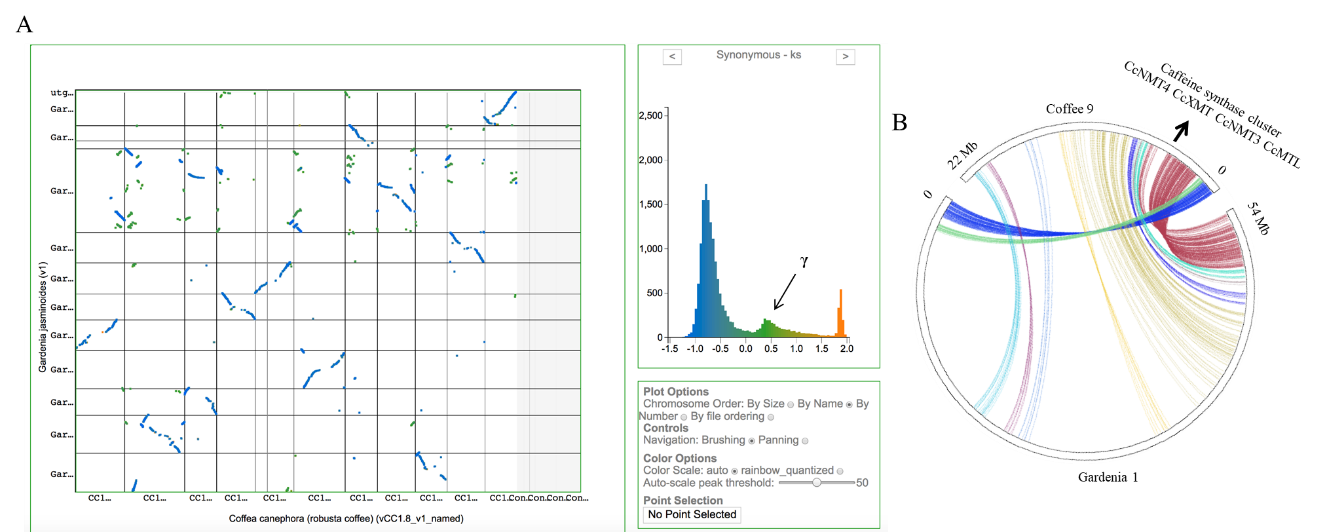


Additional file 2: Figure S2. comparative analysis of *Gardenia* and *Coffee* genome. A. The *Gardenia* genome shows considerable synteny with *C. canephora* (blue peak), as well as strong evidence for a gamma triplication peak around log_10_ *Ks* = 0.5 (green peak). B. Synteny of *Coffee* chr 9 and *Gardenia* chr1. The arrow represents the coffee gene cluster involved in caffeine biosynthesis.


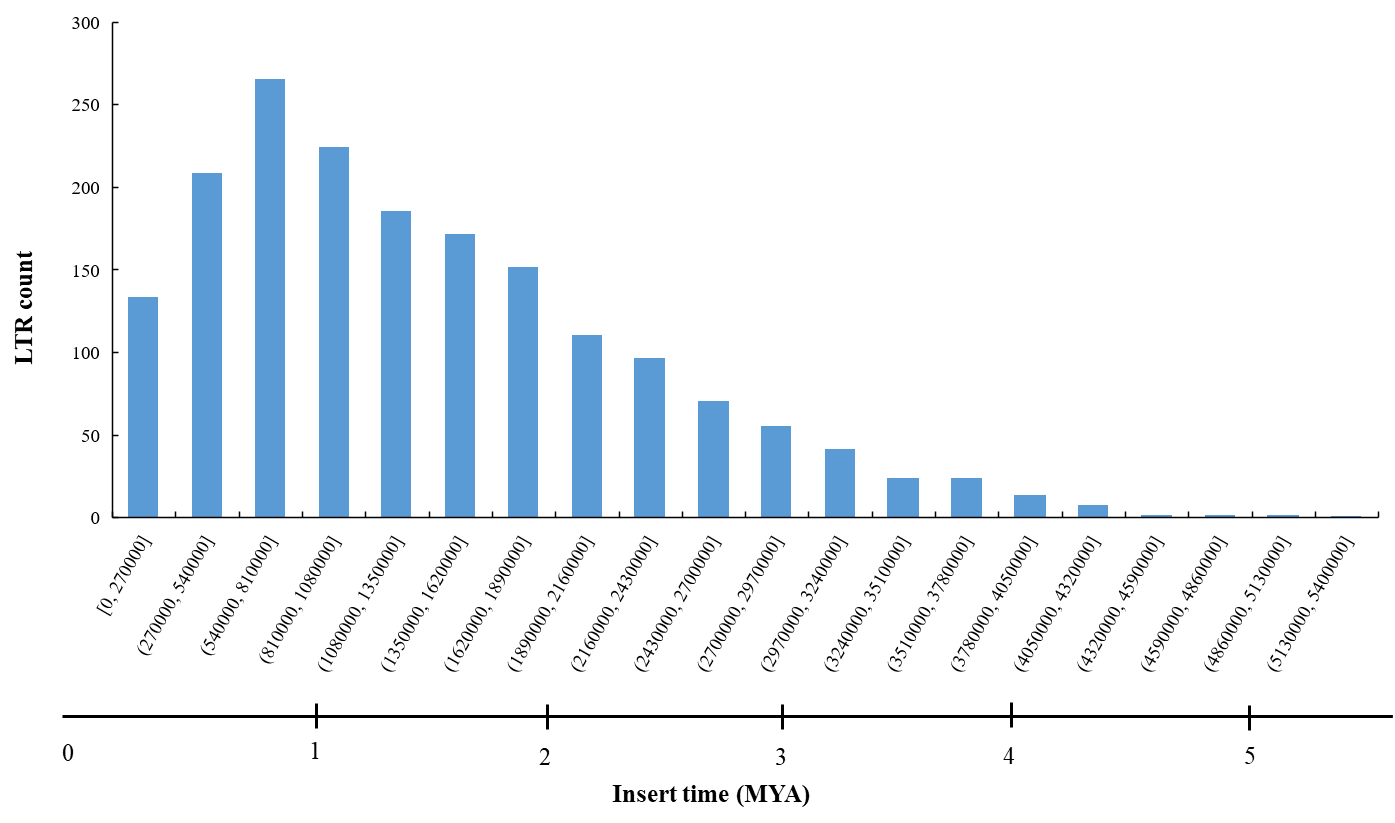


Additional file 2: Figure S3. Insertion time distribution of intact LTR-RTs in *G. jasminoides* assuming a mutation rate of *μ*=1.3×10^-8^ (per bp per year). Sequence divergence was corrected using the Jukes-Cantor model, which was applied in LTR_retriever (Ou and Jiang, 2018).


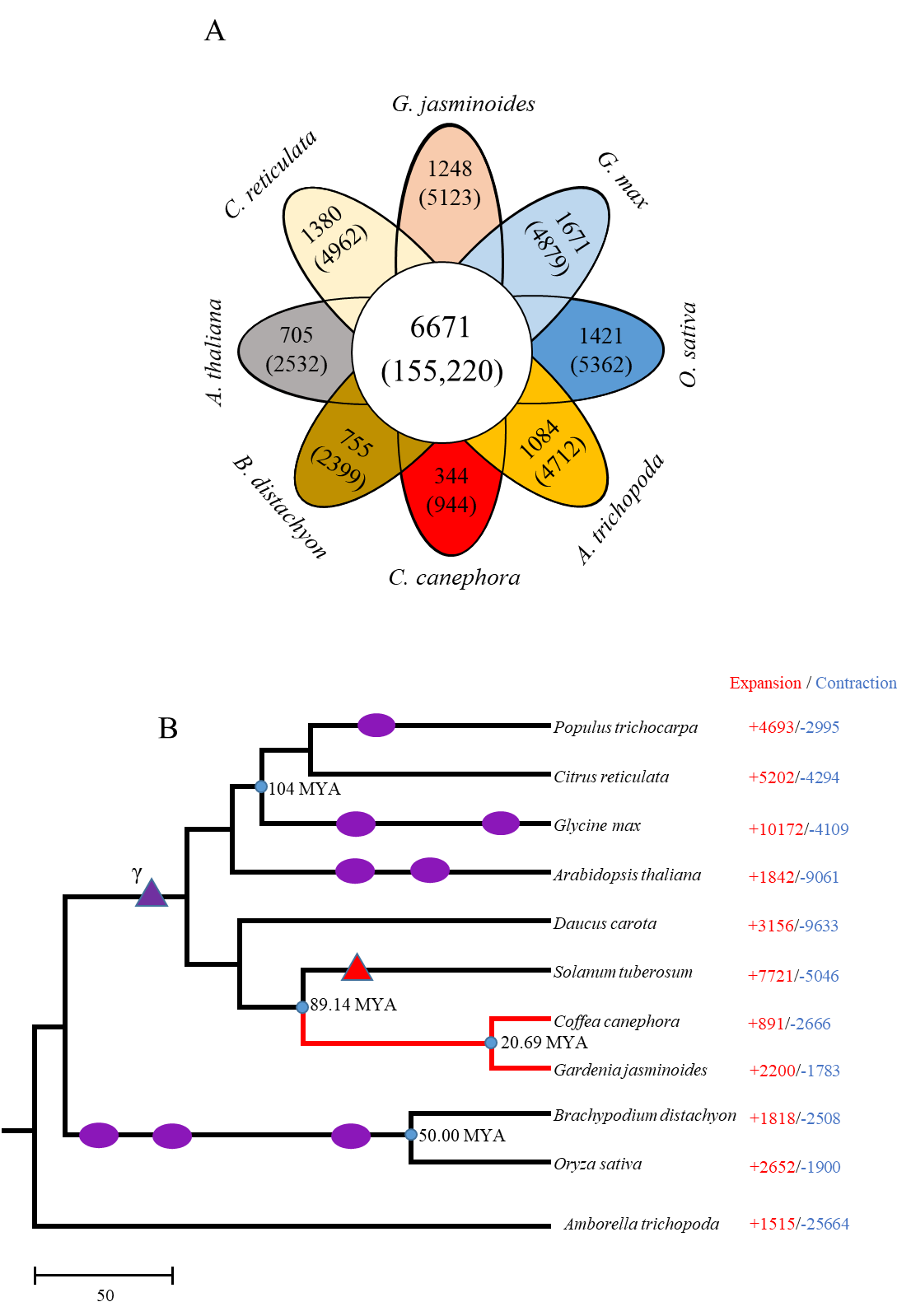


Additional file 2: Figure S4. The orthologous genes and phylogenetic analysis among *Gardenia* and other 10 angiosperms. A. Venn diagram illustrating the number of gene families and (in parenthesis) unique genes for *G. jasminoides* and those in common with other seven plant genomes. B. Expansion and contraction of gene families among 11 angiosperms. The phylogenetic tree was constructed using 121 single-copy orthologous genes. Colored numbers show the numbers of expanded (red) and contracted (blue) families in each plant species. Divergence times were estimated based on the divergence times of *P. trichocarpa*-*G. max* (94-127 MYA) and *B. distachyon*-*O. sativa* (40-53 MYA). Purple ellipses represent the known whole genome duplication events. The triangles represent the gamma (γ) and Solanaceae triplication events.


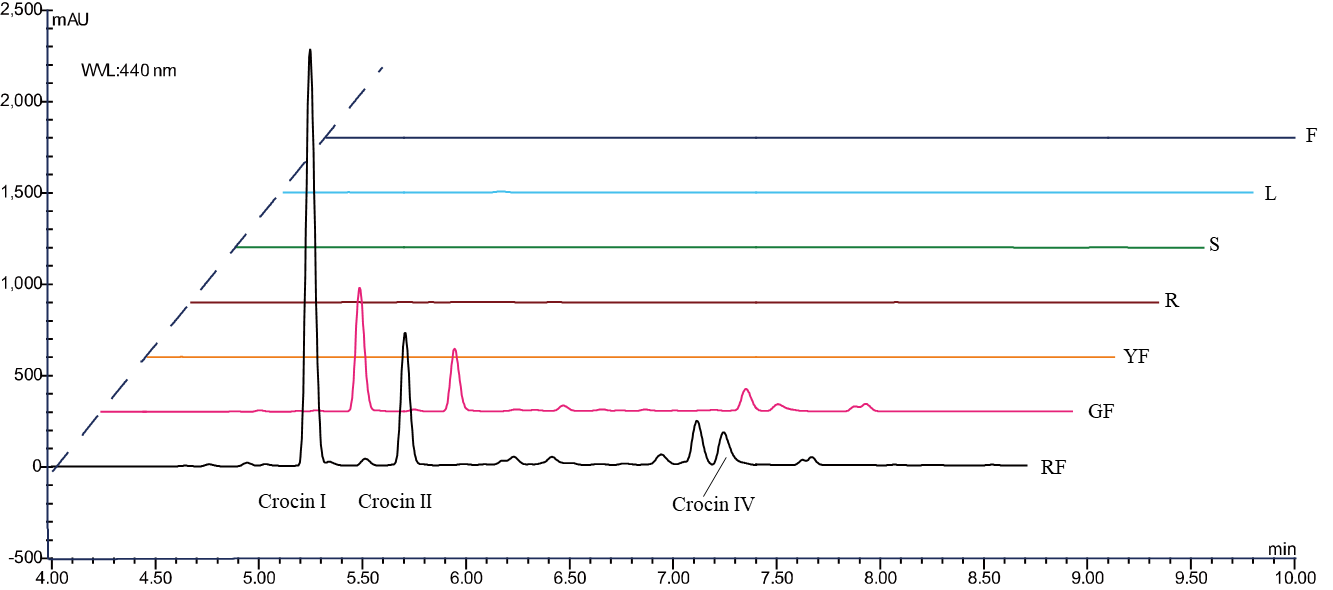


Additional file 2: Figure S5. Crocin content in *G. jasminoides* different organs (UPLC-DAD chromatograms, absorbance at 440 nm). F=flower, L=leaf, S=stem, R=root, YF=fruitlet, GF=green fruit, RF=red fruit. Crocin I, crocin II and crocin IV were detected in GF and RF.


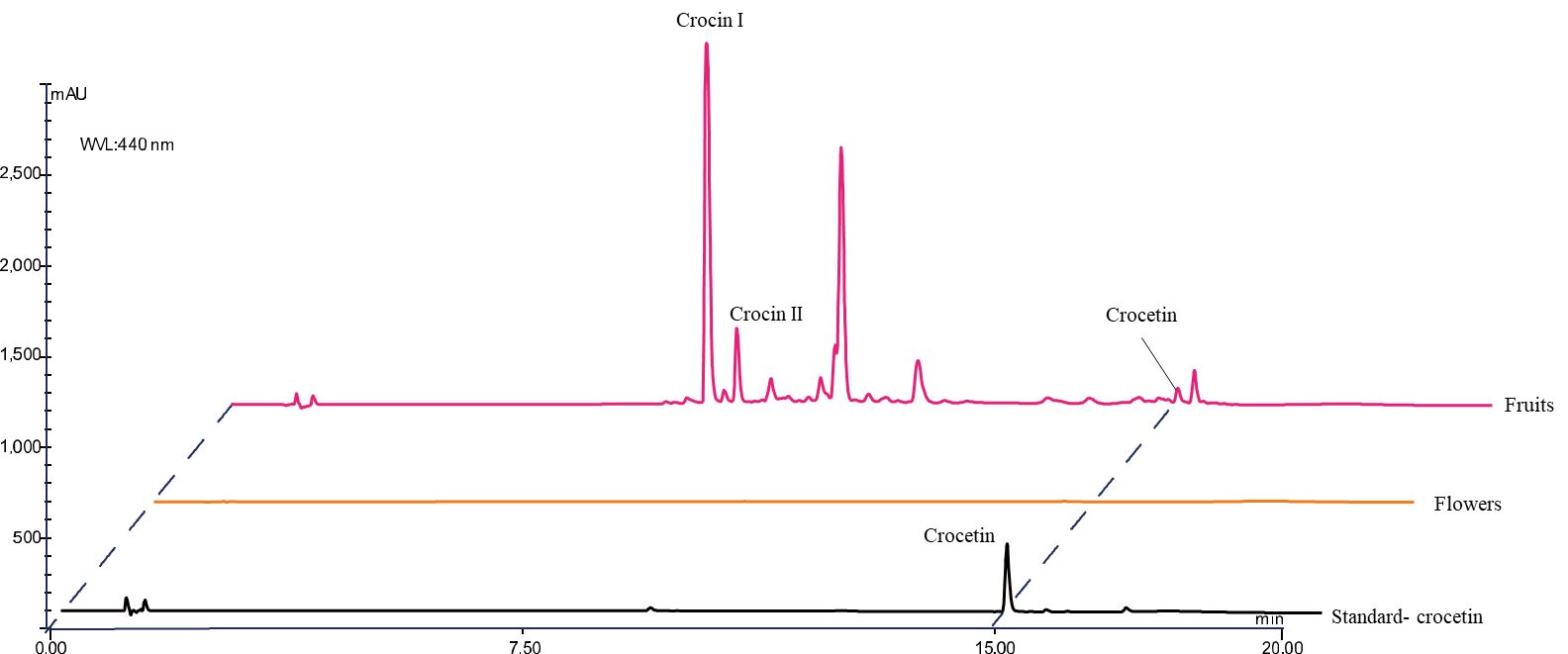


Additional file 2: Figure S6. Crocetin content in flowers and fruits of *G. jasminoides* (UPLC-DAD chromatograms, absorbance at 440 nm).


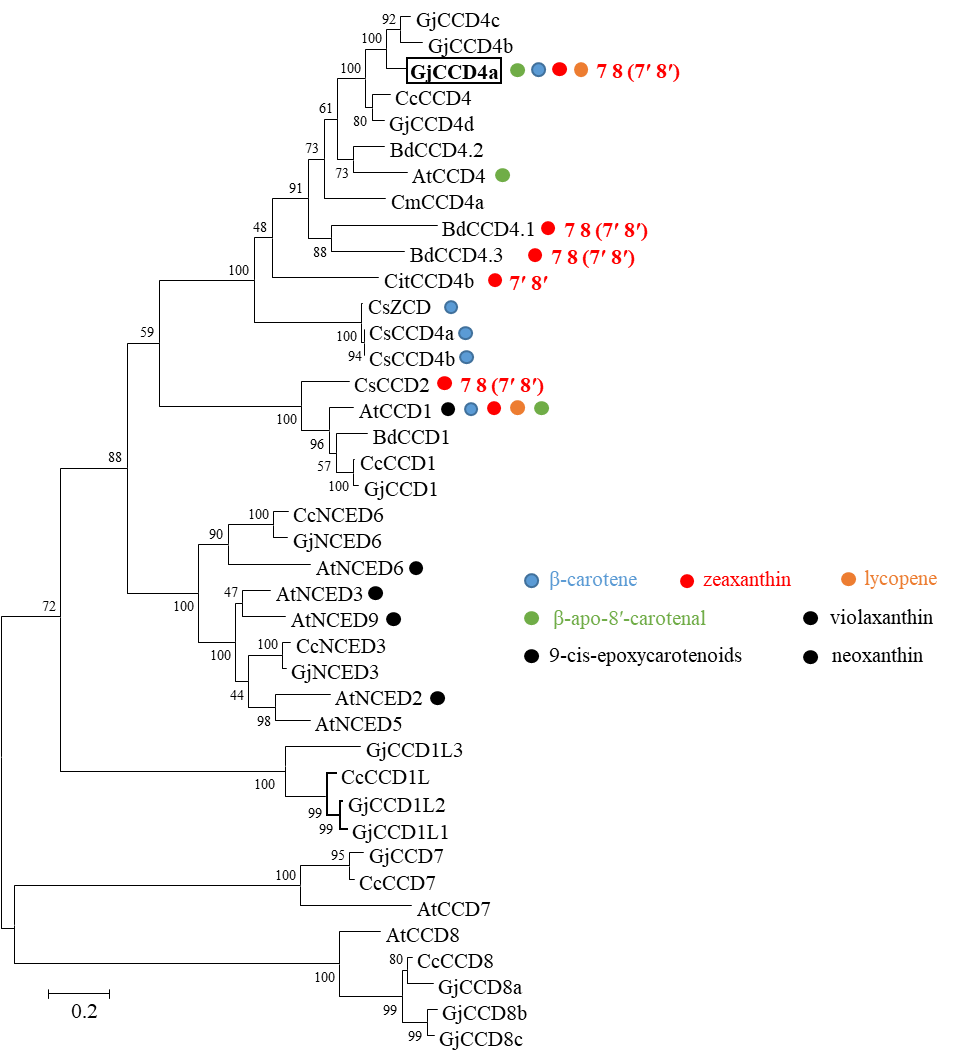


Additional file 2: Figure S7. Phylogenetic analysis of CCD family members from different species including *G. jasminoides*, *C. canephora*, *C. morifolium*, *A. thaliana*, *B. davidii* and *C. sativus* using Maximum Likelihood method. Dots of different colors represent different CCD substrates. The NCBI accession number are the following: AtCCD1 (NP_191911.1), AtCCD4 (NP_193652.1), AtCCD7 (NP_182026.5), AtCCD8 (NP_195007.2), AtNCED2 (NP_193569.1), AtNCED3 (NP_188062.1), AtNCED5 (NP_174302.1), AtNCED6 (NP_189064.1), AtNCED9 (NP_177960.1), CitCCD4b (ABC26012.1), CmCCD4a (ABY60885.1), CsCCD2 (AIG94929.1), CsCCD4a (ACD62476.1), CsCCD4b (ACD62477.1), CsZCD (Q84K96.1).


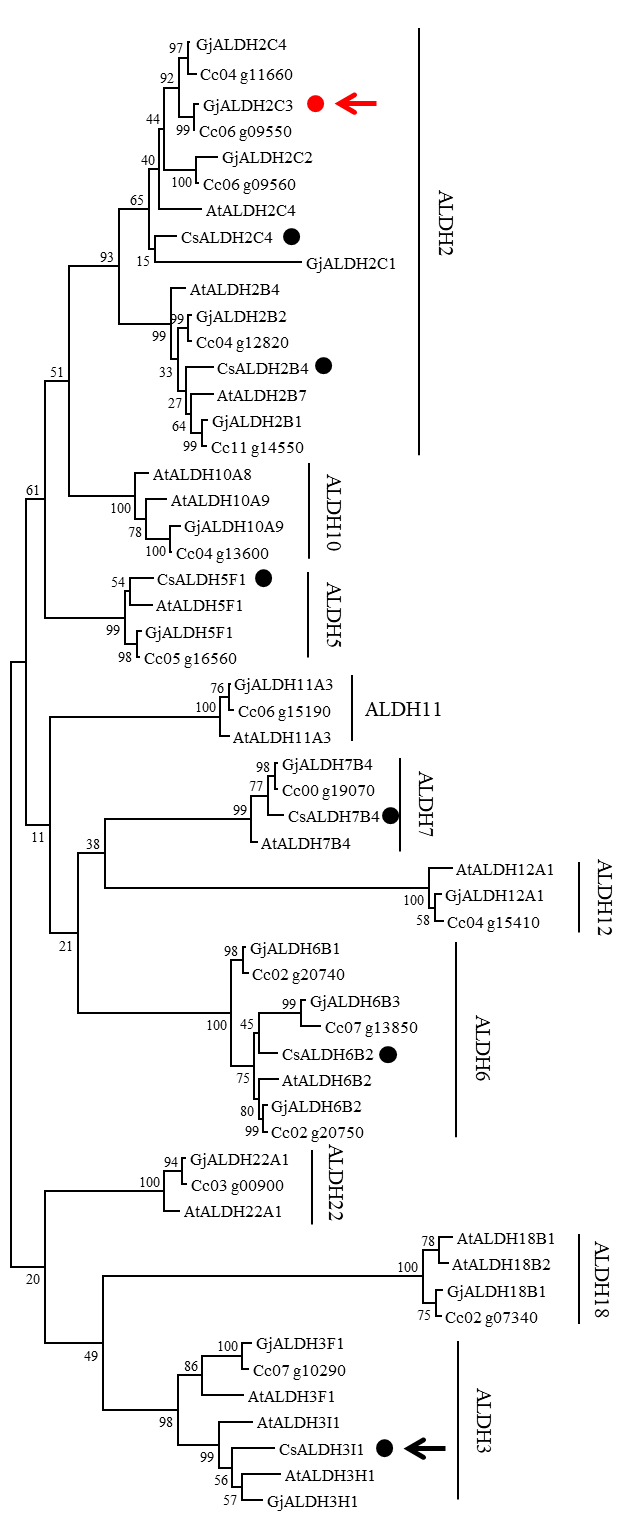


Additional file 2: Figure S8. Genome-wide identification and phylogenetic analysis of GjALDH family members in *G. jasminoides*, using Maximum Likelihood method. The black and red arrows indicate the ALDHs that catalyze crocetin dialdehyde conversion into crocetin in *C. sativus* and *G. jasminoides*, respectively.


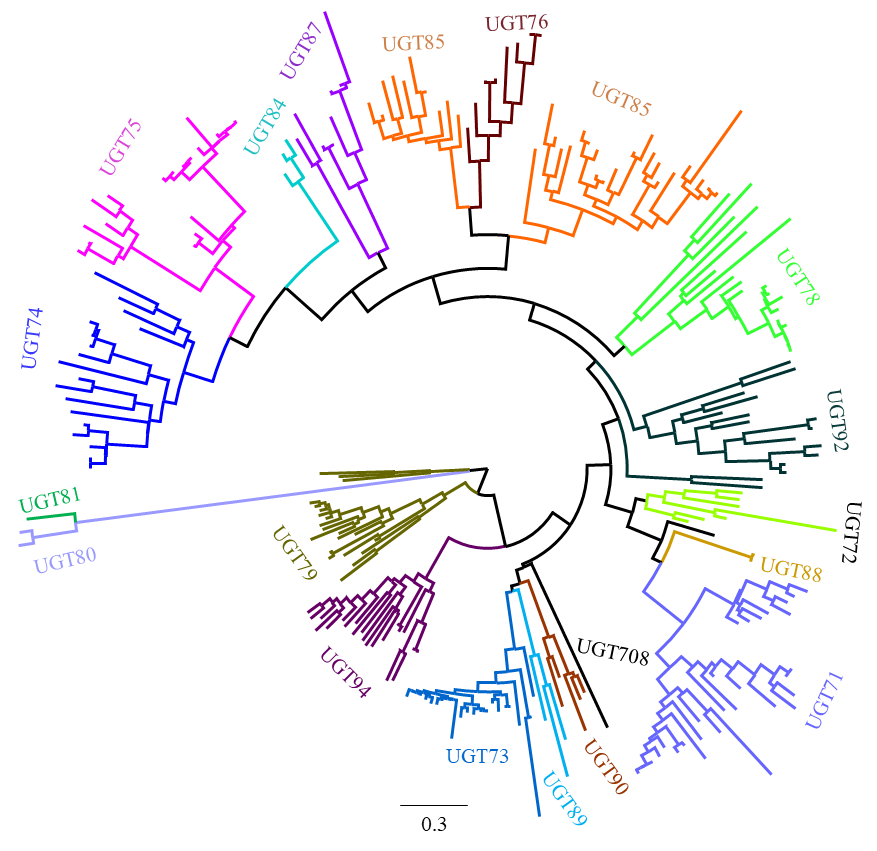


Additional file 2: Figure S9. Genome-wide identification and phylogenetic analysis of GjUGT family members in *G. jasminoides*, *C. canephora*, *A. thaliana* and *C. sativus*, using Maximum Likelihood method.


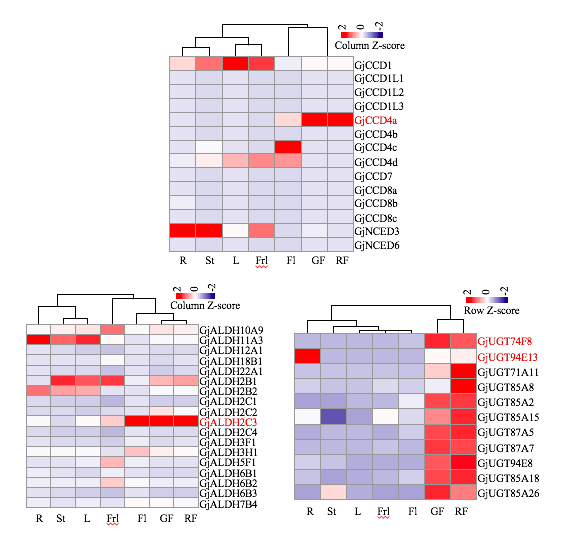


Additional file 2: Figure S10. Heatmap (Z-score) showing differential expression of different *CCD, ALDH, UGT*  genes in *G. jasminoides* organs and tissues. Genes involved in crocin biosynthesis are marked in red.


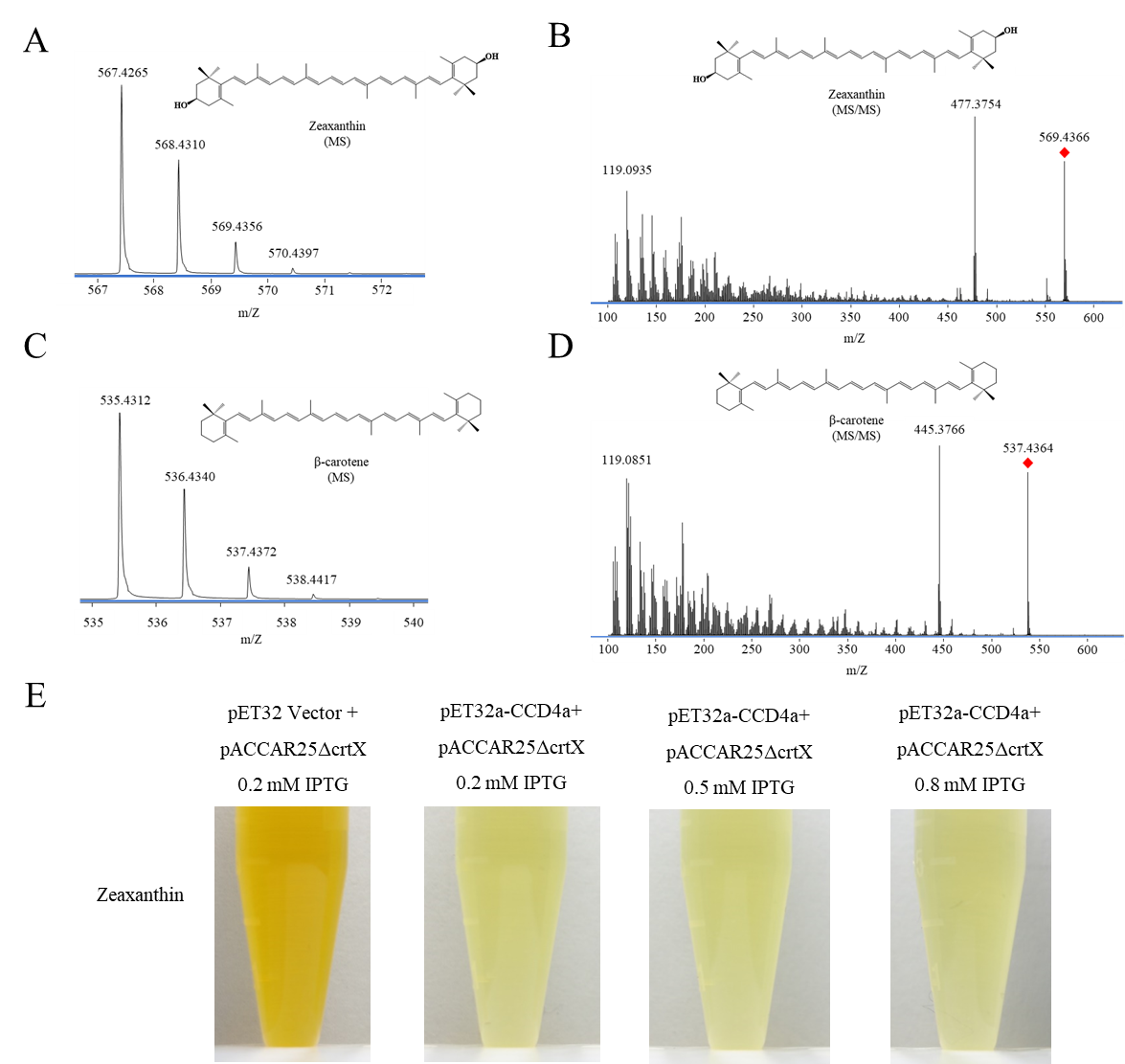


Additional file 2: Figure S11. MS and MS/MS spectra of zeaxanthin and β-carotene (A-D) and discoloration of *E. coli* zeaxantin-accumulating strain in the presence of the GjCCD4a enzyme (E).


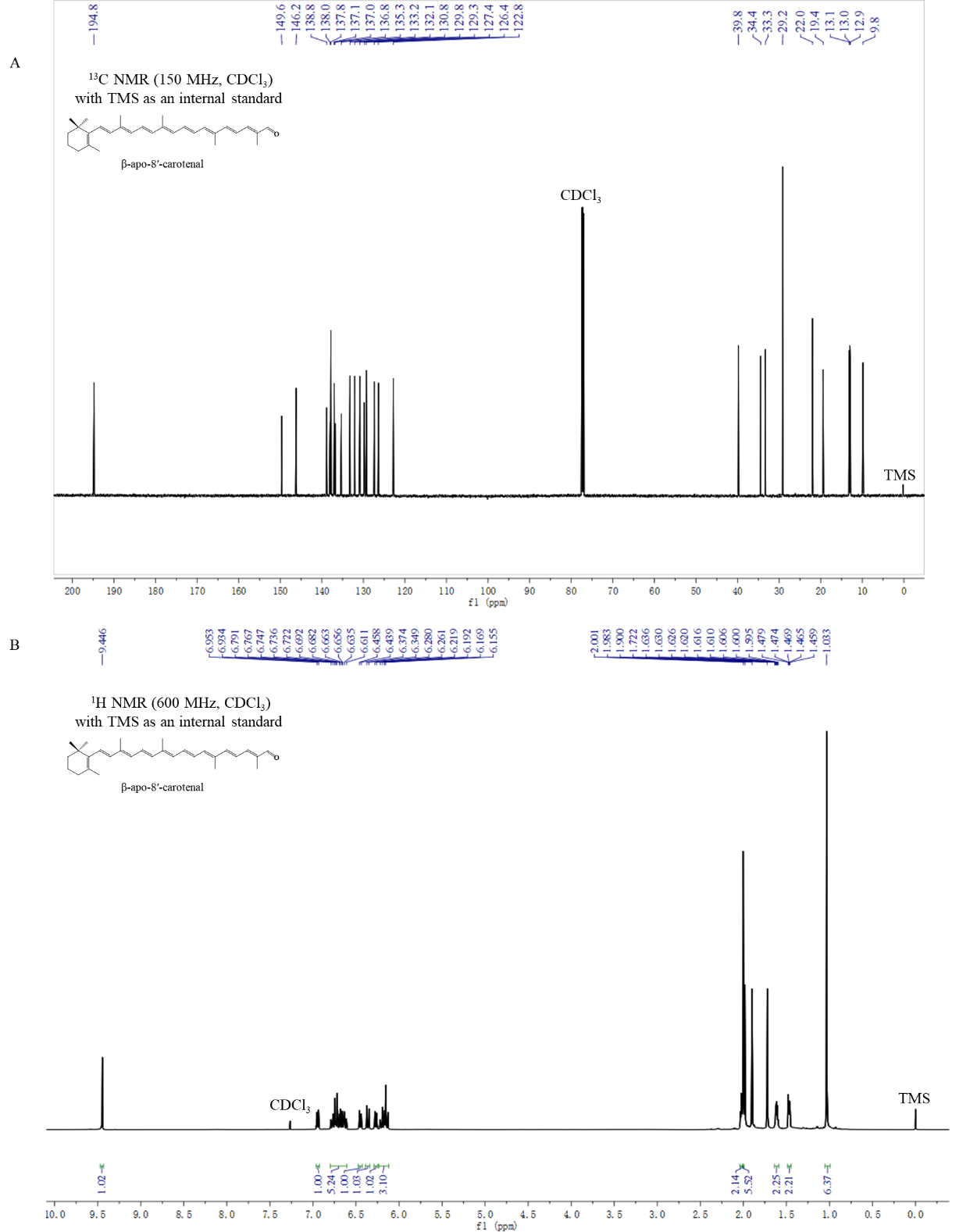


Additional file 2: Figure S12. NMR spectra of the intermediate product β-apo-8′-carotenal generated by the cleavage activity of GjCCD4a on β-carotene in bacteria.


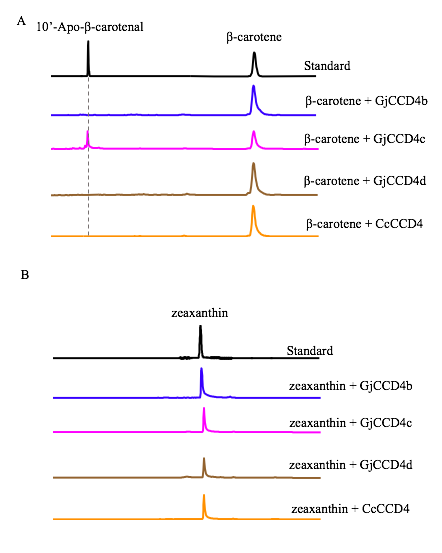


Additional file 2: Figure S13. Lack of 7/8, 7’/8’ cleavage activity by GjCCD4b-d and CcCCD4.


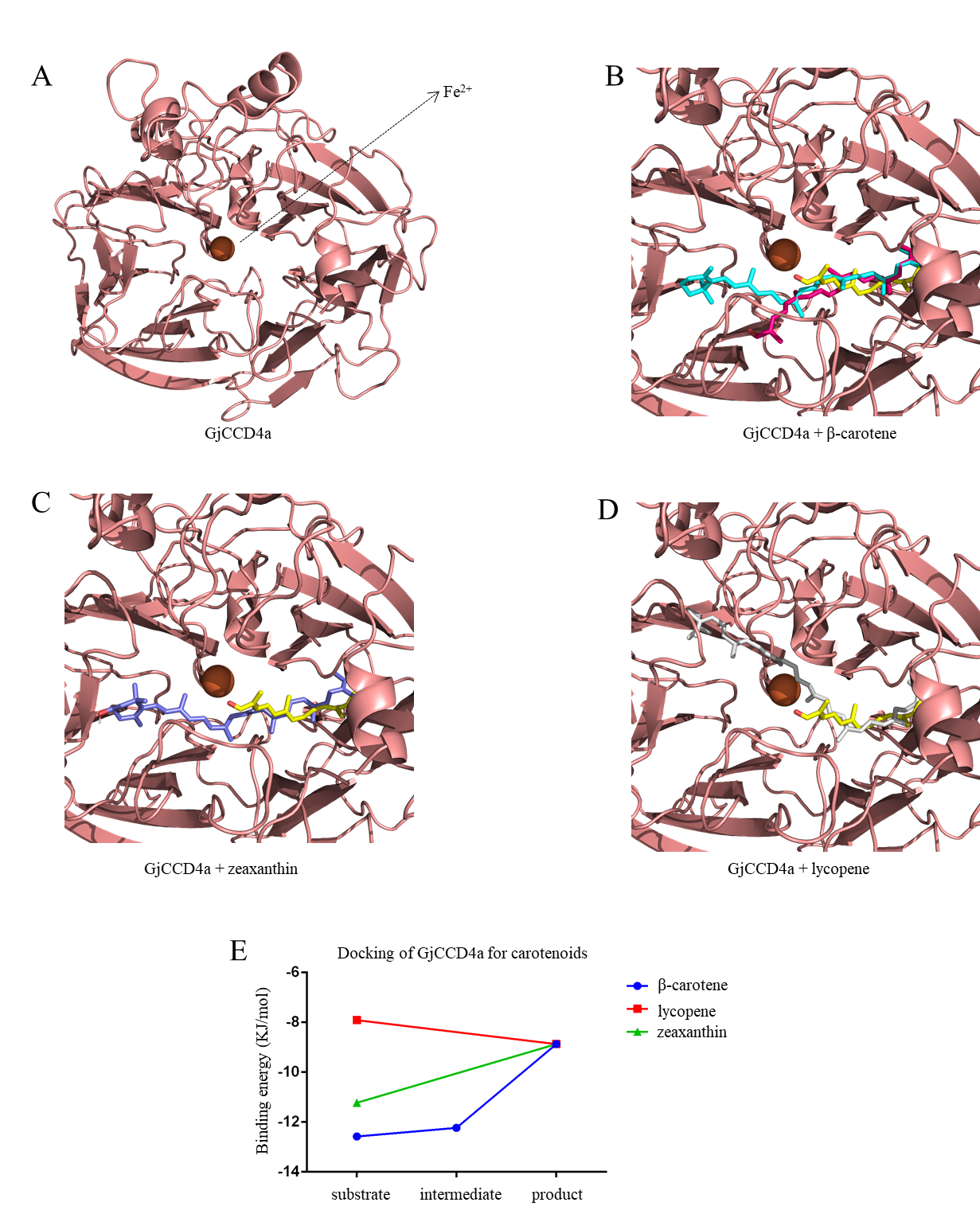


Additional file 2: Figure S14. Protein modeling and docking analysis of GjCCD4a towards three carotenoids. A. Predicted protein structure using 2biw (pdb) as model. The ferrous catalytic iron is indicated. B. Docking analysis showing the interactions with the substrate β-carotene (light blue) and its products β-apo-8′-carotenal (dark pink) and crocetin dialdehyde (yellow). C. Docking analysis showing the interactions with the substrate zeaxanthin (violet) and its product crocetin dialdehyde (yellow). D. Docking analysis showing the interaction with the substrate lycopene (grey) and its product crocetin dialdehyde (yellow). E. Binding energy of GjCCD4a docking to three carotenoids.

**
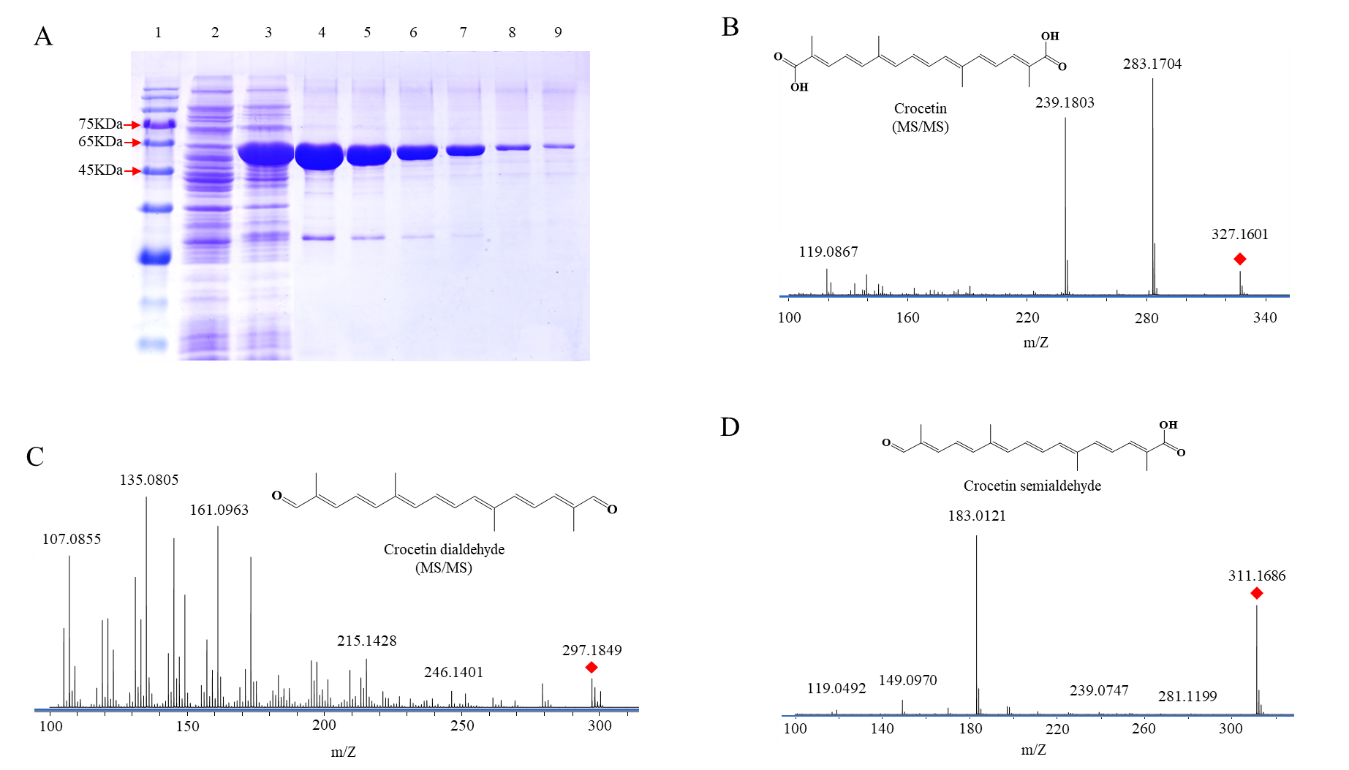
**

Additional file 2: Figure S15. Functional characterization of GjALDH2C3. A. SDS-PAGE analysis of purified GjALDH2C3. Lane 1: Marker; Lane 2: Empty pCold I vector; Lane 3: pCold-GjALDH2C3; Lanes 4-9 represent different concentration of imidazole for protein elution, including 300 mM, 400 mM, 500 mM, 500 mM, 500 mM, 500 mM, respectively. B. MS/MS analysis of crocetin. C. MS/MS analysis of crocetin dialdehyde. D. MS/MS analysis of crocetin semialdehyde.

**
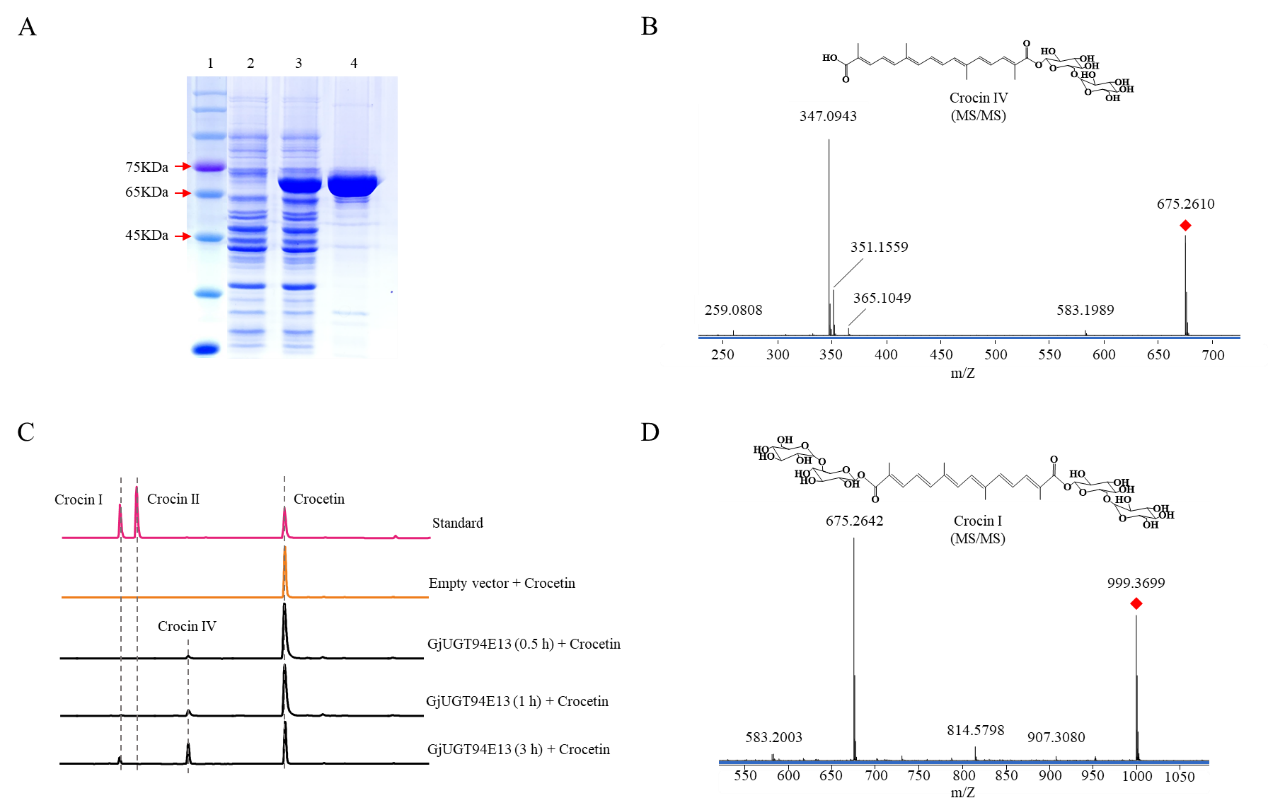
**

Additional file 2: Figure S16. Functional characterization of GjUGT94E13 activity towards crocetin. A. SDS-PAGE analysis of purified GjUGT94E13. Lane 1: Marker; Lane 2: Empty pET32a vector; Lane 3: pET32a- GjUGT94E13; Lanes 4: Purified GjUGT94E13 protein. B. MS/MS analysis of crocin IV. C. UPLC analysis of the products of GjUGT94E13 activity on crocetin. D. MS/MS analysis of crocin I.


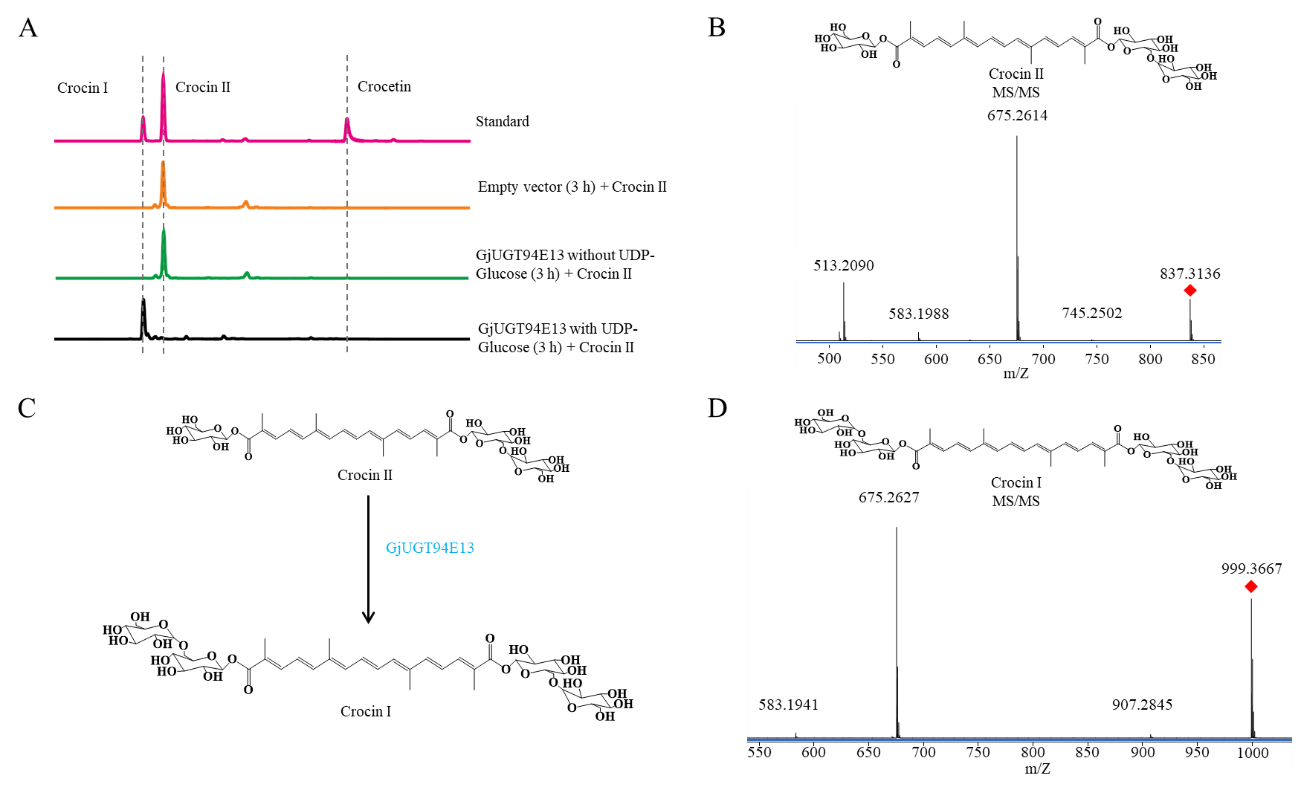


Additional file 2: Figure S17. Functional characterization of GjUGT94E13 activity towards crocin II. A. UPLC analysis of the products of GjUGT94E13 activity on crocin II. B. MS/MS analysis of crocin II. C. Proposed pathway from crocin II to crocin I. D. MS/MS analysis of crocin I.


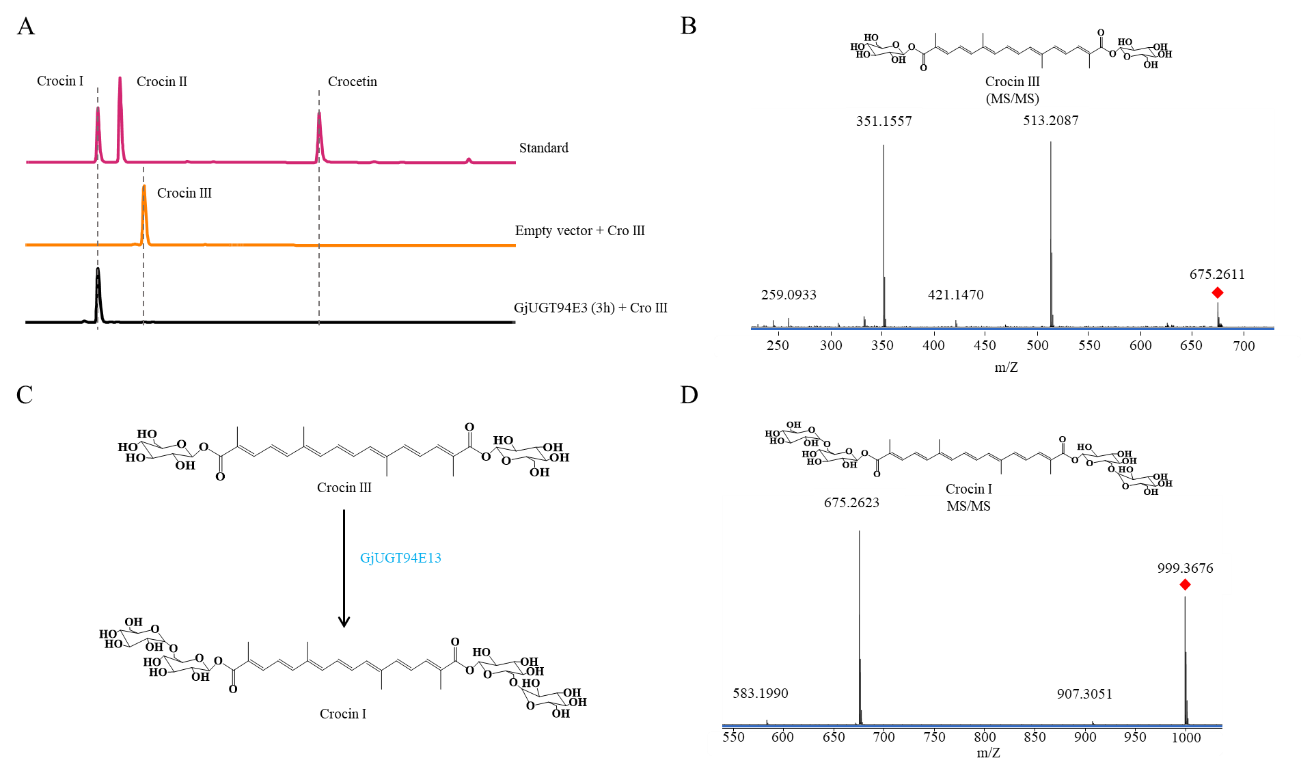


Additional file 2: Figure S18. Functional characterization of GjUGT94E13 activity towards crocin III. A. UPLC analysis of the products of GjUGT94E13 activity on crocin III. B. MS/MS analysis of crocin III. C. Proposed pathway from crocin III to crocin I. D, MS/MS analysis of crocin I.


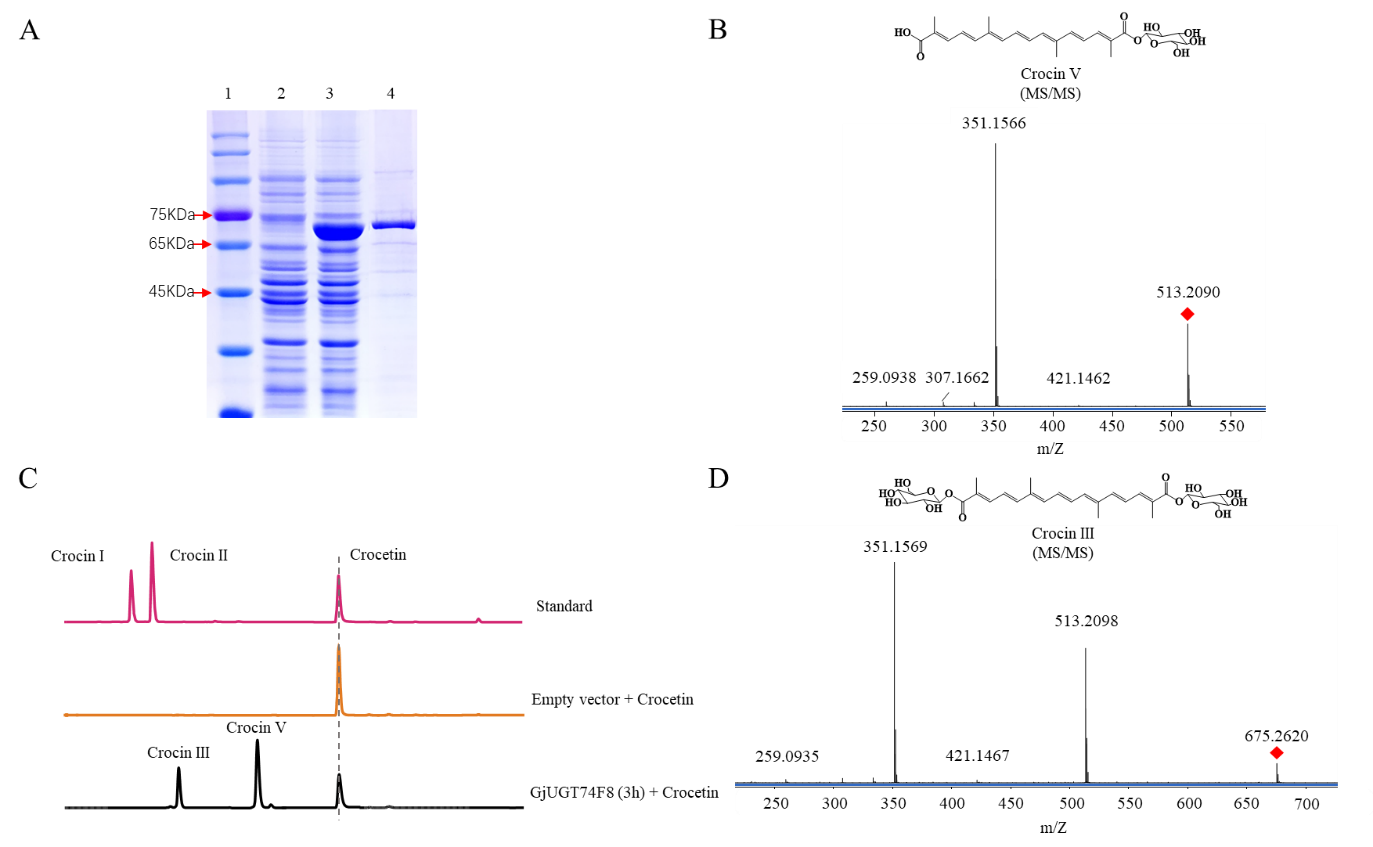


Additional file 2: Figure S19. Functional characterization of GjUGT74F8 activity towards crocetin. A. SDS-PAGE analysis of purified GjUGT74F8. Lane 1: Marker; Lane 2: Empty pET32a vector; Lane 3: pET32a- GjUGT74F8; Lanes 4: Purified GjUGT74F8 protein. B. MS/MS analysis of crocin V. C. UPLC analysis of the products of GjUGT74F8 activity on crocetin. D. MS/MS analysis of crocin III.


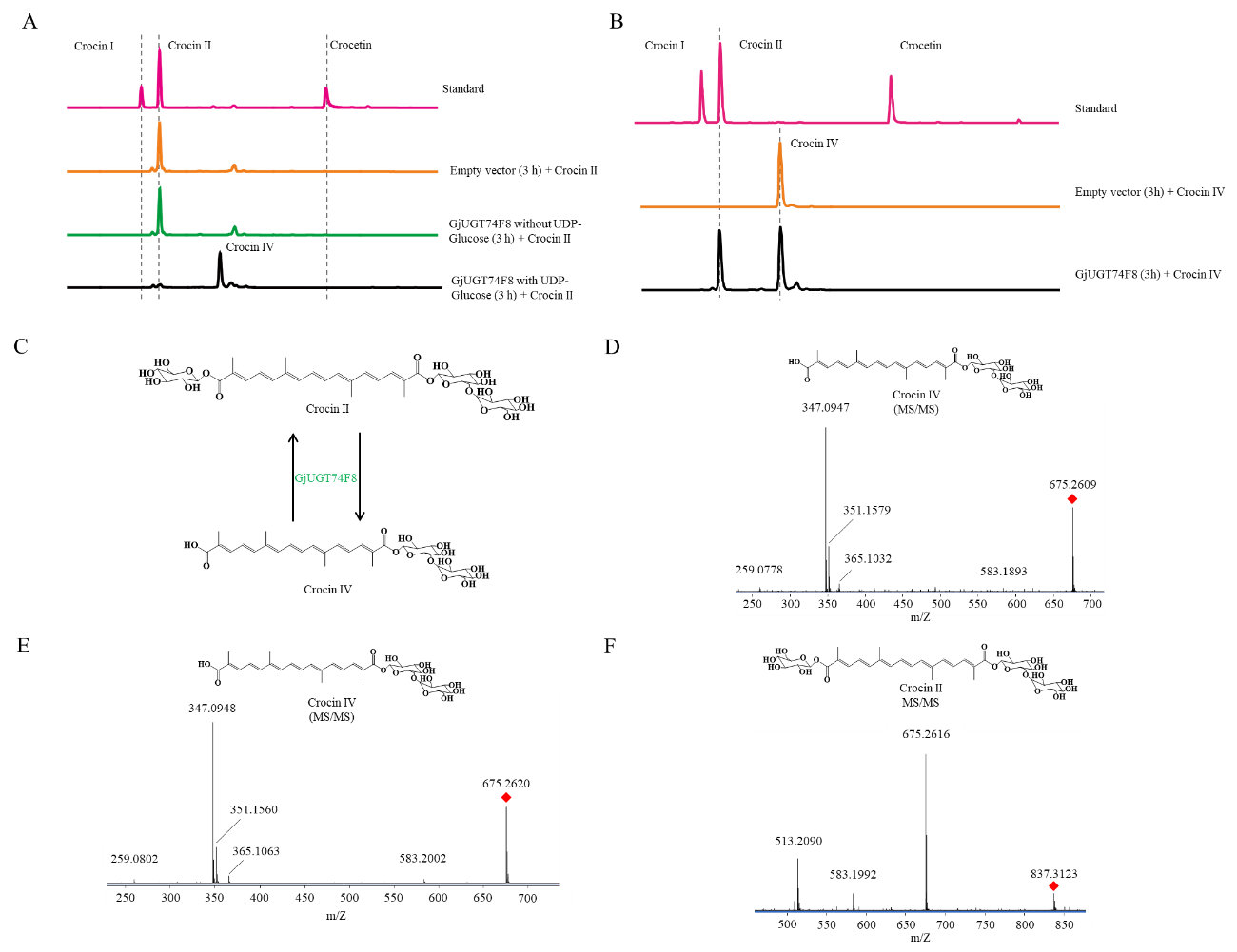


Additional file 2: Figure S20. Functional characterization of GjUGT74F8 activity towards crocin II and IV. A. UPLC analysis of the products of GjUGT74F8 catalysis with crocin II as the substrate. B. UPLC analysis of the products of GjUGT74F8 activity on crocin IV. C. Proposed pathway between crocin II and crocin IV. D. MS/MS analysis of substrate crocin IV. E. MS/MS analysis of product crocin IV. F. MS/MS analysis of product crocin IV.


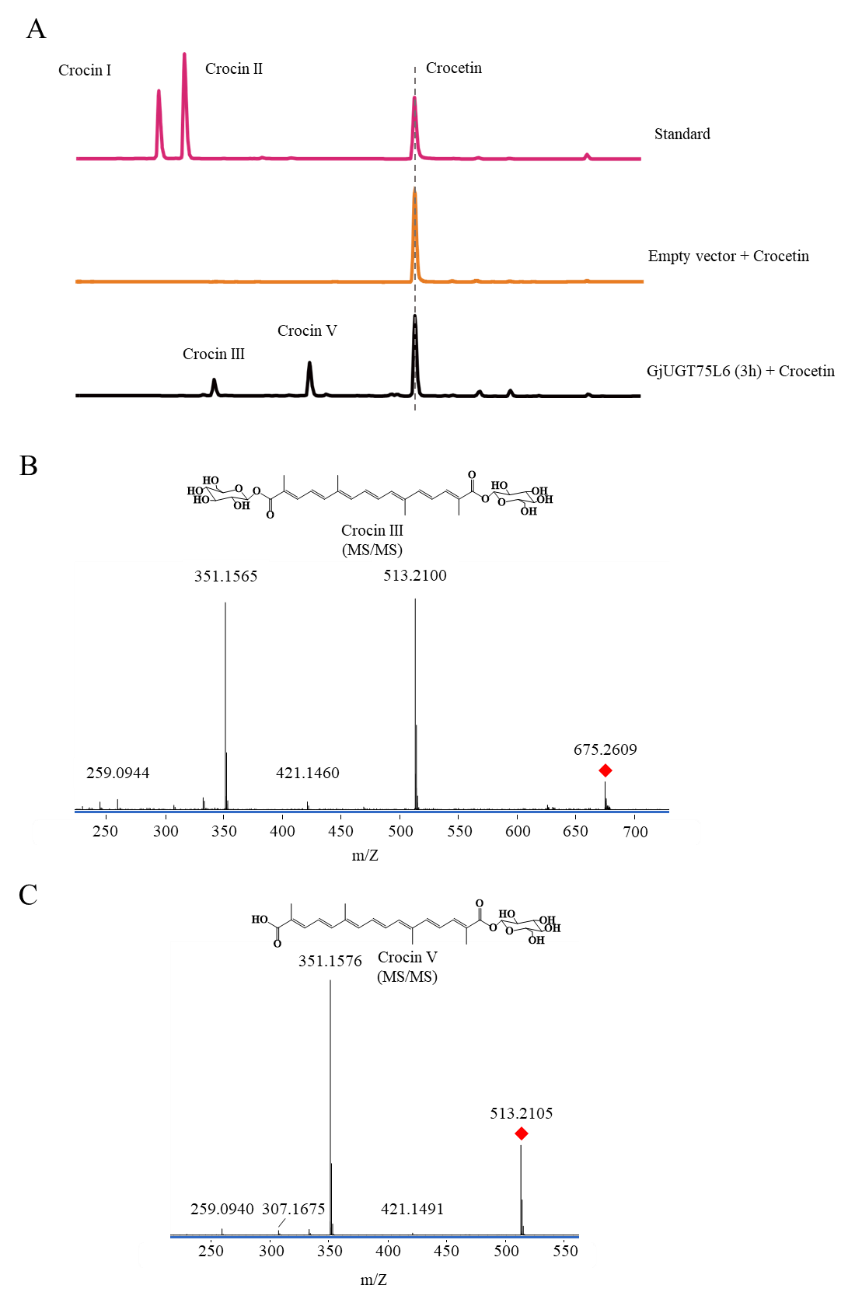


Additional file 2: Figure S21. Functional characterization of GjUGT75L6 activity towards crocetin. A. UPLC analysis of the products of GjUGT75L6 activity on crocetin. B. MS/MS analysis of crocin III. C, MS/MS analysis of crocin V.


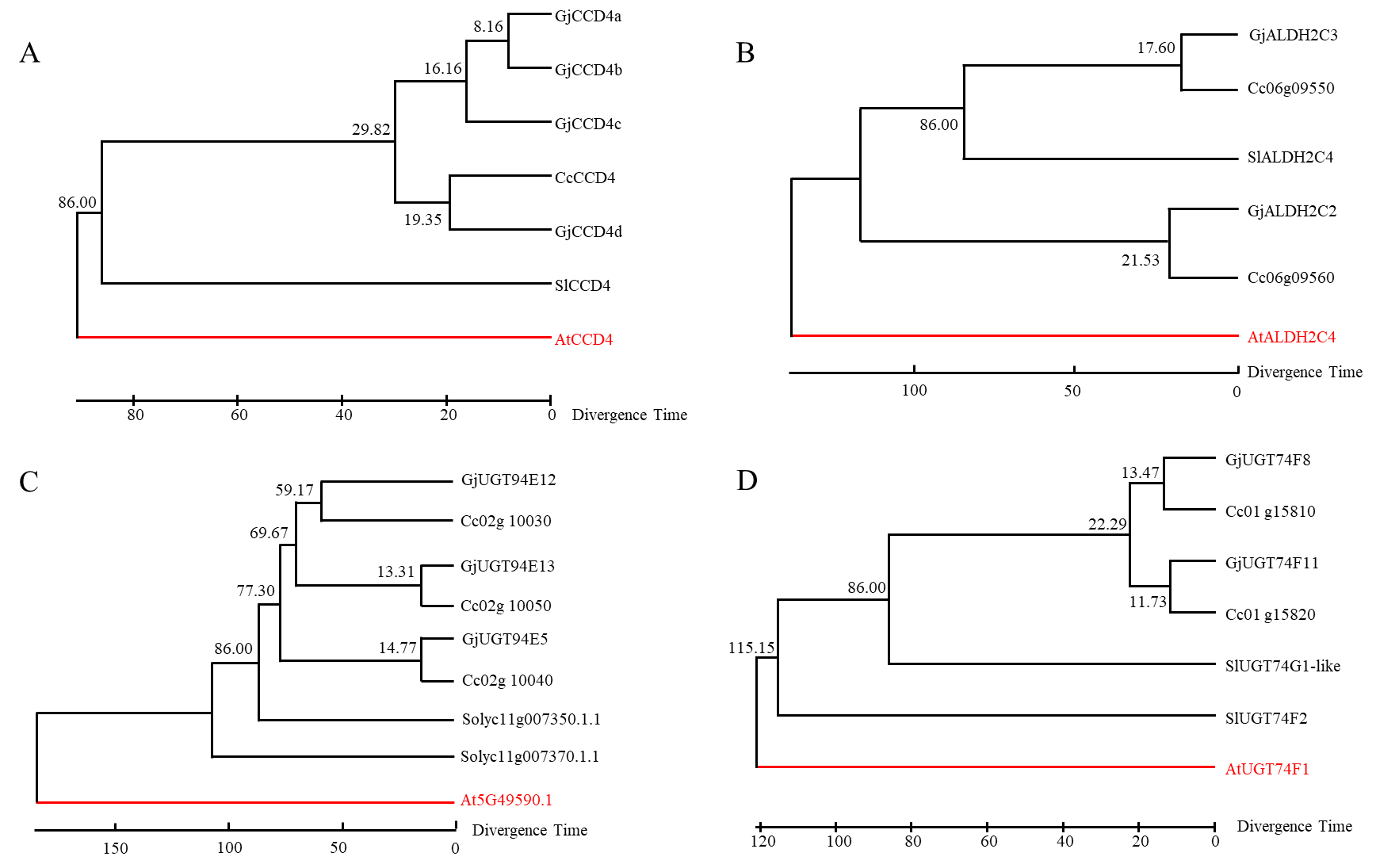


Additional file 2: Figure S22. Timetrees of CCD4, ALDH2C, UGT94E, UGT74F genes among *Gardenia*, *Coffea*, and Tomato. *A. thaliana* orthologs were chosen as outgroups. The timetrees were generated using the RelTime method on threes obtained with the Maximum Likelihood method. Divergence times for all branching points in the topology were calculated using the Maximum Likelihood method based on the Tamura-Nei model. The time calibration was performed assuming the divergence of Solanaceae from Rubiaceae 83-89 MYA.


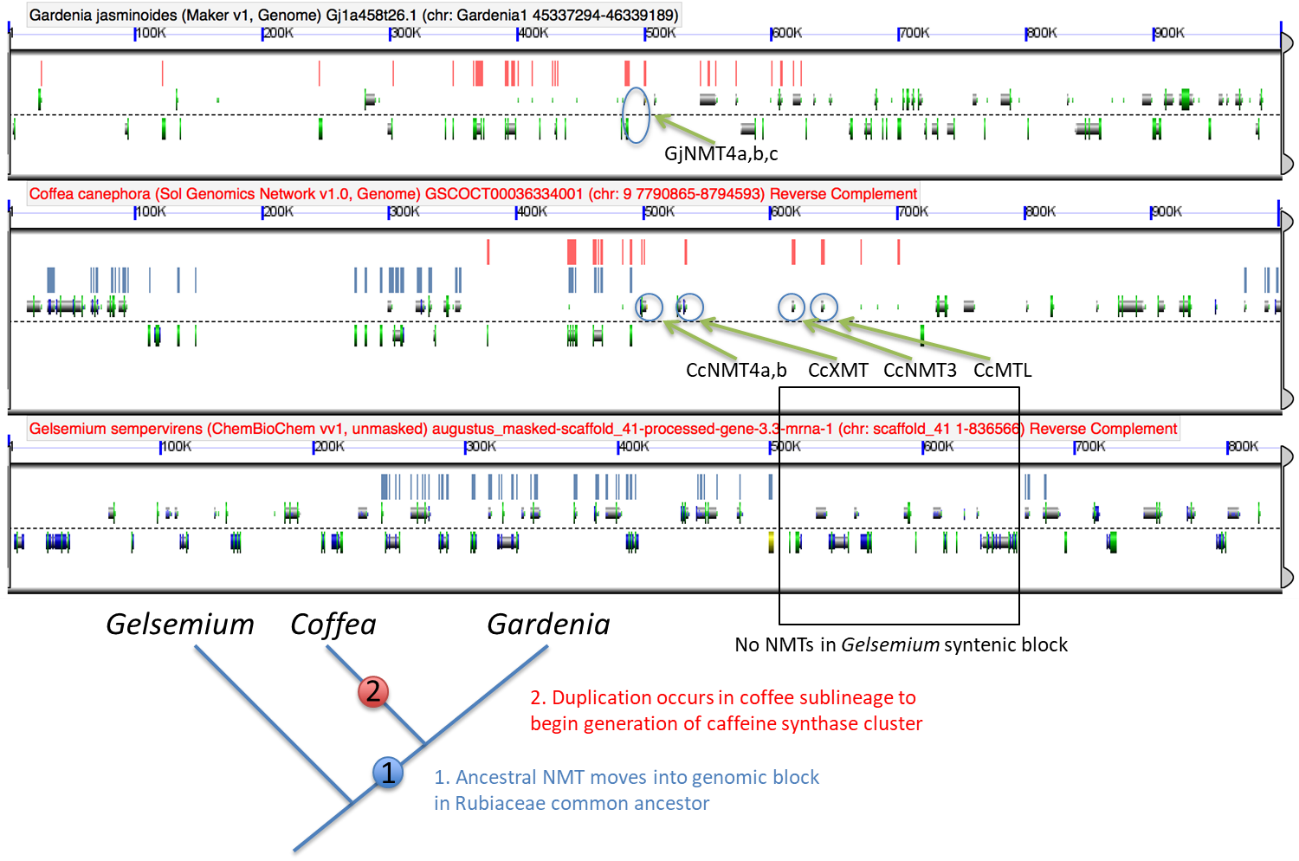


Additional file 2: Figure S23. The caffeine biosynthesis cluster evolved until after the Gelsemiaceae-Rubiaceae divergence. Synteny of the NMT cluster between *C. canephora* and *G. jasminoides* genomes showing that the beginnings of the caffeine synthase cluster already existed in Rubiaceae before the *Gardenia-Coffea* divergence. The synteny of NMT cluster between *C. canephora* and *Gelsemium sempervirens* showed that the cluster did not exist in this region of *G. sempervirens* genome.


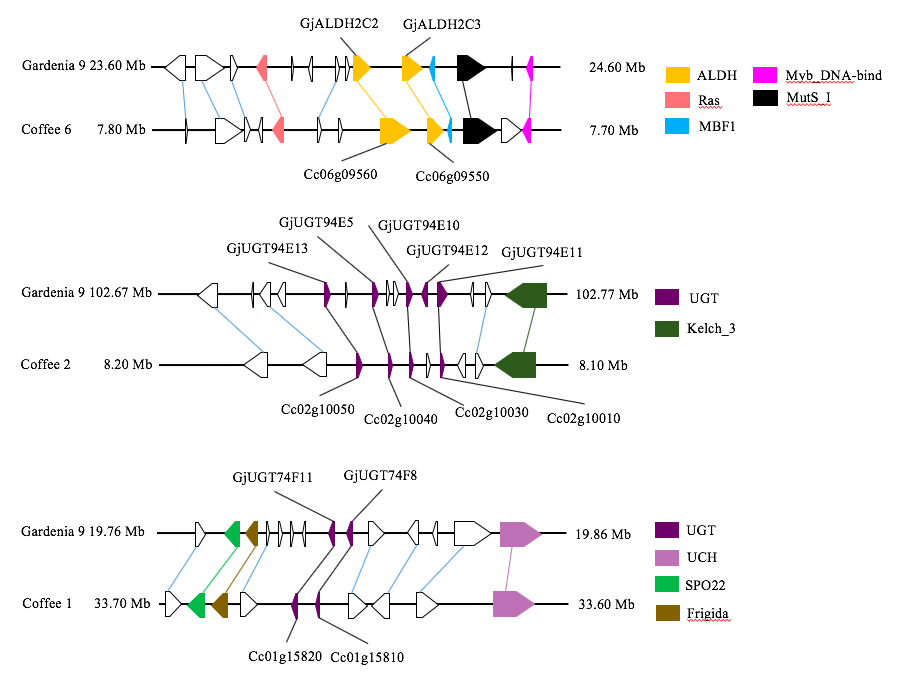
 Additional file 2: Figure S24. A. Microsynteny between *Coffea* and *Gardenia* around *ALD2C*, *UGT94* and *UGT74* genes*.*

Additional file 2: Figure S25. The nucleotide and amino acid sequences for GjCCD4a, GjALDH2C3, GjUGT94E13, and GjUGT74F8.

>GjCCD4a (nt)

ATGGATGCTTTCTCTTCCTCTTTCCTCTCTCGACTATCCCAAAATCCCAAACTTCCGACTTCTCTAACCAACCCTTCATCACCAAATATTCCTTTACTACACATTTCATCCGCTAGAATTGAAGATAAGCCACAACCAACAATTATTACTAGCACCCCCATCACTGCCACAACGCCACCATCATCTAATTCTAATCCCCTTAAGAAGCAGAACCTACCGAAATATACACTTAAAAGATCCAATCCCAAGCCGCAATCATCATCACAACCACCACCATCACTGCAGCTGCAAGAACCTTCAAGAAAGCCAGTAGAACCATCATTGCTCACCAAATTCTTCAATGCACTTGATAACTTCATAGACACATGCATTGATCCTCCAACTCACCCTTCTGTTGACCCCAAATACGTCCTGGCAAACAACTTTGTACCAGTTGATGAACTCCCCCCGACCGAATGTGAGGTGGTGGAAGGCTTTCTTCCACCGTGCCTTGACGGTGCCTACTTCCGCAATGGCCCAAACCCTCAGTTCGTCACTCACGGGCCCTACCACTTTTTCGATGGCGATGGAATGATACATTCCATCAAAATCTCCGAAGGAAAAGCCACCTTCTGCAGCCGGTACGTGAAAACATACAAGTACATGACGGAACGGGATACGGGCTCTCCCGTGTTCCCTAAAGTATTCTCTGGTTTCAATGGCCTAACAGCCTCCGCAGCCAGGGTTGCCCTAGCTGCTGCCAGAATGTTAGCTGGCCAATTTAACCGAGCCAATGGTTTTGGCCTGGCAAACACTAGCTTGGCTCTAGTTGGGGGGAAACTATTCGCTTTAGGTGAACCAGACCTTCCATATGCTGTAAAATTAACACAAACTGGAGATTTAGTAACCGTAGGCCGTCATGACTTTGATGGTAAGCTTATTGCGAGCATGACTGCACATCCCAAAATCGACCCTGAGACAGGTGAAGCTTTCGCATTCAGATACGCGCTAATGCCTCCATTTCTAACCTTCTTTAGAATCAACCCTGATGGTACAAAAGAACCAGACGTCCCGATTTTCTCCATGACACGGCCTTCATTTGTTCATGATTTGGCCATCACAAGAAAATACGCTATTTTTCCTGACATACAAATAGGGATCAACCCTCTTCAGATGATCGCCGGCGGACCACTAGTGGGTTCAGATCCCCGAAAAGTGCCACGACTCGGGGTTATTCCCAGGTATGCTGCGGATGAAGCTGAGATGAAGTGGTTTGATGTTCCGGGACTTAATCTTATGCATGCTGTCAATGCTTGGGATGAAGATGATGGTGATGGTATTGTTGTTGTAGGAACTAATAGTTTGCCCATTGAGCATGTTCTTCGGCGGATGGATTTGGTTCATGCATCCATGGTGAAAATCAAGATTGATCTCAAGACTGGGATGGTTTGGAAACATCCAATTTCTACGAGGAGTATGGAATTTGGAGTCATTAATCCGGCTTATGTTGGCAAGAAGAACAAGTATGTATATGCAGCAGTTGTAGACCCTTTGCCAAAGGTGTCAGGAGTGGTGAAGCTAGACTTATCAGCTTCGGCAGGTGAGCACCGCGACTGCGTCATTGCTAGCCAGCTGTATGAACCGGAGTGCTTTGGAAGCGAACCATTTTTCGTGGCCAAAGACCCTAACAACCCAAATGCAGATGAAGATGATGGTTATGTGGTGTCCTATGTCCATGATGAAAACACAGGAGAGTCAAGATTCTTAGTGATGGACGCGAAGTCACCGAGTCTTGAAATTGTGGCTGCTGTAAAATTGCCTCAAAGGGTTCCTTCTGGCTTTCATGGCCTCTTTGTCAGAGAAAGTGAGCTCAATAAATTGTAA

>GjCCD4a (aa)

MDAFSSSFLSRLSQNPKLPTSLTNPSSPNIPLLHISSARIEDKPQPTIITSTPITATTPPSSNSNPLKKQNLPKYTLKRSNPKPQSSSQPPPSLQLQEPSRKPVEPSLLTKFFNALDNFIDTCIDPPTHPSVDPKYVLANNFVPVDELPPTECEVVEGFLPPCLDGAYFRNGPNPQFVTHGPYHFFDGDGMIHSIKISEGKATFCSRYVKTYKYMTERDTGSPVFPKVFSGFNGLTASAARVALAAARMLAGQFNRANGFGLANTSLALVGGKLFALGEPDLPYAVKLTQTGDLVTVGRHDFDGKLIASMTAHPKIDPETGEAFAFRYALMPPFLTFFRINPDGTKEPDVPIFSMTRPSFVHDLAITRKYAIFPDIQIGINPLQMIAGGPLVGSDPRKVPRLGVIPRYAADEAEMKWFDVPGLNLMHAVNAWDEDDGDGIVVVGTNSLPIEHVLRRMDLVHASMVKIKIDLKTGMVWKHPISTRSMEFGVINPAYVGKKNKGENPPPVGPIVDLSPGVPVVGDGLVGSTRRRRRPGGCCGGPPLVPPGSGIESWGGPQQHPPGRRLRRVLPTRPSPMVGAAYDGYVYAAVVDPLPKVSGVVKLDLSASAGEHRDCVIASQLYEPECFGSEPFFVAKDPNNPNADEDDGYVVSYVHDENTGESRFLVMDAKSPSLEIVAAVKLPQRVPSGFHGLFVRESELNKL*

>GjALDH2C3 (nt)

ATGGCCGTCCAAAGCAACGGCAATGGAAGCTCGGATTCTCATGTTAAGGTTCCTCAGATAAAGTTCACCAAGCTCTTCATCAATGGAGAGTTCGTTGATTCTGTTTCAGGAAAGACGTTTGAGACAATAGATCCAAGAAATGGGGAGGTGATTGCAGAAATTGCCGAGGGGGGCAAGGAAGATGTTGATTTGGCTGTCAAAGCTGCCCGAGAAGCTTTTGACCACGGCCCATGGCCTCGTTTACCTGGCTCTGAGAGGCGAAAGATAATGATGAAGTTTGCAGACTTAATAGATGAAAATACCCAAGAATTGGCCACCTTGGATGCAATTGATGCTGGAAAATTGTTTTACCTTTGCAAGATTATGGATATTCCAGGAGCAGCAGAAACAATTCGCTATTATGCGGGTGCGGCAGATAAGATTCATGGGGAGACTCTAAAAATGTCAACCGCGCTACAAGGATACACATTGCATGAGCCCATTGGAGTTGTTGGACACATCATTCCCTGGAACTTCCCCAGTCAAATGTTTGCTATGAAGGTTGGCCCTGCATTAGCAGCTGGCTGCACCATGGTAGTCAAGCCTGCTGAACAGACGCCTCTGTCAGCCCTCTATTACGCTCATTTAGCAAAGCTGGCTGGAATCCCCGATGGAGTGCTAAACGTTGTAACAGGATTCGGACAAACAGCTGGTGCGGCAATTAGCTCTCATATGGACATTGATATGGTAAGTTTCACGGGTTCTACAGAAGTAGGGCGTCTCGTCATGCAAGCTGCAGCAACAAGCAACTTAAAGCCTGTGTGTCTAGAACTGGGAGGCAAGTCCCCCATTTTAATCTTCGATGATGCAGATGTTGACAAAGTTACAGAACTCGCACTGCAGGGAACTCTATTTAACAAGGGTGAAATATGTGTTGCTGGCTCTCGTATTTTTGTTCAAGAGGGGATTTATGACAAGTTTTTAATCAAGTTGAAGCAGAAGGTGAAGAATTGGGTGGTCGGTGACCCTTTTGATCCAACATCTCATCAAGGACCCCAAGTTGACAAGAAGCAGTACGATAGAATACTTTCATACATCGAGCATGGGAAGAAGGAAGGTGCCACCTTATTCCACGGTGGCAAGCCTTGTGACGGGAAGGGCTACTACATTGAGCCAACAATATTCACTGATGTCACGGATGAGATGACTATTGCGAAGGAAGAAATATTTGGGCCTGTGATGTCAGTATTCAAATTCAAGACTGTTGAGGAGGCAATCAAGAGAGCAAATGCGACCAAATATGGACTGGCAGCGGGCGTCATGACTAACAACATTAACATCGCCAACACCGTCGCAAGATCAATCCGTGCGGGTGCTATCTGGATAAATTGTTACTTTGCCTTCGACAGAGATAGCCCTTACGGAGGCTACAAAATGAGTGGTTTCGGGAGAGACATGGGAATGGATGGCCTCAAGAAGTATCTTGCAGTTAAAGCTGTTGCTACCCCCATTTATAATTCACCTTGGCTGTAA

>GjALDH2C3 (aa)

MAVQSNGNGSSDSHVKVPQIKFTKLFINGEFVDSVSGKTFETIDPRNGEVIAEIAEGGKEDVDLAVKAAREAFDHGPWPRLPGSERRKIMMKFADLIDENTQELATLDAIDAGKLFYLCKIMDIPGAAETIRYYAGAADKIHGETLKMSTALQGYTLHEPIGVVGHIIPWNFPSQMFAMKVGPALAAGCTMVVKPAEQTPLSALYYAHLAKLAGIPDGVLNVVTGFGQTAGAAISSHMDIDMVSFTGSTEVGRLVMQAAATSNLKPVCLELGGKSPILIFDDADVDKVTELALQGTLFNKGEICVAGSRIFVQEGIYDKFLIKLKQKVKNWVVGDPFDPTSHQGPQVDKKQYDRILSYIEHGKKEGATLFHGGKPCDGKGYYIEPTIFTDVTDEMTIAKEEIFGPVMSVFKFKTVEEAIKRANATKYGLAAGVMTNNINIANTVARSIRAGAIWINCYFAFDRDSPYGGYKMSGFGRDMGMDGLKKYLAVKAVATPIYNSPWL*

>GjUGT94E13 (nt)

ATGAAGGTACTAATGTTGCCATGGTTAGCTCATGGTCACATCTCTCCTTTCTTAGAGCTAGCCAAGAGGCTGGCAAAGAAAAACTTCCACATCTACCTGTGTTCCACCTCTGTCAATCTCAGCTCCATCAAGAACAAGATTACCGGAGAATATTCCGACTCAATAGAACCAGTTGAGCTACAGCTTCCATGCTTGCCTGATCTTCCTCCTCATTACCACACCACCAATGGCCTCCCACCCCATCTCATGACCACCCTCAAAACAGCCTATGAACTGTCAGCTCCCGACTTTTCTAATATCCTCACCGCTCTGCATCCTGACTTGGTTGTGTACGACTTTAACCAGCCGTGGGCAGCAGAAATTGCTTCGTCTAAAAACATCCCAGCAGTTCAATTTCTCCCGGTTGGGGCAACAATGATGGCTTTCAGTCTGCACATGCTCAAGTATTCTGGAAAAGAATTCCCCCACCCAGAAATTTATATCCGGGACTACGAGATGCTCAAGTTTCAGTCAAGGAACGATCAAGTTAATGATGTTAGTGATAGAGAAAGAATCCTGCAAGCTCTGGACCTGTCGTGTAAGATTTTGTTGGTCAAGTCGTTCAAAGAAATCGAAGAAAAATTCATGAATACTCTCTCTGTTGCATCTGGTAAAAAGGTGGTCCCCGTTGGCCCTCTTGTCCAAGACGTCAATATCGATGACATTCAAGATGAGGAAATGGAAATCATCCATTGGCTTGACCAAAAGGAAAATGCTTCAGTGGTGTTTGTCTCTTTTGGTAGTGAGTATTTCTTGACTAAGGAGGAAAGAAACGAGATTGCTCGGGGTCTTGAGCTCAGCAATGTTAACTTTATATGGGTAATTAGGTTCCCTCTGGGAGGAAAAATAACTATGGAAGAAGCATTGCCCGAAGGTTTTCTTGAGAGAGTGGGAGATAGAGGAAAAATTGTGGATGGATGGGCTCCTCAGGCAAGAATTTTGAAGCATGCGAATACTGGTGCTTTTTTGAGCCATTGTGGATGGAGTTCAATGATGGAGAGTATGAAATTTGGTGTTCCAATAATAGCTATGCCTATGAGTGTTGATCAACCTGTGAATGCTAGATTGATTGAAGCAGTTGGAGTGGGATTGGAGCCCTTGAGAGACGAGAAGGGGAATCTTCAAAGTGCGGAGATTGCCAAAGTGATTAGAAAGGTTTTGGTGGACGAAAGTGGAGAAAATGTGAGGAGGAAGGCTAAGGAATTGAGTGAACAAATGGAAATGAGAGGTGACGAGGAGGAGATTGATAACCTTGTAGAGGAGCTGCTGCAACTCTGCCGAAAGAACAACGGGGGCTGTTAA

>GjUGT94E13 (aa)

MKVLMLPWLAHGHISPFLELAKRLAKKNFHIYLCSTSVNLSSIKNKITGEYSDSIEPVELQLPCLPDLPPHYHTTNGLPPHLMTTLKTAYEMSAPDFSNILTALHPDLVVYDFNQPWAAEIASSKNIPAVQFLPVGATMMAFSLHMLKYSGKEFPHPEIYIRDYEMLKFQSRNDQVNDVSDRERILQALDLSCKILLVKSFKEIEEKFMNTLSVASGKKVVPVGPLVQDVNIDDIQDEEMEIIHWLDQKENASVVFVSFGSEYFLTKEERNEIAWGLELSNVNFIWVIRFPLGGKITMEEALPEGFLERVGDRGKIVDGWAPQARILKHANTGAFLSHCGWSSMMESMKFGVPIIAMPMSVDQPVNARLIEAVGVGLEPLRDEKGNLQSAEIAKVIRKVLVDESGENVRRKAKELSEQMEMRGDEEEIDNLVEELLQLCRKNNGGC*

>GjUGT74F8 (nt)

ATGAATTCCAGCAAAGTTCATGTTTTAGTTCTGCCATATCCTGCCCAGGGCCACATAAACCCCTTGCTCCAGTTCTGTAAACGTTTGGTGGCAAAAGGTGTCAAAACCACGTTTGTCAACACTGTCTTCGTCTCCAAGTCCATTCAACCAGATCCCAGCTGCTCAATTAACTTTGAGACCATTTCAGATGGCCATGACGAAGGTGGCTATGCAGCAGCTGAAAGCCCAGAGGCCTATCTTGTAGAACTGGCCACCTTCGGCTCAAAGACGTTGGCTGATCTTATCAGGAAGCTTGAAGACAAGGGCGAACCAGTCCAGGCCGTGATTTATGATTCATTCCTGACATGGGCTCTTGATGTAGCTAAGCAATTTGGACTTGTTACAGCTGCTTTTTTCACTCAGACTTGTGCTGTAAACAGCGTCTACTACCATGTTTATCATGGACTTCTACCGCTCCCTCTGTCAGACTCACCTCTTTCACTTCCTGGATTGCCGCTGCTCCAACCCAAGGAAACTCCGTCCTTTGTTTACCTTCCCGATTCGTATCCTGCTTTTCGTCTCATGCTCGTTAATCAGTTCTCCAACGTTGATCAAGCAGATTGGGTCATCCTCAACATTTTCCACAAATTGGAGGAAGATGTGCTGAATTGGATAGCGAAACTTTGGCGAGTGATTACAGTCGGTCCAACGGTTCCATCCATGTACTTGGATAAACGCCTTGAGGACGATAAATGTTACGGAATAAATCTCTTCAAGCCAGACTCCTGCCTGTGCATGAACTGGCTAAATAATCAACCAAAGGACTCGGTAGTTTATGTATCATTCGGTAGCTGGACAGAAATTGACGTAAAACAAGTGGAGGAAATAGCTTCAGCTTTGAAGGAAAGCGGATTCAAATTCTTGTGGGTCGTGAGATCATTTGAGAAGGAAAAGCTTCCAAGCAACTTCGCTGAGGAGACGTCGGAGAAAGGCTTGGTGGTGACATGGAGTCCACAGCTTGAGGTATTGGCACATGAATCCGTCGCTTGTTTCGTCACTCATTGCGGGTTCAATTCAGTGCTGGAGGCGTTGAGTTTAGGGGTGCCGGTGGTGGCCGCGCCGCAGTGGACGGACCAGCCCACCAACGCTAAGTTTCTGGAGGATATTTGGGGAGTTGGTGTGAAGGCTGGTGCGGATGAGAAAGGAATTGTGACGAGGGAAACGCTAGTTTCCTGCATAAGGGAGATTATGGAGGGAGAAAAAGGAAAGCAGATTAAAGAGAATGCCATTAAATGGAAGACTTTAGCCAAAGAAGCAGTTGATGAGGGCGGAAGTTCTGATAAGAACATTGACGAATTTGTTGCAGAATTGGCTGGCCAGAAGGCCCAGAACTAG

>GjUGT74F8 (aa)

MMNSSKVHVLVLPYPAQGHINPLLQFCKRLVAKGVKTTFVNTVFVSKSIQPDPSCSINFETISDGHDEGGYAAAESPEAYLVELATFGSKTLADLIRKLEDKGEPVQAVIYDSFLTWALDVAKQFGLVTAAFFTQTCAVNSVYYHVYHGLLPLPLSDSPLSLPGLPLLQPKETPSFVYLPDSYPAFRLMLVNQFSNVDQADWVILNIFHKLEEDVLNWIAKLWRVITVGPTVPSMYLDKRLEDDKCYGINLFKPDSCLCMNWLNNQPKDSVVYVSFGSWTEIDVKQVEEIASALKESGFKFLWVVRSFEKEKLPSNFAEETSEKGLVVTWSPQLEVLAHESVACFVTHCGFNSVLEALSLGVPVVAAPQWTDQPTNAKFLEDIWGVGVKAGADEKGIVTRETLVSCIREIMEGEKGKQIKENAIKWKTLAKEAVDEGGSSDKNIDEFVAELAGQKAQN*


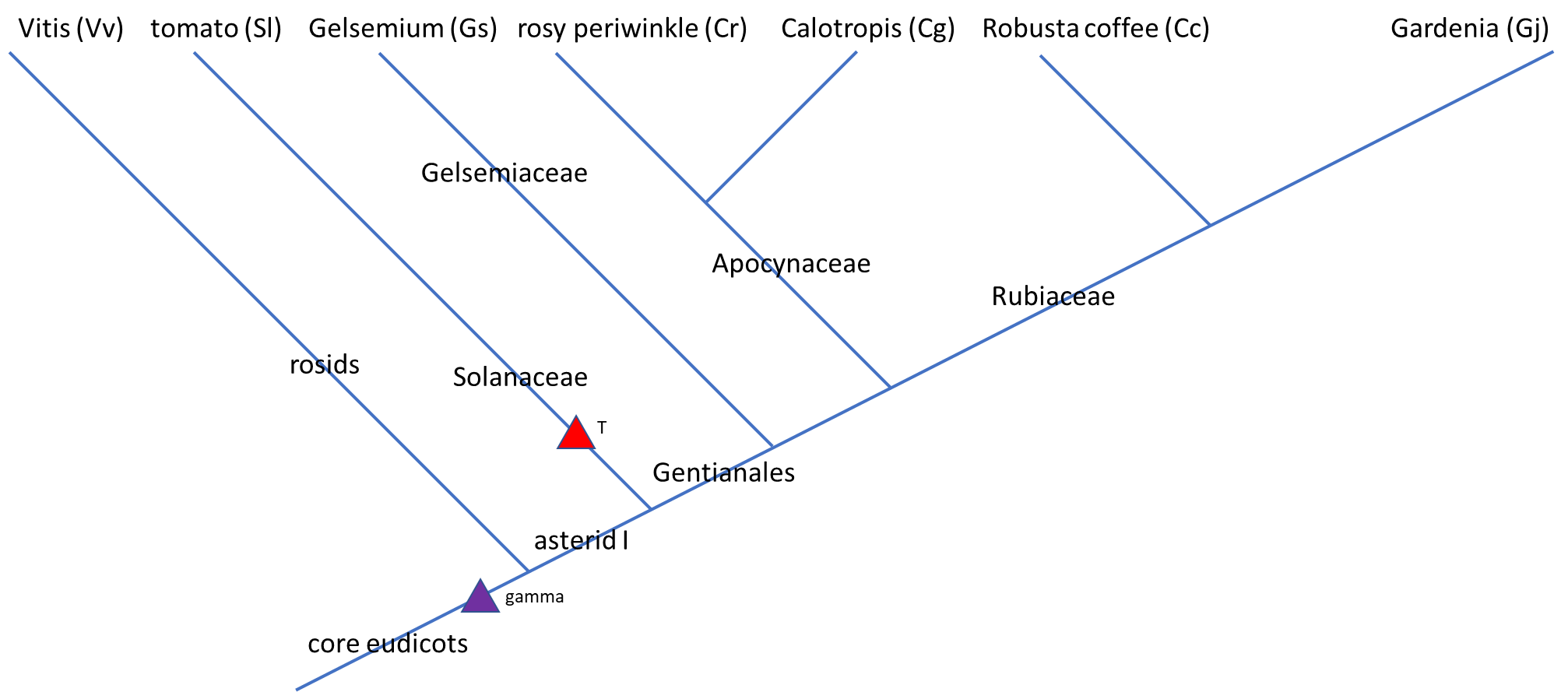


Additional file 2: Figure S26. Phylogenetic tree of candidate species for the evolution analysis of NMTs, CCDs, UGTs, and ALDHs presented in this study.

Additional file 2: Table S1. Illumina sequencing of paired-end and mate-pair libraries.

| Library | Insert size | Clean reads | Base pairs | Coverage (×) |
| --- | --- | --- | --- | --- |
| PE_lib1 | 250 bp | 319,216,178 | 46,937,618,600 | 85 |
| PE_lib 2 | 500 bp | 278,358,794 | 41,075,029,576 | 75 |
| MP_lib 3 | 2 kb | 217,234,616 | 21,506,227,182 | 39 |
| MP_lib 4 | 5 kb | 204,381,020 | 20,2337,21,178 | 37 |
| MP_lib 5 | 10 kb | 126,616,828 | 12,535,066,170 | 23 |
| MP_lib 6 | 20 kb | 189,273,492 | 18,738,075,906 | 34 |
| Total |  | 1,335,080,928 | 161,025,738,612 | 293 |

Additional file 2: Table S2. ONT sequencing data.

| Libraries | Total Reads Bases | Total Reads Number | Pass Reads Bases | Pass Reads Number | Pass Reads Mean Length | Pass Reads N50 Length | Pass Reads Medium Length | Pass Reads Max Length | Pass Reads Avg Score |
| --- | --- | --- | --- | --- | --- | --- | --- | --- | --- |
| ONT1 | 6,767,747,724 | 611,577 | 6,308,205,834 | 455,254 | 13,856 | 19,970 | 6,015 | 130,015 | 10.56 |
| ONT2 | 5,535,387,510 | 436,524 | 5,274,624,738 | 335,672 | 15,713 | 22,094 | 6,981 | 125,850 | 10.13 |
| ONT 3 | 7,804,504,233 | 623,628 | 7,432,584,475 | 481,948 | 15,421 | 21,616 | 6,881 | 113,514 | 10.18 |
| ONT 4 | 5,608,557,039 | 445,714 | 5,329,121,913 | 347,791 | 15,322 | 21,317 | 13,647 | 366,834 | 9.96 |
| ONT 5 | 5,072,285,590 | 400,113 | 4,843,256,471 | 322,166 | 15,033 | 21,140 | 6,643 | 95,879 | 10.04 |
| ONT 6 | 1,611,735,102 | 157,974 | 1,527,554,334 | 89,716 | 17,026 | 23,168 | 7,715 | 104,414 | 10.62 |

Additional file 2: Table S3. *G. jasminoides* genome assembly statistics.

|  | ALLPATHS-LG | Canu | Minimap/Miniasm | Canu+  SMARTdenovo | Canu+  SMARTdenovo+  Pilon 3× | Canu+  SMARTdenovo+  Pilon 3×+  Purge Haplotigs | Canu+  SMARTdenovo+  Pilon 3×+  Purge Haplotigs+ Hi-C |
| --- | --- | --- | --- | --- | --- | --- | --- |
| Input Data | HiSeq | ONT | ONT | ONT | ONT+HiSeq | ONT+HiSeq+  ONT | ONT+HiSeq+  ONT+Hi-C |
| Genome Size (bp) | 635,613,314 | 838,534,178 | 553,533,477 | 669,809,995 | 677,871,174 | 534,116,817 | 534,975,817 |
| Contigs | - | 6213 | 3465 | 2006 | 2006 | 940 | - |
| Contig N50 (bp) | - | 269,719 | 224,879 | 691,101 | 700,746 | 1,028,321 | - |
| Largest Contig (bp) | - | 4,466,419 | 1,640,736 | 11,561,164 | 11,656,183 | 11,656,183 | - |
| Scaffolds | 58,859 | - | - | - | - | - | **11 Chromosomes+**  **68 unordered contigs+**  **2 unclustered contigs** |
| Scaffolds N50 (bp) | 60,601 | - | - | - | - | - | 43,900,188 |
| Largest Scaffold (bp) | 874,432 | - | - | - | - | - | 110,581,163 |
| N bases in scaffolds (%) | 27.7 | 0 | 0.01 | 0 | 0 | 0 | 0.16% |
| Completeness BUSCOs | 60.8% | 41.8% | - | - | 96.1% | 95.0% | 95.0% |
| complete single-copy BUSCOs |  |  |  |  | 91.9% | 92.7% | 92.7% |
| complete duplicated BUSCOs |  |  |  |  | 4.2% | 2.3% | 2.3% |

Additional file 2: Table S4. Genome annotation of different *G. jasminoides* assemblies

| **Annotation** | **ONT-based assembly** | **Illumina-based assembly** |
| --- | --- | --- |
| No. of predicted gene loci | 35 954 | 35 769 |
| No. of predicted transcripts and proteins | 35 967 | 43 527 |
| Average gene length (bp) | 978 | 807 |
| GC content (%) | 43.67 | 42.92 |
| Percentage of whole gene length in genome (%) | 6.58 | 4.55 |
| Masked repeat sequence length (bp) | 288,723,343 | 245,721,735 |
| Percentage of sequence repeats in genome (%) | 53.97 | 36.51 |

Additional file 2: Table S5. Annotation of *G. jasminoides* transposable elements (TEs).

| Repeat Class | Elements number | Length occupied (bp) | Percentage of sequence |
| --- | --- | --- | --- |
| Retrotransposon | 460,912 | 283,300,793 | 52.96 % |
|  |  |  |  |
| RNA transposable elements | 178,718 | 192,469,660 | 35.98% |
| non-LTR(Long terminal repeat) | 18,137 | 12,801,856 | 2.39% |
| LINE | 17,562 | 12,624,690 | 2.36 % |
| SINE | 575 | 177,166 | 0.03 % |
| LTR(Long terminal repeat) | 160,581 | 179,667,804 | 33.58 % |
| Gypsy | 107,040 | 120,783,441 | 22.58% |
| Copia | 29,087 | 36,916,960 | 6.90% |
| Other | 24,454 | 21,967,403 | 4.10% |
|  |  |  |  |
| DNA elements | 54,698 | 20,584,841 | 3.85 % |
|  |  |  |  |
| Unclassified TEs | 227,496 | 70,246,292 | 13.13 % |
|  |  |  |  |
| Small RNA | 76 | 5308 | 0.00 % |
|  |  |  |  |
| Satellites | 169 | 35,556 | 0.01 % |
| Simple repeats | 110,858 | 5,106,989 | 0.95 % |
| Low complexity | 20,868 | 965,003 | 0.18 % |
|  |  |  |  |
| Total Repeats | 592,883 | 288,723,343 | 53.97% |

Additional file 2: Table S6. Identification of simple sequence repeats (SSR) in the *G. jasminoides* genome.

| Types | Unit size | Cut-off | Number of SSR |
| --- | --- | --- | --- |
| Monomer | 1 | 10 | 195,517 |
| Dimer | 2 | 6 | 59,140 |
| Trimer | 3 | 5 | 17,869 |
| Tetramer | 4 | 5 | 4,548 |
| Pentamer | 5 | 5 | 930 |
| Hexamer | 6 | 5 | 428 |
| Total |  |  | 278,432 |

Additional file 2: Table S7. Annotation of *G. jasminoides* non-coding RNAs.

| ncRNA type | #Loci | Average length (bp) | Total length (bp) |
| --- | --- | --- | --- |
| miRNA | 106 | 78 | 13,320 |
| tRNA | 425 | 71 | 30,097 |
| rRNA(5S) | 21 | 112 | 2,250 |
| rRNA(18s) | 8 | 1,760 | 14,077 |
| rRNA(5.8s) | 8 | 154 | 1,232 |
| rRNA(28s) | 8 | 6,319 | 50,555 |
| snoRNA | 150 | 100 | 15,117 |
| snRNA | 75 | 164 | 12,280 |

Additional file 2: Table S8. Annotation of *G. jasminoides* rRNAs.

| **Chromosome** | **Start** | **End** | **Length** |  | **Type** |
| --- | --- | --- | --- | --- | --- |
| Gardenia11 | 9328246 | 9328359 | 113 | - | 5s_rRNA |
| Gardenia11 | 16633314 | 16633427 | 113 | - | 5s_rRNA |
| Gardenia11 | 13703143 | 13703257 | 114 | + | 5s_rRNA |
| Gardenia9 | 101459215 | 101459328 | 113 | + | 5s_rRNA |
| Gardenia11 | 16637281 | 16637376 | 95 | - | 5s_rRNA |
| Gardenia1 | 15642868 | 15642982 | 114 | + | 5s_rRNA |
| Gardenia11 | 16633996 | 16634115 | 119 | - | 5s_rRNA |
| Gardenia11 | 16634718 | 16634832 | 114 | - | 5s_rRNA |
| Gardenia11 | 16636501 | 16636605 | 104 | - | 5s_rRNA |
| Gardenia11 | 9829140 | 9829254 | 114 | + | 5s_rRNA |
| Gardenia9 | 77293479 | 77293591 | 112 | + | 5s_rRNA |
| Gardenia11 | 16674727 | 16674841 | 114 | - | 5s_rRNA |
| Gardenia11 | 16632600 | 16632714 | 114 | - | 5s_rRNA |
| Gardenia11 | 16629086 | 16629201 | 115 | - | 5s_rRNA |
| Gardenia11 | 16635864 | 16635978 | 114 | - | 5s_rRNA |
| Gardenia11 | 16635430 | 16635544 | 114 | - | 5s_rRNA |
| Gardenia11 | 15973186 | 15973298 | 112 | + | 5s_rRNA |
| Gardenia9 | 29796415 | 29796528 | 113 | - | 5s_rRNA |
| Gardenia11 | 16642388 | 16642502 | 114 | - | 5s_rRNA |
| Gardenia11 | 16628376 | 16628490 | 114 | - | 5s_rRNA |
| Gardenia2 | 10344657 | 10344771 | 114 | + | 5s_rRNA |
| Gardenia10 | 5903651 | 5905447 | 1796 | + | 18s_rRNA |
| Gardenia10 | 5905640 | 5905790 | 154 | + | 5.8S_rRNA |
| Gardenia10 | 5905641 | 5912627 | 6986 | + | 28s_rRNA |
| Gardenia10 | 5912594 | 5914367 | 1773 | + | 18s_rRNA |
| Gardenia10 | 5914606 | 5914761 | 154 | + | 5.8S_rRNA |
| Gardenia10 | 5914607 | 5921685 | 7078 | + | 28s_rRNA |
| Gardenia10 | 5921652 | 5923417 | 1765 | + | 18s_rRNA |
| Gardenia10 | 5923592 | 5923738 | 154 | + | 5.8S_rRNA |
| Gardenia10 | 5923593 | 5929257 | 5664 | + | 28s_rRNA |
| Gardenia10 | 5929224 | 5930986 | 1762 | + | 18s_rRNA |
| Gardenia10 | 5931169 | 5931324 | 154 | + | 5.8S_rRNA |
| Gardenia10 | 5931170 | 5937383 | 6213 | + | 28s_rRNA |
| Gardenia10 | 5937350 | 5939063 | 1713 | + | 18s_rRNA |
| Gardenia10 | 5939241 | 5939389 | 154 | + | 5.8S_rRNA |
| Gardenia10 | 5939242 | 5945817 | 6575 | + | 28s_rRNA |
| Gardenia10 | 5945784 | 5947532 | 1748 | + | 18s_rRNA |
| Gardenia10 | 5947714 | 5947861 | 154 | + | 5.8S_rRNA |
| Gardenia10 | 5947715 | 5954094 | 6379 | + | 28s_rRNA |
| Gardenia10 | 5954061 | 5955820 | 1759 | + | 18s_rRNA |
| Gardenia10 | 5956012 | 5956157 | 154 | + | 5.8S_rRNA |
| Gardenia10 | 5956013 | 5962665 | 6652 | + | 28s_rRNA |
| Gardenia10 | 5962632 | 5964393 | 1761 | + | 18s_rRNA |
| Gardenia10 | 5964580 | 5964728 | 154 | + | 5.8S_rRNA |
| Gardenia10 | 5964581 | 5969589 | 5008 | + | 28s_rRNA |

Additional file 2: Table S9. Enrichment of UDP-glucoronosyl and UDP-glucosyl transferase, and glycoside hydrolase family members in *Gardenia*-specific and -expanded gene families.

| PFAM annotation | PFAM ID | Gene ID | E value |
| --- | --- | --- | --- |
| UDPGT | PF00201.17 | Gj7A355T79.1 | 2.70E-13 |
| UDPGT | PF00201.17 | Gj7A356T49.1 | 1.60E-15 |
| UDPGT | PF00201.17 | Gj11X147T4.1 | 1.60E-16 |
| UDPGT | PF00201.17 | Gj11X234T8.1 | 3.00E-17 |
| UDPGT | PF00201.17 | Gj11X236T42.1 | 1.80E-18 |
| UDPGT | PF00201.17 | Gj11A290T115.1 | 2.20E-16 |
| UDPGT | PF00201.17 | Gj5A305T50.1 | 4.00E-19 |
| UDPGT | PF00201.17 | Gj10P221T20.1 | 1.00E-17 |
| UDPGT | PF00201.17 | Gj10P221T21.1 | 7.10E-20 |
| Glyco_hydro_1 | PF00232.17 | Gj11A44T31.1 | 1.90E-29 |
| Glyco_hydro_3_C | PF01915.21 | Gj11A66T20.1 | 1.20E-23 |
| Glyco_hydro_3_C | PF01915.21 | Gj11A68T14.1 | 2.40E-23 |
| Glyco_hydro_1 | PF00232.17 | Gj6P280T10.1 | 2.20E-110 |
| Glyco_hydro_1 | PF00232.17 | Gj6P280T10.1 | 2.20E-110 |
| Glyco_hydro_19 | PF00182.18 | Gj3P101T4.1 | 6.00E-13 |
| Glyco_hydro_19 | PF00182.18 | Gj3P148T3.1 | 1.40E-12 |
| Glyco_hydro_1 | PF00232.17 | Gj1P51T23.1 | 2.00E-94 |
| Glyco_hydro_1 | PF00232.17 | Gj1P51T23.1 | 2.00E-94 |
| Glyco_hydro_1 | PF00232.17 | Gj1P51T23.1 | 2.00E-94 |
| Glyco_hydro_1 | PF00232.17 | Gj1P51T23.1 | 2.00E-94 |
| Glyco_hydro_1 | PF00232.17 | Gju18967P0T3.1 | 4.50E-73 |
| Glyco_hydro_1 | PF00232.17 | Gju18967P0T3.1 | 4.50E-73 |
| Glyco_hydro_1 | PF00232.17 | Gj5A116T47.1 | 4.40E-63 |
| Glyco_hydro_1 | PF00232.17 | Gj5A116T47.1 | 4.40E-63 |
| Glyco_hydro_1 | PF00232.17 | Gj5A117T34.1 | 1.70E-105 |
| Glyco_hydro_1 | PF00232.17 | Gj5A118T54.1 | 1.90E-109 |
| Glyco_hydro_1 | PF00232.17 | Gj5P151T4.1 | 2.00E-74 |
| Glyco_hydro_1 | PF00232.17 | Gj5P151T4.1 | 2.00E-74 |
| Glyco_hydro_1 | PF00232.17 | Gj5P151T4.1 | 2.00E-74 |
| Glyco_hydro_17 | PF00332.17 | Gj5X296T25.1 | 1.70E-88 |
| Glyco_hydro_17 | PF00332.17 | Gj5X296T34.1 | 3.00E-89 |
| Glyco_hydro_1 | PF00232.17 | Gj5P368T9.1 | 8.20E-110 |
| Glyco_hydro_1 | PF00232.17 | Gj2A411T68.1 | 1.00E-07 |
| Glyco_hydro_1 | PF00232.17 | Gj2P411T6.1 | 6.90E-17 |
| Glyco_hydro_18 | PF00704.27 | Gj9X803T21.1 | 1.30E-24 |
| Glyco_hydro_18 | PF00704.27 | Gj9P803T10.1 | 2.40E-24 |
| Glyco_hydro_18 | PF00704.27 | Gj9X804T17.1 | 7.00E-22 |
| Glyco_hydro_1 | PF00232.17 | Gj4A172T9.1 | 3.70E-34 |
| Glyco_hydro_1 | PF00232.17 | Gj4A172T9.1 | 3.70E-34 |
| Glyco_hydro_1 | PF00232.17 | Gj4A173T60.1 | 3.90E-24 |
| Glyco_hydro_1 | PF00232.17 | Gj4P403T3.1 | 3.70E-49 |
| Glyco_hydro_1 | PF00232.17 | Gj4P403T3.1 | 3.70E-49 |
| Glyco_hydro_1 | PF00232.17 | Gj4P403T3.1 | 3.70E-49 |
| Glyco_hydro_1 | PF00232.17 | Gj4A482T70.1 | 5.30E-92 |
| Glyco_hydro_1 | PF00232.17 | Gj4A483T61.1 | 1.40E-110 |
| Glyco_hydro_1 | PF00232.17 | Gj4A483T61.1 | 1.40E-110 |
| Glyco_hydro_1 | PF00232.17 | Gj4A483T63.1 | 3.00E-110 |
| Glyco_hydro_1 | PF00232.17 | Gj4A483T63.1 | 3.00E-110 |

Additional file 2: Table S10. *CCD* genes identified in different plant genomes.

|  | *Gardenia jasminoides* | *Arabidopsis thaliana* | *Coffea canephora* | *Vitis vinifera* | *Capsicum annuum* | *Malus domestica* | *Glycine max* | *Solanum*  *lycopersicum* | *Gelsemium sempervirens* | *Catharanthus roseus* | *Calotropis gigantea* |
| --- | --- | --- | --- | --- | --- | --- | --- | --- | --- | --- | --- |
| CCD1 | 4 | 1 | 3 | 2 | 1 | 3 | 2 | 1 | 0 | 1 | 1 |
| CCD2 | 0 | 0 | 0 | 0 | 0 | 0 | 0 | 0 | 0 | 0 | 0 |
| CCD4 | 4 | 1 | 1 | 3 | 2 | 5 | 1 | 2 | 1 | 3 | 1 |
| CCD7 | 1 | 1 | 1 | 1 | 1 | 2 | 1 | 0 | 1 | 1 | 1 |
| CCD8 | 3 | 1 | 2 | 1 | 1 | 3 | 2 | 1 | 1 | 1 | 1 |
| NCEDs | 2 | 5 | 2 | 3 | 3 | 4 | 2 | 3 | 4 | 3 | 3 |
| Total | 14 | 9 | 9 | 10 | 11 | 21 | 8 | 7 | 7 | 9 | 7 |

Additional file 2: Table S11. *CCD* gene expression (FPKM values) in *G. jasminoides* tissues.

| Chromosome | Gene name | Gene ID | Root | Stem | Leaf | Flower | Fruitlet | Green Fruit | Red Fruit |
| --- | --- | --- | --- | --- | --- | --- | --- | --- | --- |
| Gardenia2 | CCD1 | Gj2A450T56 | 39.20 | 97.02 | 340.49 | 464.54 | 119.10 | 209.05 | 204.66 |
| Gardenia9 | CCD1L1 | Gj9A304T15 | 0.00 | 0.00 | 0.00 | 0.00 | 0.00 | 0.00 | 0.00 |
| Gardenia9 | CCD1L2 | Gj9A305T8 | 0.00 | 0.00 | 0.00 | 0.00 | 0.00 | 0.00 | 0.00 |
| Gardenia9 | CCD1L3 | Gj9A305T9 | 0.00 | 0.00 | 0.00 | 0.00 | 0.00 | 0.00 | 0.00 |
| Gardenia9 | CCD4a* | Gj9A597T69 | 0.00 | 0.00 | 0.00 | 1567.62 | 0.09 | 1861.45 | 2071.86 |
| Gardenia9 | CCD4b | Gj9A597T68 | 0.00 | 0.00 | 0.00 | 2.75 | 0.00 | 0.00 | 0.04 |
| Gardenia9 | CCD4c | Gj9A597T67 | 0.68 | 15.67 | 0.93 | 5672.04 | 1.08 | 0.00 | 0.12 |
| Gardenia9 | CCD4d | Gj9P597T6 | 4.18 | 31.43 | 126.76 | 2794.84 | 79.25 | 12.97 | 6.62 |
| Gardenia2 | CCD7 | Gj2A358T79 | 0.12 | 0.21 | 0.00 | 0.00 | 0.07 | 0.00 | 0.08 |
| Gardenia9 | CCD8a | Gj9A301T69 | 0.21 | 0.33 | 0.07 | 0.07 | 0.00 | 0.00 | 0.18 |
| Gardenia9 | CCD8b | Gj9A301T70 | 3.22 | 0.84 | 0.03 | 0.00 | 0.05 | 0.11 | 0.09 |
| Gardenia9 | CCD8c | Gj9A301T73 | 0.29 | 0.54 | 0.00 | 0.00 | 0.00 | 0.00 | 0.00 |
| Gardenia7 | NCED3 | Gj7X408T57 | 188.42 | 147.31 | 49.39 | 26.19 | 93.06 | 17.88 | 6.10 |
| Gardenia9 | NCED6 | Gj9X759T23 | 0.11 | 0.00 | 0.00 | 0.08 | 3.74 | 2.79 | 0.50 |

*Candidate CCD gene for crocetin dialdehyde biosynthesis, characterized in this study.

Additional file 2: Table S12. *ALDH* gene expression (FPKM values) in *G. jasminoides* tissues.

| Chromosome | Gene ID | Gene name | Root | Stem | Leaf | Flower | Fruitlet | Green Fruit | Red Fruit | Pearson correlation coefficient with *GjCCD4a* |
| --- | --- | --- | --- | --- | --- | --- | --- | --- | --- | --- |
| Gardenia6 | Gj6A205T30.1 | GjALDH10A9 | 160.53 | 154.28 | 137.69 | 118.12 | 150.57 | 174.31 | 165.98 | 0.17 |
| Gardenia4 | Gj4P472T2.1 | GjALDH11A3 | 57.98 | 457.91 | 1194.29 | 37.49 | 460.04 | 106.57 | 120.91 | -0.57 |
| Gardenia6 | Gj6P19T11.1 | GjALDH12A1 | 10.80 | 22.15 | 18.40 | 32.72 | 13.40 | 31.40 | 21.90 | 0.74 |
| Gardenia9 | Gj9P1049T24.1 | GjALDH18B1 | 54.62 | 29.29 | 46.32 | 13.81 | 60.13 | 36.96 | 45.12 | -0.44 |
| Gardenia3 | Gj3P286T12.1 | GjALDH22A1 | 10.52 | 23.38 | 17.02 | 6.86 | 19.77 | 11.97 | 15.44 | -0.52 |
| Gardenia11 | Gj11A422T108.1 | GjALDH2B1(ALDH14) | 201.94 | 554.82 | 13.94 | 64.89 | 386.33 | 273.64 | 412.87 | -0.03 |
| Gardenia6 | Gj6A242T55.1 | GjALDH2B2 | 34.58 | 312.48 | 767.51 | 29.42 | 251.73 | 102.78 | 126.70 | -0.51 |
| Gardenia6 | Gj6A297T86.1 | GjALDH2C1 | 0.00 | 0.00 | 0.00 | 0.00 | 0.00 | 0.00 | 0.00 |  |
| Gardenia9 | Gj9A24T69.1 | GjALDH2C2 | 20.19 | 10.16 | 0.61 | 15.47 | 14.37 | 160.86 | 116.80 | 0.80 |
| Gardenia9 | Gj9A24T70.1 | GjALDH2C3(ALDH12)* | 96.18 | 13.66 | 106.67 | 1686.88 | 101.18 | 642.04 | 849.59 | 0.78 |
| Gardenia6 | Gj6A297T87.1 | GjALDH2C4 | 1.19 | 0.77 | 0.29 | 38.97 | 5.16 | 34.98 | 21.06 | 0.89 |
| Gardenia10 | Gj10P294T12.1 | GjALDH3F1 | 18.90 | 14.76 | 13.67 | 80.51 | 23.75 | 25.73 | 43.74 | 0.64 |
| Gardenia8 | Gj8P92T6.1 | GjALDH3H1 | 33.82 | 98.19 | 120.25 | 489.90 | 85.79 | 146.38 | 147.13 | 0.52 |
| Gardenia9 | Gj9A149T108.1 | GjALDH5F1 | 107.67 | 51.49 | 49.99 | 63.59 | 68.34 | 55.15 | 51.67 | -0.35 |
| Gardenia9 | Gj9A894T52.1 | GjALDH6B1 | 37.61 | 32.94 | 60.62 | 19.08 | 34.94 | 26.91 | 27.57 | -0.66 |
| Gardenia11 | Gj11P26T2.1 | GjALDH6B2 | 94.10 | 56.12 | 61.31 | 131.71 | 60.93 | 85.54 | 96.51 | 0.65 |
| Gardenia10 | Gj10P264T10.1 | GjALDH6B3 | 8.09 | 12.21 | 5.52 | 13.90 | 13.06 | 6.70 | 7.04 | -0.17 |
| Gardenia9 | Gj9P844T7.1 | GjALDH7B4 | 34.62 | 44.19 | 69.29 | 210.94 | 69.95 | 117.35 | 132.23 | 0.79 |
| Gardenia9 | Gj9A597T69 | GjCCD4a | 0.00 | 0.00 | 0.00 | 1567.62 | 0.09 | 1861.45 | 2071.86 | 1.00 |

*Candidate ALDH gene for crocetin biosynthesis, characterized in this study. The genes in brackets are the ALDHs identified as candidate genes in a previous study

(Ji et al., 2017).

Additional file 2: Table S13. *UGT* gene expression (FPKM values) in *G. jasminoides* tissues.

| Chromosome | Gene ID | Gene name | Root | Stem | Leaf | Flower | Fruitlet | Green Fruit | Red Fruit |  | Significant expression |
| --- | --- | --- | --- | --- | --- | --- | --- | --- | --- | --- | --- |
| Gardenia 9 | Gj9A198T90* | UGT74F8 | 21.95 | 12.17 | 11.54 | 4.05 | 121.34 | 1853.29 | 1621.71 | Cluster30 | Y |
| Gardenia 9 | Gj9P1027T10* | UGT94E13 (UGT60) | 3.31 | 1.06 | 0.46 | 3026.19 | 5.72 | 682.70 | 807.14 | Cluster28 | Y |
| Gardenia 7 | Gj7P357T4* | UGT71A11 | 0.30 | 0.03 | 2.63 | 8.15 | 5.40 | 220.47 | 668.44 | Cluster25 | Y |
| Gardenia 8 | Gj8A384T85* | UGT85A8 | 0.77 | 0.17 | 18.65 | 9.90 | 4.49 | 50.89 | 389.84 | Cluster27 | Y |
| Gardenia 10 | Gj10A251T58* | UGT85A2 | 1.91 | 21.27 | 29.70 | 5.46 | 87.44 | 319.20 | 326.68 | Cluster2 |  |
| Gardenia 1 | Gj1A405T61* | UGT85A15 | 0.08 | 66.36 | 137.51 | 130.03 | 109.84 | 229.86 | 285.42 | Cluster6 |  |
| Gardenia 9 | Gj9P206T30* | UGT87A5 (UGT75) | 12.28 | 14.00 | 5.85 | 0.76 | 31.90 | 210.60 | 230.36 | Cluster31 | Y |
| Gardenia 8 | Gj8A383T68 | UGT85A27 | 0.17 | 0.19 | 81.74 | 1.42 | 5.83 | 25.14 | 162.52 | Cluster27 |  |
| Gardenia 4 | Gj4A443T28* | UGT87A7 (UGT67) | 0.53 | 0.13 | 8.01 | 0.00 | 4.38 | 160.98 | 155.42 |  | Y |
| Gardenia 9 | Gj9P883T16 | UGT84A1 | 316.74 | 210.43 | 293.85 | 245.40 | 168.81 | 196.79 | 130.90 | Cluster40 |  |
| Gardenia 8 | Gj8X257T8 | UGT71E8 | 40.95 | 65.29 | 71.38 | 38.78 | 56.69 | 82.82 | 129.76 |  |  |
| Gardenia 11 | Gj11A263T75 | UGT74F6 | 124.19 | 32.52 | 45.57 | 51.38 | 87.74 | 65.95 | 126.11 | Cluster9 |  |
| Gardenia 9 | Gj9X1088T40 | UGT88B1 | 8.72 | 4.19 | 22.14 | 0.46 | 25.56 | 225.86 | 102.06 |  |  |
| Gardenia 9 | Gj9P1027T12 | UGT94E5 | 51.36 | 80.31 | 318.79 | 357.88 | 68.30 | 87.14 | 94.41 | Cluster28 |  |
| Gardenia 4 | Gj4P54T2 | UGT73C14 | 7.97 | 19.75 | 10.90 | 5.71 | 27.60 | 47.84 | 90.52 | Cluster18 |  |
| Gardenia 8 | Gj8P8T11 | UGT74F9 | 87.51 | 29.35 | 68.30 | 144.64 | 51.45 | 97.00 | 81.82 |  |  |
| Gardenia 9 | Gj9P255T12* | UGT94E8 (UGTS24) | 6.90 | 0.44 | 0.14 | 9.43 | 2.02 | 59.12 | 70.06 | Cluster33 | Y |
| Gardenia 7 | Gj7P99T6 | UGT71E7 | 19.60 | 44.47 | 68.98 | 9.55 | 53.38 | 43.17 | 68.56 |  |  |
| Gardenia 9 | Gj9P189T14 | UGT80A2 | 97.97 | 50.24 | 94.45 | 59.56 | 67.29 | 61.59 | 65.01 |  |  |
| Gardenia 4 | Gj4P54T0 | UGT73C13 | 2.45 | 12.34 | 8.14 | 85.55 | 8.53 | 29.87 | 64.93 | Cluster18 |  |
| Gardenia 9 | Gj9A198T89 | UGT74F11 | 20.86 | 1.64 | 15.19 | 5.78 | 14.28 | 48.70 | 59.29 | Cluster30 |  |
| Gardenia 10 | Gj10P134T3* | UGT85A18 (UGTS47) | 1.09 | 2.16 | 4.26 | 5.96 | 8.22 | 45.47 | 49.05 |  |  |
| Gardenia 3 | Gj3P297T15 | UGT94E6 | 11.31 | 81.16 | 50.88 | 16.54 | 51.92 | 46.57 | 40.35 | Cluster13 |  |
| Gardenia 5 | Gj5P306T12 | UGT76E2 | 2.80 | 0.14 | 0.28 | 0.26 | 1.00 | 3.38 | 37.43 | Cluster19 |  |
| Gardenia 1 | Gj1A405T60 | UGT85A22 | 0.04 | 0.03 | 0.11 | 0.00 | 0.38 | 15.82 | 36.42 | Cluster6 |  |
| Gardenia 9 | Gj9P739T9 | UGT75B17 | 2.10 | 9.83 | 4.00 | 1.83 | 4.76 | 20.48 | 34.85 | Cluster38 |  |
| Gardenia 9 | Gj9A900T111 | UGT85A11 | 748.02 | 0.46 | 31.31 | 66.89 | 75.71 | 57.01 | 28.94 | Cluster41 |  |
| Gardenia 4 | Gj4P53T12 | UGT73C7 | 9.71 | 0.25 | 4.94 | 2.14 | 10.55 | 20.88 | 28.92 | Cluster18 |  |
| Gardenia 2 | Gj2X387T58 | UGT79B8 | 17.67 | 13.59 | 5.52 | 17.05 | 9.92 | 32.43 | 27.79 | Cluster12 |  |
| Gardenia 9 | Gj9A1051T98 | UGT85A14 | 5.62 | 0.51 | 4.34 | 1450.78 | 23.73 | 8.62 | 26.64 | Cluster29 |  |
| Gardenia 8 | Gj8A383T63 | UGT85A19 | 9.00 | 26.71 | 25.56 | 20.64 | 17.78 | 23.94 | 25.78 | Cluster27 |  |
| Gardenia 11 | Gj11P363T9 | UGT71A2 | 0.22 | 0.06 | 0.07 | 1.50 | 0.45 | 23.58 | 25.12 |  |  |
| Gardenia 7 | Gj7P357T3 | UGT71A10 | 1.89 | 0.93 | 0.38 | 74.51 | 1.36 | 13.08 | 24.08 | Cluster25 |  |
| Gardenia 11 | Gj11P363T8 | UGT71A1 | 4.58 | 2.73 | 1.14 | 13.33 | 7.79 | 13.25 | 23.48 |  |  |
| Gardenia 9 | Gj9X992T23 | UGT92A13 | 18.75 | 25.82 | 23.15 | 23.04 | 53.62 | 15.11 | 22.79 | Cluster43 |  |
| Gardenia 10 | Gj10X263T40 | UGT79B19 | 15.38 | 10.69 | 15.04 | 21.53 | 16.99 | 17.57 | 22.59 | Cluster3 |  |
| Gardenia 5 | Gj5P5T0 | UGT71E3 | 0.56 | 13.55 | 23.82 | 0.09 | 13.90 | 12.97 | 20.40 | Cluster21 |  |
| Gardenia 9 | Gj9P739T8 | UGT75B10 | 4.56 | 27.09 | 3.95 | 0.35 | 13.50 | 5.44 | 19.47 | Cluster38 |  |
| Gardenia 10 | Gj10A251T57* | UGT85A26 (UGT89) | 11.36 | 0.47 | 0.14 | 0.08 | 0.27 | 25.53 | 19.44 | Cluster2 |  |
| Gardenia 9 | Gj9A1027T106 | UGT94E11 | 4.10 | 2.94 | 7.11 | 9.76 | 8.03 | 48.86 | 18.25 | Cluster28 |  |
| Gardenia 9 | Gj9X316T52 | UGT71E9 | 0.63 | 0.37 | 3.14 | 0.46 | 1.58 | 51.95 | 17.99 |  |  |
| Gardenia 10 | Gj10A263T100 | UGT79B3 | 1.20 | 0.90 | 3.89 | 15.22 | 5.18 | 7.23 | 17.55 | Cluster3 |  |
| Gardenia 9 | Gj9P739T3 | UGT75B8 | 16.73 | 1.20 | 2.72 | 71.46 | 4.45 | 6.20 | 17.09 | Cluster38 |  |
| Gardenia 4 | Gj4X137T73 | UGT71K6 | 15.68 | 63.44 | 11.01 | 231.20 | 16.78 | 10.13 | 16.78 | Cluster15 |  |
| Gardenia 11 | Gj11A263T74 | UGT74F12 | 32.06 | 1.56 | 1.37 | 96.40 | 10.39 | 18.59 | 16.51 | Cluster9 |  |
| Gardenia 4 | Gj4X210T5 | UGT71K8 | 0.00 | 0.25 | 22.24 | 18.12 | 10.26 | 13.97 | 16.05 |  |  |
| Gardenia 9 | Gj9A206T101 | UGT87A4 | 10.54 | 1.55 | 2.92 | 4.99 | 8.45 | 10.64 | 15.79 | Cluster31 |  |
| Gardenia 9 | Gj9P810T16 | UGT89B3 | 10.70 | 32.00 | 9.36 | 85.07 | 28.38 | 13.40 | 15.16 | Cluster39 |  |
| Gardenia 7 | Gj7P356T8 | UGT71A9 | 2.95 | 2.33 | 1.07 | 64.48 | 1.88 | 6.22 | 14.18 | Cluster25 |  |
| Gardenia 9 | Gj9A900T120 | UGT85A13 | 2.17 | 2.13 | 3.16 | 8.94 | 5.44 | 10.79 | 13.92 | Cluster41 |  |
| Gardenia 10 | Gj10A250T66 | UGT85A1 | 10.94 | 5.10 | 9.39 | 12.87 | 30.92 | 7.19 | 13.11 | Cluster2 |  |
| Gardenia 9 | Gj9A605T72 | UGT92A1 | 469.93 | 235.09 | 385.87 | 31.01 | 272.37 | 10.37 | 13.07 | Cluster35 |  |
| Gardenia 5 | Gj5P9T0 | UGT71E5 | 0.18 | 13.08 | 13.66 | 0.25 | 8.13 | 10.98 | 12.13 | Cluster21 |  |
| Gardenia 4 | Gj4P81T0 | UGT71K3 | 26.15 | 2.64 | 124.39 | 27.36 | 15.44 | 17.97 | 11.91 |  |  |
| Gardenia 10 | Gj10P261T21 | UGT79B16 | 40.12 | 28.26 | 6.37 | 0.26 | 3.68 | 30.29 | 11.83 | Cluster3 |  |
| Gardenia 4 | Gj4P51T14 | UGT73C3 | 188.71 | 82.36 | 574.06 | 10.03 | 144.59 | 10.52 | 10.93 | Cluster17 |  |
| Gardenia 9 | Gj9X376T9 | UGT92A4 | 3.59 | 7.32 | 11.66 | 7.16 | 5.06 | 0.91 | 10.92 |  |  |
| Gardenia 2 | Gj2X392T39 | UGT72B3 | 51.54 | 72.19 | 55.83 | 24.70 | 20.40 | 17.77 | 10.71 |  |  |
| Gardenia 4 | Gj4P499T14 | UGT78D16 | 0.86 | 11.79 | 51.54 | 7.14 | 17.30 | 14.26 | 10.69 | Cluster16 |  |
| Gardenia 7 | Gj7P356T7 | UGT71A8 | 35.81 | 4.20 | 15.80 | 2.02 | 9.35 | 11.55 | 10.00 | Cluster25 |  |
| Gardenia 9 | Gj9X193T49 | UGT79B5 | 29.02 | 42.01 | 14.34 | 1.25 | 17.10 | 18.40 | 9.99 | Cluster30 |  |
| Gardenia 8 | Gj8A14T55 | UGT81B1 | 27.03 | 15.95 | 20.77 | 13.66 | 18.20 | 11.81 | 9.77 |  |  |
| Gardenia 9 | Gj9P92T8 | UGT92A6 | 19.09 | 10.90 | 6.99 | 0.08 | 9.08 | 10.21 | 9.71 | Cluster42 |  |
| Gardenia 9 | Gj9X254T42 | UGT94E17 | 2.35 | 0.00 | 0.12 | 2.74 | 10.90 | 3.78 | 9.51 | Cluster33 |  |
| Gardenia 3 | Gj3P26T4 | UGT94E18 | 7.14 | 16.81 | 0.45 | 2.38 | 4.02 | 3.98 | 9.49 |  |  |
| Gardenia 9 | Gj9P739T4* | UGT75L6 | 9.55 | 62.19 | 176.77 | 87.19 | 19.77 | 10.25 | 9.37 | Cluster38 |  |
| Gardenia 4 | Gj4P52T0 | UGT73C5 | 5.14 | 3.70 | 38.40 | 17.74 | 21.00 | 36.57 | 8.37 | Cluster17 |  |
| Gardenia 9 | Gj9X722T84 | UGT92A16 | 1.07 | 6.02 | 8.37 | 7.02 | 7.82 | 5.55 | 8.25 |  |  |
| Gardenia 10 | Gj10P261T20 | UGT79B15 | 13.92 | 10.03 | 4.80 | 0.59 | 4.66 | 21.95 | 8.19 | Cluster3 |  |
| Gardenia 8 | Gj8A384T81 | UGT85A29 | 3.74 | 13.37 | 3.67 | 13.96 | 2.98 | 5.74 | 7.98 | Cluster27 |  |
| Gardenia 9 | Gj9X696T96 | UGT90A5 | 2.63 | 86.08 | 0.69 | 0.10 | 26.79 | 8.26 | 7.77 |  |  |
| Gardenia 9 | Gj9X977T39 | UGT72E1 | 12.65 | 0.85 | 2.77 | 1.92 | 14.73 | 6.28 | 7.69 |  |  |
| Gardenia 11 | Gj11P280T20 | UGT78D7 | 15.54 | 55.81 | 111.54 | 0.40 | 36.86 | 4.10 | 7.65 | Cluster11 |  |
| Gardenia 7 | Gj7P103T3 | UGT71E6 | 2.03 | 1.93 | 8.00 | 21.19 | 7.95 | 2.22 | 7.44 |  |  |
| Gardenia 4 | Gj4P53T17 | UGT73C9 | 0.72 | 1.31 | 19.52 | 2.63 | 8.64 | 0.68 | 7.34 | Cluster18 |  |
| Gardenia 5 | Gj5P5T2 | UGT71E4 | 26.39 | 17.47 | 8.96 | 6.16 | 18.97 | 2.13 | 7.33 | Cluster21 |  |
| Gardenia 9 | Gj9A883T83 | UGT84A3 | 3.43 | 1.61 | 3.12 | 60.46 | 3.86 | 15.02 | 6.40 | Cluster40 |  |
| Gardenia 7 | Gj7P356T0 | UGT71A5 | 12.61 | 15.89 | 130.82 | 2.60 | 47.84 | 7.07 | 6.38 | Cluster25 |  |
| Gardenia 7 | Gj7P357T5 | UGT71A12 | 1.08 | 0.44 | 2.15 | 7.07 | 2.55 | 4.18 | 6.38 | Cluster25 |  |
| Gardenia 4 | Gj4P52T5 | UGT73C6 | 1.51 | 1.81 | 20.91 | 8.17 | 6.81 | 4.63 | 6.27 | Cluster17 |  |
| Gardenia 9 | Gj9P738T3 | UGT75B5 | 7.61 | 3.09 | 0.64 | 0.09 | 2.25 | 1.74 | 5.84 | Cluster38 |  |
| Gardenia 1 | Gj1P494T5 | UGT71K2 | 6.85 | 3.62 | 7.05 | 10.28 | 6.84 | 7.35 | 5.69 | Cluster7 |  |
| Gardenia 9 | Gj9E1027T2 | UGT94E12 | 0.00 | 0.00 | 0.05 | 0.00 | 0.08 | 12.23 | 5.18 | Cluster28 |  |
| Gardenia 5 | Gj5X161T16 | UGT92A15 | 0.82 | 11.46 | 1.14 | 7.89 | 3.68 | 3.67 | 4.61 |  |  |
| Gardenia 8 | Gj8P363T6 | UGT79B20 | 1.44 | 0.00 | 0.22 | 0.08 | 0.08 | 1.63 | 4.13 | Cluster26 |  |
| Gardenia 9 | Gj9P881T18 | UGT92A9 | 23.24 | 7.72 | 3.76 | 17.02 | 16.12 | 1.47 | 4.01 | Cluster40 |  |
| Gardenia 9 | Gj9P738T2 | UGT75B4 | 6.44 | 1.89 | 0.12 | 0.69 | 1.90 | 0.13 | 3.79 | Cluster38 |  |
| Gardenia 9 | Gj9A729T103 | UGT76E7 | 7.33 | 1.59 | 2.27 | 0.10 | 4.23 | 3.88 | 3.70 | Cluster37 |  |
| Gardenia 4 | Gj4P54T3 | UGT73C15 | 26.39 | 1.92 | 5.79 | 0.80 | 12.33 | 4.27 | 3.67 | Cluster18 |  |
| Gardenia 8 | Gj8A384T80 | UGT85A28 | 0.00 | 0.06 | 1.91 | 0.19 | 0.91 | 3.59 | 3.65 | Cluster27 |  |
| Gardenia 9 | Gj9X895T38 | UGT79B21 | 4.80 | 8.85 | 18.67 | 4.69 | 5.71 | 4.36 | 3.64 | Cluster40 |  |
| Gardenia 9 | Gj9P900T17 | UGT85A12 | 0.69 | 0.09 | 2.47 | 0.34 | 3.27 | 4.03 | 3.57 | Cluster41 |  |
| Gardenia 9 | Gj9X686T42 | UGT708A1 | 5.81 | 3.40 | 8.81 | 29.08 | 4.17 | 4.33 | 3.21 | Cluster36 |  |
| Gardenia 10 | Gj10X263T19 | UGT79B12 | 1.02 | 0.56 | 3.21 | 1.00 | 1.89 | 5.04 | 3.20 | Cluster3 |  |
| Gardenia 4 | Gj4A499T86 | UGT78D15 | 0.25 | 14.57 | 12.64 | 0.79 | 9.62 | 3.81 | 3.17 | Cluster16 |  |
| Gardenia 9 | Gj9A1051T97 | UGT85A31 | 0.88 | 2.91 | 6.09 | 36.02 | 3.16 | 2.05 | 3.12 | Cluster29 |  |
| Gardenia 9 | Gj9X810T27 | UGT89B2 | 12.31 | 1.22 | 1.37 | 93.22 | 7.58 | 2.27 | 3.10 | Cluster39 |  |
| Gardenia 4 | Gj4P53T16 | UGT73C8 | 1.49 | 4.50 | 2.07 | 9.34 | 2.94 | 4.74 | 3.03 | Cluster18 |  |
| Gardenia 9 | Gj9P736T19 | UGT75B11 | 30.19 | 11.69 | 1.42 | 0.61 | 3.34 | 0.07 | 3.01 | Cluster38 |  |
| Gardenia 4 | Gj4A52T70 | UGT73C1 | 0.00 | 1.29 | 0.76 | 0.87 | 0.36 | 1.52 | 3.01 | Cluster17 |  |
| Gardenia 1 | Gj1P494T3 | UGT71K1 | 2.27 | 1.79 | 8.66 | 3.57 | 5.28 | 4.53 | 2.86 | Cluster7 |  |
| Gardenia 3 | Gj3A33T42 | UGT74E1 | 0.00 | 0.00 | 0.14 | 0.97 | 0.38 | 2.51 | 2.77 | Cluster14 |  |
| Gardenia 11 | Gj11X419T45 | UGT74F5 | 138.61 | 29.54 | 327.98 | 21.48 | 125.55 | 3.32 | 2.71 |  |  |
| Gardenia 4 | Gj4P53T2 | UGT73C10 | 2.60 | 0.00 | 0.00 | 0.83 | 0.19 | 0.99 | 2.57 | Cluster17 |  |
| Gardenia 1 | Gj1A507T92 | UGT80A1 | 12.07 | 2.39 | 3.98 | 2.35 | 4.57 | 2.18 | 2.40 | Cluster7 |  |
| Gardenia 10 | Gj10P303T18 | UGT72D1 | 0.41 | 1.49 | 14.25 | 0.74 | 6.55 | 1.66 | 2.23 | Cluster5 |  |
| Gardenia 4 | Gj4P55T5 | UGT73C18 | 0.28 | 0.00 | 0.00 | 0.15 | 0.00 | 0.00 | 2.23 | Cluster18 |  |
| Gardenia 4 | Gj4X137T45 | UGT71K5 | 1.39 | 2.16 | 1.92 | 1.97 | 4.83 | 0.24 | 2.15 | Cluster15 |  |
| Gardenia 5 | Gj5P47T7 | UGT74F10 | 0.47 | 0.06 | 0.05 | 3.14 | 0.03 | 0.35 | 2.14 | Cluster20 |  |
| Gardenia 10 | Gj10P262T3 | UGT79B10 | 4.01 | 0.14 | 0.00 | 0.00 | 0.06 | 0.27 | 1.97 | Cluster3 |  |
| Gardenia 4 | Gj4P55T4 | UGT73C17 | 0.00 | 0.00 | 0.00 | 0.16 | 0.03 | 4.94 | 1.96 | Cluster18 |  |
| Gardenia 5 | Gj5A5T42 | UGT71E2 | 1.64 | 1.93 | 1.86 | 0.28 | 1.11 | 0.00 | 1.78 | Cluster21 |  |
| Gardenia 3 | Gj3A33T43 | UGT74E2 | 4.68 | 22.02 | 8.30 | 0.06 | 14.95 | 1.35 | 1.67 | Cluster14 |  |
| Gardenia 4 | Gj4A446T29 | UGT87A8 | 38.41 | 0.17 | 0.04 | 0.00 | 0.74 | 20.43 | 1.67 |  |  |
| Gardenia 10 | Gj10A262T82 | UGT79B1 | 1.97 | 9.02 | 19.36 | 2.70 | 13.88 | 0.60 | 1.57 | Cluster3 |  |
| Gardenia 4 | Gj4P56T4 | UGT73C19 | 1.61 | 0.00 | 0.34 | 6.86 | 0.03 | 1.41 | 1.53 | Cluster18 |  |
| Gardenia 9 | Gj9X606T56 | UGT92A2 | 1.93 | 2.19 | 1.77 | 0.71 | 2.10 | 1.38 | 1.48 | Cluster35 |  |
| Gardenia 8 | Gj8A285T32 | UGT85A4 | 1.35 | 1.08 | 0.28 | 2.29 | 0.81 | 1.23 | 1.48 |  |  |
| Gardenia 10 | Gj10A262T83 | UGT79B9 | 13.05 | 9.53 | 2.58 | 8.14 | 7.94 | 3.04 | 1.41 | Cluster3 |  |
| Gardenia 9 | Gj9X540T43 | UGT72B1 | 0.00 | 1.61 | 1.87 | 0.21 | 0.40 | 0.00 | 1.40 | Cluster34 |  |
| Gardenia 9 | Gj9P92T7 | UGT92A5 | 1.42 | 2.76 | 114.38 | 0.08 | 3.62 | 1.57 | 1.36 | Cluster42 |  |
| Gardenia 11 | Gj11X380T17 | UGT94E21 | 0.35 | 2.67 | 32.92 | 0.40 | 15.74 | 2.87 | 1.36 |  |  |
| Gardenia 10 | Gj10P261T22 | UGT79B11 | 12.97 | 0.00 | 0.00 | 0.00 | 0.07 | 1.01 | 1.35 | Cluster3 |  |
| Gardenia 10 | Gj10A262T84 | UGT79B14 | 24.53 | 4.22 | 3.18 | 3.16 | 5.23 | 2.22 | 1.34 | Cluster3 |  |
| Gardenia 9 | Gj9P738T5 | UGT75B7 | 0.00 | 0.09 | 0.00 | 28.92 | 0.31 | 0.09 | 1.23 | Cluster38 |  |
| Gardenia 6 | Gj6A216T43 | UGT74F7 | 6.79 | 3.69 | 1.33 | 10.42 | 8.71 | 2.18 | 1.20 |  |  |
| Gardenia 11 | Gj11P280T10 | UGT78D6 | 2.43 | 9.49 | 0.66 | 0.15 | 3.12 | 0.34 | 1.13 | Cluster11 |  |
| Gardenia 11 | Gj11X395T26 | UGT89B1 | 13.71 | 18.79 | 27.93 | 1.11 | 9.42 | 4.57 | 1.13 |  |  |
| Gardenia 9 | Gj9P881T15 | UGT92A8 | 24.86 | 9.88 | 4.84 | 1.00 | 12.10 | 0.64 | 1.12 | Cluster40 |  |
| Gardenia 10 | Gj10P227T10 | UGT85A25 | 4.14 | 0.26 | 0.03 | 0.25 | 0.07 | 3.99 | 1.00 | Cluster1 |  |
| Gardenia 6 | Gj6A316T89 | UGT87A2 | 0.08 | 0.26 | 6.39 | 4.29 | 1.41 | 0.26 | 0.92 | Cluster23 |  |
| Gardenia 4 | Gj4X138T36 | UGT71K7 | 0.00 | 0.00 | 0.00 | 0.00 | 0.00 | 0.33 | 0.90 | Cluster15 |  |
| Gardenia 9 | Gj9X992T30 | UGT92A14 | 0.22 | 3.10 | 75.12 | 0.35 | 38.98 | 0.40 | 0.89 | Cluster43 |  |
| Gardenia 4 | Gj4P51T12 | UGT73C2 | 0.46 | 0.97 | 0.62 | 18.11 | 0.61 | 1.76 | 0.87 | Cluster17 |  |
| Gardenia 9 | Gj9P739T6 | UGT75B15 | 7.57 | 1.13 | 0.00 | 0.52 | 0.47 | 0.23 | 0.82 | Cluster38 |  |
| Gardenia 6 | Gj6P93T1 | UGT78D9 | 0.75 | 0.06 | 0.37 | 3.09 | 0.80 | 0.27 | 0.80 | Cluster24 |  |
| Gardenia 9 | Gj9A736T102 | UGT75B2 | 0.00 | 0.31 | 0.00 | 19.26 | 0.14 | 0.00 | 0.79 | Cluster38 |  |
| Gardenia 11 | Gj11A234T87 | UGT76E3 | 0.00 | 0.06 | 0.18 | 0.00 | 0.30 | 0.45 | 0.75 | Cluster8 |  |
| Gardenia 1 | Gj1A406T23 | UGT76B1 | 0.00 | 0.85 | 0.16 | 218.76 | 1.24 | 0.41 | 0.74 | Cluster6 |  |
| Gardenia 11 | Gj11A261T50 | UGT74F13 | 106.70 | 65.23 | 68.74 | 12.07 | 33.19 | 2.38 | 0.70 | Cluster9 |  |
| Gardenia 9 | Gj9P739T5 | UGT75B16 | 9.85 | 2.07 | 0.28 | 0.00 | 0.88 | 0.31 | 0.68 | Cluster38 |  |
| Gardenia 6 | Gj6X244T13 | UGT90A2 | 0.44 | 0.00 | 0.00 | 0.00 | 0.25 | 0.22 | 0.68 | Cluster22 |  |
| Gardenia 9 | Gj9X738T77 | UGT75B14 | 4.83 | 0.46 | 0.15 | 0.00 | 0.93 | 0.22 | 0.68 | Cluster38 |  |
| Gardenia 7 | Gj7A2T48 | UGT78D11 | 0.76 | 0.21 | 2.41 | 2.32 | 2.16 | 0.76 | 0.63 |  |  |
| Gardenia 7 | Gj7P356T4 | UGT71A6 | 1.83 | 0.54 | 21.94 | 0.00 | 4.57 | 0.39 | 0.60 | Cluster25 |  |
| Gardenia 9 | Gj9P739T7 | UGT75B9 | 0.12 | 0.27 | 0.00 | 0.00 | 1.91 | 0.13 | 0.56 | Cluster38 |  |
| Gardenia 9 | Gj9P736T18 | UGT75B13 | 1.47 | 37.33 | 2.17 | 0.00 | 7.47 | 0.00 | 0.56 | Cluster38 |  |
| Gardenia 9 | Gj9A1027T105 | UGT94E10 | 0.00 | 0.00 | 0.35 | 0.00 | 0.93 | 0.44 | 0.55 | Cluster28 |  |
| Gardenia 8 | Gj8P39T4 | UGT94E16 | 0.00 | 0.03 | 0.29 | 0.00 | 0.00 | 0.33 | 0.55 |  |  |
| Gardenia 9 | Gj9P738T4 | UGT75B6 | 0.61 | 1.46 | 0.29 | 3.99 | 0.12 | 0.00 | 0.50 | Cluster38 |  |
| Gardenia 9 | Gj9P736T12 | UGT75B3 | 0.00 | 0.71 | 2.01 | 94.01 | 1.16 | 0.07 | 0.50 | Cluster38 |  |
| Gardenia 9 | Gj9P1001T7 | UGT90A4 | 8.35 | 12.02 | 27.43 | 0.39 | 12.66 | 0.72 | 0.50 |  |  |
| Gardenia 4 | Gj4X454T15 | UGT94E7 | 1.31 | 8.20 | 4.57 | 1.86 | 13.30 | 1.18 | 0.48 |  |  |
| Gardenia 7 | Gj7X286T10 | UGT88F2 | 1.53 | 0.78 | 0.10 | 1.34 | 0.30 | 0.07 | 0.46 |  |  |
| Gardenia 9 | Gj9P684T17 | UGT78D19 | 2.19 | 1.25 | 1.39 | 9.68 | 1.13 | 0.95 | 0.40 | Cluster36 |  |
| Gardenia 10 | Gj10P262T6 | UGT79B13 | 0.47 | 0.00 | 0.00 | 0.35 | 0.03 | 0.13 | 0.39 | Cluster3 |  |
| Gardenia 7 | Gj7P356T6 | UGT71A7 | 0.05 | 0.00 | 0.00 | 0.00 | 0.04 | 0.00 | 0.38 | Cluster25 |  |
| Gardenia 1 | Gj1A404T47 | UGT85A16 | 0.68 | 0.57 | 0.31 | 0.16 | 1.85 | 0.00 | 0.37 | Cluster6 |  |
| Gardenia 8 | Gj8A384T82 | UGT85A3 | 0.55 | 0.00 | 0.00 | 0.00 | 0.05 | 1.38 | 0.37 | Cluster27 |  |
| Gardenia 4 | Gj4P51T15 | UGT73C4 | 12.54 | 0.22 | 1.18 | 1.02 | 1.34 | 0.99 | 0.35 | Cluster17 |  |
| Gardenia 5 | Gj5P46T6 | UGT74F2 | 0.04 | 0.00 | 0.00 | 0.00 | 0.48 | 0.14 | 0.35 | Cluster20 |  |
| Gardenia 10 | Gj10P263T12 | UGT79B18 | 1.89 | 1.13 | 3.17 | 0.17 | 0.72 | 0.74 | 0.34 | Cluster3 |  |
| Gardenia 9 | Gj9A684T98 | UGT78D18 | 0.09 | 1.17 | 1.15 | 0.34 | 1.32 | 0.33 | 0.33 | Cluster36 |  |
| Gardenia 10 | Gj10P303T19 | UGT72D2 | 19.94 | 2.91 | 0.99 | 0.00 | 1.72 | 0.00 | 0.33 | Cluster5 |  |
| Gardenia 4 | Gj4P53T4 | UGT73C11 | 11.00 | 0.17 | 1.00 | 0.08 | 1.85 | 0.58 | 0.32 | Cluster17 |  |
| Gardenia 8 | Gj8P39T2 | UGT73C21 | 0.00 | 0.00 | 0.00 | 0.00 | 0.00 | 0.21 | 0.29 |  |  |
| Gardenia 9 | Gj9A216T135 | UGT76C4 | 1.28 | 1.49 | 2.35 | 0.09 | 0.99 | 0.34 | 0.28 |  |  |
|  | Gju80P0T1 | UGT73C22 | 0.00 | 0.00 | 0.00 | 0.00 | 0.00 | 0.11 | 0.28 |  |  |
| Gardenia 2 | Gj2A98T64 | UGT78D14 | 0.00 | 0.03 | 3.11 | 0.00 | 0.29 | 0.75 | 0.21 |  |  |
| Gardenia 9 | Gj9P1051T15 | UGT85A20 | 8.25 | 0.00 | 0.41 | 0.60 | 2.56 | 0.05 | 0.17 | Cluster29 |  |
| Gardenia 2 | Gj2X388T39 | UGT79B7 | 0.14 | 0.88 | 0.44 | 3.52 | 0.68 | 0.48 | 0.17 | Cluster12 |  |
| Gardenia 11 | Gj11A279T83 | UGT78D3 | 0.00 | 5.42 | 1.31 | 0.09 | 2.23 | 0.54 | 0.17 | Cluster10 |  |
| Gardenia 10 | Gj10P262T2 | UGT79B17 | 0.04 | 0.03 | 0.00 | 0.00 | 0.00 | 0.00 | 0.13 | Cluster3 |  |
| Gardenia 5 | Gj5A42T28 | UGT74F4 | 0.00 | 0.00 | 0.00 | 0.00 | 0.00 | 0.00 | 0.12 | Cluster20 |  |
| Gardenia 11 | Gj11P263T6 | UGT74F3 | 3.06 | 0.11 | 6.61 | 2.35 | 4.90 | 0.00 | 0.12 | Cluster9 |  |
| Gardenia 11 | Gj11X236T18 | UGT76E1 | 0.29 | 0.03 | 0.04 | 0.00 | 1.13 | 0.21 | 0.12 | Cluster8 |  |
| Gardenia 8 | Gj8X318T47 | UGT88F1 | 1.35 | 0.45 | 0.09 | 1.07 | 0.37 | 0.07 | 0.11 |  |  |
| Gardenia 11 | Gj11P280T7 | UGT78D5 | 0.00 | 1.93 | 1.31 | 0.00 | 0.73 | 1.80 | 0.11 | Cluster11 |  |
| Gardenia 5 | Gj5P52T12 | UGT92A12 | 5.72 | 0.15 | 0.53 | 0.00 | 0.28 | 0.13 | 0.11 |  |  |
| Gardenia 8 | Gj8P363T5 | UGT79B2 | 0.64 | 0.03 | 0.15 | 0.00 | 0.25 | 0.06 | 0.11 | Cluster26 |  |
| Gardenia 5 | Gj5A46T63 | UGT74F1 | 0.00 | 0.00 | 2.20 | 0.00 | 1.67 | 0.28 | 0.06 | Cluster20 |  |
| Gardenia 4 | Gj4P53T6 | UGT73C12 | 1.29 | 0.03 | 9.77 | 0.27 | 1.58 | 0.00 | 0.06 | Cluster17 |  |
| Gardenia 10 | Gj10P221T21 | UGT94E1 | 0.16 | 0.00 | 0.00 | 0.09 | 0.00 | 0.34 | 0.06 | Cluster4 |  |
| Gardenia 10 | Gj10A227T81 | UGT85A23 | 0.00 | 0.00 | 0.05 | 0.00 | 0.10 | 0.14 | 0.06 | Cluster1 |  |
| Gardenia 9 | Gj9P92T9 | UGT92A3 | 25.34 | 1.67 | 0.11 | 0.34 | 0.06 | 0.00 | 0.05 | Cluster42 |  |
| Gardenia 6 | Gj6A243T40 | UGT90A1 | 0.00 | 0.00 | 0.00 | 0.00 | 0.00 | 0.00 | 0.05 | Cluster22 |  |
| Gardenia 10 | Gj10A250T63 | UGT85A6 | 1.37 | 0.00 | 0.04 | 0.00 | 0.00 | 0.86 | 0.05 | Cluster2 |  |
| Gardenia 9 | Gj9A900T118 | UGT85A5 | 0.29 | 0.44 | 1.14 | 4.62 | 0.58 | 0.06 | 0.05 | Cluster41 |  |
| Gardenia 9 | Gj9A900T119 | UGT85A32 | 0.29 | 0.44 | 1.14 | 4.62 | 0.58 | 0.06 | 0.05 | Cluster41 |  |
| Gardenia 10 | Gj10A251T59 | UGT85A7 | 0.00 | 0.00 | 0.11 | 0.00 | 0.00 | 0.00 | 0.00 | Cluster2 |  |
| Gardenia 10 | Gj10P221T20 | UGT94E4 | 0.08 | 0.10 | 0.00 | 0.26 | 1.62 | 0.00 | 0.00 | Cluster4 |  |
| Gardenia 10 | Gj10P250T8 | UGT85A9 | 0.00 | 0.00 | 0.00 | 0.00 | 0.00 | 0.00 | 0.00 | Cluster2 |  |
| Gardenia 10 | Gj10X205T21 | UGT92A7 | 0.00 | 0.00 | 0.00 | 0.00 | 0.00 | 0.00 | 0.00 |  |  |
| Gardenia 10 | Gj10X227T60 | UGT84A2 | 0.00 | 0.00 | 0.00 | 0.00 | 0.00 | 0.00 | 0.00 | Cluster1 |  |
| Gardenia 11 | Gj11A247T47 | UGT85A10 | 0.00 | 0.00 | 0.00 | 1.40 | 0.00 | 0.00 | 0.00 |  |  |
| Gardenia 11 | Gj11A290T115 | UGT78D2 | 0.00 | 0.00 | 0.00 | 0.00 | 0.00 | 0.00 | 0.00 |  |  |
| Gardenia 11 | Gj11P163T0 | UGT85A17 | 0.04 | 0.19 | 0.27 | 0.00 | 0.21 | 0.00 | 0.00 |  |  |
| Gardenia 11 | Gj11P279T11 | UGT78D1 | 0.00 | 1.97 | 1.53 | 0.00 | 0.14 | 0.20 | 0.00 | Cluster10 |  |
| Gardenia 11 | Gj11P279T18 | UGT78D4 | 1.46 | 11.53 | 6.75 | 0.00 | 2.11 | 0.00 | 0.00 | Cluster10 |  |
| Gardenia 11 | Gj11P280T8 | UGT78D8 | 0.31 | 4.50 | 3.00 | 0.00 | 1.50 | 0.07 | 0.00 | Cluster11 |  |
| Gardenia 11 | Gj11P50T0 | UGT75B1 | 0.00 | 0.00 | 0.00 | 0.00 | 0.00 | 0.00 | 0.00 |  |  |
| Gardenia 11 | Gj11X234T8 | UGT94E2 | 0.00 | 0.00 | 0.00 | 0.00 | 1.31 | 0.00 | 0.00 | Cluster8 |  |
| Gardenia 11 | Gj11X236T42 | UGT94E3 | 0.00 | 0.04 | 0.00 | 0.00 | 2.85 | 0.00 | 0.00 | Cluster8 |  |
| Gardenia 11 | Gj11X281T19 | UGT78D12 | 0.00 | 0.56 | 35.20 | 0.00 | 7.42 | 0.00 | 0.00 | Cluster11 |  |
| Gardenia 11 | Gj11X379T64 | UGT94E19 | 0.00 | 0.00 | 0.00 | 0.00 | 1.75 | 0.00 | 0.00 |  |  |
| Gardenia 11 | Gj11X421T21 | UGT94E14 | 0.00 | 0.00 | 0.00 | 0.00 | 0.00 | 0.00 | 0.00 |  |  |
| Gardenia 1 | Gj1P76T7 | UGT79B6 | 0.00 | 0.00 | 0.00 | 0.00 | 0.03 | 0.00 | 0.00 |  |  |
| Gardenia 2 | Gj2A247T10 | UGT71E1 | 0.00 | 0.00 | 0.00 | 0.00 | 0.00 | 0.00 | 0.00 |  |  |
| Gardenia 3 | Gj3A34T148 | UGT74E3 | 1.92 | 0.35 | 0.00 | 0.00 | 0.04 | 0.08 | 0.00 | Cluster14 |  |
| Gardenia 3 | Gj3A35T105 | UGT74E4 | 0.00 | 0.00 | 0.00 | 3.17 | 0.03 | 0.00 | 0.00 | Cluster14 |  |
| Gardenia 3 | Gj3P297T13 | UGT94E15 | 0.00 | 0.14 | 0.31 | 0.00 | 0.56 | 0.31 | 0.00 | Cluster13 |  |
| Gardenia 4 | Gj4A499T84 | UGT78D13 | 0.00 | 0.00 | 0.00 | 0.00 | 0.00 | 0.00 | 0.00 | Cluster16 |  |
| Gardenia 4 | Gj4P55T1 | UGT73C16 | 0.06 | 0.06 | 0.44 | 0.13 | 0.12 | 0.20 | 0.00 | Cluster18 |  |
| Gardenia 4 | Gj4P56T5 | UGT73C20 | 0.00 | 0.06 | 0.00 | 0.08 | 0.13 | 0.00 | 0.00 | Cluster18 |  |
| Gardenia 4 | Gj4X137T25 | UGT71K4 | 0.15 | 0.00 | 0.00 | 0.00 | 0.00 | 0.00 | 0.00 | Cluster15 |  |
| Gardenia 5 | Gj5A305T50 | UGT85A21 | 0.00 | 0.27 | 0.00 | 0.00 | 0.00 | 0.00 | 0.00 | Cluster19 |  |
| Gardenia 5 | Gj5A306T79 | UGT76E5 | 0.00 | 0.00 | 0.00 | 0.00 | 0.00 | 0.00 | 0.00 | Cluster19 |  |
| Gardenia 5 | Gj5A42T27 | UGT76E6 | 0.00 | 0.00 | 0.00 | 0.00 | 0.00 | 0.00 | 0.00 | Cluster20 |  |
| Gardenia 5 | Gj5P52T3 | UGT92A11 | 0.00 | 0.00 | 0.00 | 0.00 | 0.14 | 0.06 | 0.00 |  |  |
| Gardenia 5 | Gj5P76T6 | UGT72E2 | 0.00 | 0.00 | 0.00 | 0.00 | 0.00 | 0.00 | 0.00 |  |  |
| Gardenia 6 | Gj6A316T90 | UGT87A3 | 0.76 | 0.58 | 1.73 | 5.66 | 1.00 | 0.24 | 0.00 | Cluster23 |  |
| Gardenia 6 | Gj6A92T35 | UGT78D10 | 0.00 | 0.00 | 0.00 | 0.00 | 0.00 | 0.00 | 0.00 | Cluster24 |  |
| Gardenia 6 | Gj6A93T32 | UGT78D17 | 0.00 | 0.00 | 0.00 | 0.00 | 0.00 | 0.00 | 0.00 | Cluster24 |  |
| Gardenia 6 | Gj6X245T9 | UGT90A3 | 0.00 | 0.00 | 0.00 | 0.00 | 0.00 | 0.00 | 0.00 | Cluster22 |  |
| Gardenia 7 | Gj7A355T79 | UGT71A3 | 0.00 | 0.00 | 0.00 | 0.00 | 0.00 | 0.00 | 0.00 | Cluster25 |  |
| Gardenia 7 | Gj7A356T49 | UGT71A4 | 0.00 | 0.00 | 0.00 | 0.00 | 0.00 | 0.00 | 0.00 | Cluster25 |  |
| Gardenia 8 | Gj8P117T1 | UGT94E9 | 5.53 | 10.02 | 0.27 | 0.14 | 0.86 | 0.11 | 0.00 |  |  |
| Gardenia 8 | Gj8X384T15 | UGT85A30 | 0.00 | 0.00 | 0.14 | 0.00 | 0.00 | 0.00 | 0.00 | Cluster27 |  |
| Gardenia 9 | Gj9A242T106 | UGT87A6 | 0.22 | 0.39 | 0.09 | 0.25 | 0.00 | 0.19 | 0.00 | Cluster32 |  |
| Gardenia 9 | Gj9A729T102 | UGT76E4 | 0.00 | 0.00 | 0.00 | 0.09 | 0.00 | 0.00 | 0.00 | Cluster37 |  |
| Gardenia 9 | Gj9A881T92 | UGT92A10 | 0.14 | 0.00 | 0.00 | 0.24 | 0.17 | 0.19 | 0.00 | Cluster40 |  |
| Gardenia 9 | Gj9P1025T22 | UGT85A24 | 0.30 | 0.35 | 0.76 | 0.84 | 0.11 | 0.33 | 0.00 | Cluster28 |  |
| Gardenia 9 | Gj9P242T17 | UGT87A1 | 0.75 | 0.00 | 0.26 | 0.00 | 0.00 | 0.00 | 0.00 | Cluster32 |  |
| Gardenia 9 | Gj9X113T58 | UGT79B4 | 0.00 | 0.00 | 0.00 | 0.13 | 0.00 | 0.00 | 0.00 |  |  |
| Gardenia 9 | Gj9X540T63 | UGT72B2 | 0.69 | 1.15 | 0.06 | 0.00 | 0.23 | 0.08 | 0.00 | Cluster34 |  |
| Gardenia 9 | Gj9X644T42 | UGT94E20 | 0.00 | 0.00 | 0.00 | 0.00 | 0.00 | 0.00 | 0.00 |  |  |
| Gardenia 9 | Gj9X738T36 | UGT75B12 | 0.00 | 0.00 | 0.00 | 0.00 | 0.00 | 0.00 | 0.00 | Cluster38 |  |

*Candidate GjUGTs for crocetin biosynthesis characterized in this study. In brackets are reported the names of the UGTs identified in a previous study (Ji et al., 2017).

Additional file 2: Table S14. NMR data for the intermediate product β-apo-8′-carotenal derived from the cleavage of β-carotene by GjCCD4a.

|  | ^1^H | ^13^C |
| --- | --- | --- |
| 1 | - | 34.4 |
| 2 | 1.47, m, 2H | 39.7 |
| 3 | 1.62, m, 2H | 19.4 |
| 4 | 2.02, m, 2H | 33.3 |
| 5 | - | 129.3 |
| 6 | - | 138.8 |
| 7 | 6.21, d, *J*=16.2, 1H | 126.4 |
| 8 | 6.14, d, *J*=16.2, 1H | 138 |
| 9 | - | 137 |
| 10 | 6.16, d, *J*=11.4, 1H | 133.2 |
| 11 | dd | 122.8 |
| 12 | 6.36, d, *J*=15, 1H | 137.8 |
| 13 | - | 137.1 |
| 14 | 6.27, d, *J*=11.4, 1H | 129.7 |
| 15 | dd | 132.1 |
| 16 | 1.03, s, 6H | 29.1 |
| 17 |  | 29.1 |
| 18 | 1.72, s, 3H | 21.9 |
| 19 | 1.98, s, 3H | 13.1 |
| 20 | 2.00, s, 3H | 13 |
| 8' | 9.46, *J*=10.9, 1H | 194.8 |
| 9' | - | 146.2 |
| 10' | 6.94, *J*=11.4, 1H | 149.6 |
| 11' | dd | 135.3 |
| 12' | d | 136.8 |
| 13' | - | 137.8 |
| 14' | 6.45, d, *J*=11.4, 1H | 127.4 |
| 15' | dd | 130.8 |
| 19' | 1.90, s, 3H | 9.8 |
| 20' | 2.00, s, 3H | 12.8 |

Additional file 2: Table S15. Synteny of *CCD4*, *ALDH2C*, and *UGT94E* genes among *Gardenia*, *Coffea*, and Tomato.

| CCD4 | Gardenia | Coffea | Tomato |
| --- | --- | --- | --- |
|  | Gj9A597T64 | Cc08_g05590 |  |
|  | Gj9P597T12 | Cc08_g05600 |  |
| CCD4 | Gj9A597T69 (GjCCD4a) |  |  |
| CCD4 | Gj9A597T68 (GjCCD4b) |  |  |
| CCD4 | Gj9A597T67 (GjCCD4c) |  | Solyc08g075490.2.1  (SlCCDb) |
| CCD4 | Gj9P597T6 (GjCCD4d) | Cc08_g05610 (CcCCD4) | Solyc08g075480.2.1 (SlCCD4a) |
|  | Gj9P596T14 | Cc08_g05620 | Solyc08g075450.2.1 |
|  | Gj9A596T79 | Cc08_g05640 | Solyc08g075430.2.1 |
|  | Gj9A596T78 | Cc08_g05650 | Solyc08g075420.2.1 |
|  |  |  |  |
| ALDH | Gardenia | Coffea | Tomato |
|  | Gj9P24T18 | Cc06_g09510 |  |
|  | Gj9P24T11 | Cc06_g09530 |  |
|  | Gj9P24T17 | Cc06_g09540 |  |
| ALDH2C | Gj9A24T70 (GjALDH2C3) | Cc06_g09550 | Solyc12g007030.1.1 (SlALDH2C4) |
| ALDH2C | Gj9A24T69 (GjALDH2C2) | Cc06_g09560 |  |
|  | Gj9P24T7 | Cc06_g09580 |  |
|  | Gj9A24T73 | Cc06_g09590 |  |
|  |  |  |  |
| UGT | Gardenia | Coffea | Tomato |
|  | Gj9P1027T25 | Cc02_g09980 | Solyc11g007320.1.1 |
|  | Gj9P1027T17 | Cc02_g09990 | Solyc11g007330.1.1 |
| UGT94E | Gj9A1027T106 (GjUGT94E11) | Cc02_g10010 |  |
| UGT94E | Gj9E1027T2 (GjUGT94E12) |  |  |
| UGT94E | Gj9A1027T105 (GjUGT94E10) | Cc02_g10030 | Solyc11g007350.1.1 |
| UGT94E | Gj9P1027T12 (GjUGT94E5) | Cc02_g10040 | Solyc11g007360.1.1 |
| UGT94E | Gj9P1027T10 (GjUGT94E13) | Cc02_g10050 | Solyc11g007370.1.1 |
|  | Gj9A1027T109 | Cc02_g10070 | Solyc11g007530.1.1 |
|  | Gj9X1026T64 | Cc02_g10080 | Solyc11g007540.1.1 |

| UGT | Gardenia | Coffea | Tomato |
| --- | --- | --- | --- |
|  | Gj9A198T86 | Cc01_g15770 | Solyc09g019970.2.1 |
|  | Gj9A198T85 | Cc01_g15780 | Solyc09g020000.2.1 |
|  | Gj9A198T91 | Cc01_g15790 | Solyc06g082910.2.1 |
|  | Gj9P198T10 | Cc01_g15800 |  |
| UGT74F | Gj9A198T90  (GjUGT74F8) | Cc01_g15810 |  |
| UGT74F | Gj9A198T89  (GjUGT74F11) | Cc01_g15820 |  |
|  | Gj9P198T7 | Cc01_g15830 | Solyc06g082920.2.1 |
|  | Gj9A197T171 | Cc01_g15840 | Solyc06g082930.2.1 |
|  | Gj9A197T170 | Cc01_g15850 |  |
|  | Gj9A197T158 | Cc01_g15860 |  |

Additional file 2: Table S16. Correspondence between candidate genes identified in the present study and unigenes identified in a previous transcriptome study (Ji et al., 2017).

| **Genome** | **Enzyme** | **Transcriptome** |
| --- | --- | --- |
| Gj9A597T69 | GjCCD4a* | GjCCD4a |
| Gj9A24T70 | GjALDH2C3* | ALDH12 |
| Gj11A422T108 | GjALDH2B1 | ALDH14 |
| Gj9A198T90 | UGT74F8* | None |
| GJ61E11T0 | UGT85A15 | None |
| Gj7P357T4 | UGT71A11 | None |
| Gj8A384T85 | UGT85A8 | None |
| Gj10A251T58 | UGT85A2 | None |
| Gj9P1027T10 | UGT94E13* | UGT60 |
| Gj4A443T28 | UGT87A7 | UGT67 |
| Gj10A251T57 | UGT85A26 | UGT89 |
| Gj9P206T30 | UGT87A5 | UGT75 |
| None | UGT71H4 | UGT86 |
| Gj10P134T3 | UGT85A18 | UGTS47 |
| Gj9P255T12 | UGT94E8 | UGTS24 |

*Candidate enzymes involved in crocin biosynthesis

Additional file 2: Table S17. Nomenclature of crocins in *G. jasminoides* and *C. sativus*.

|  | Crocins in *G. jasminoides* | Crocins in *C. sativus* | IUPAC name |
| --- | --- | --- | --- |
| Crocetin di-gentiobiosyl ester | Crocin I | Crocin 4 | bis[(2*S*,3*R*,4*S*,5*S*,6*R*)-3,4,5-trihydroxy-6-[[(2*R*,3*R*,4*S*,5*S*,6*R*)-3,4,5-trihydroxy-6-(hydroxymethyl)oxan-2-yl]oxymethyl]oxan-2-yl] (2*E*,4*E*,6*E*,8*E*,10*Z*,12*E*,14*Z*)-2,6,11,15-tetramethylhexadeca-2,4,6,8,10,12,14-heptaenedioate |
| Crocetin glucosyl-gentiobiosyl ester | Crocin II | Crocin 3 | 1-*O*-[(2*R*,3*R*,4*S*,5*S*,6*R*)-3,4,5-trihydroxy-6-(hydroxymethyl)oxan-2-yl] 16-*O*-[(2*S*,3*R*,4*S*,5*S*,6*R*)-3,4,5-trihydroxy-6-[[(2*R*,3*R*,4*S*,5*S*,6*R*)-3,4,5-trihydroxy-6-(hydroxymethyl)oxan-2-yl]oxymethyl]oxan-2-yl] (2*E*,4*E*,6*E*,8*E*,10*E*,12*E*,14*E*)-2,6,11,15-tetramethylhexadeca-2,4,6,8,10,12,14-heptaenedioate |
| Crocetin di-glucosyl ester | Crocin III | Crocin 2′ | 1-[(2*R*,3*S*,4*R*,5*R*,6*S*)-3,4,5-trihydroxy-6-(hydroxymethyl)tetrahydro-2H-pyran-2-yl] 16-[(2*S*,3*R*,4*S*,5*S*,6*R*)-3,4,5-trihydroxy-6-(hydroxymethyl)tetrahydro-2H-pyran-2-yl] (2*E*,4*E*,6*E*,8*E*,10*E*,12*E*,14*E*)-2,6,11,15-tetramethylhexadeca-2,4,6,8,10,12,14-heptaenedioate |
| Crocetin mono-gentiobiosyl ester | Crocin IV | Crocin 2 | (2*E*,4*E*,6*E*,8*E*,10*E*,12*E*,14*E*)-2,6,11,15-tetramethyl-16-oxo-16-[(2*S*,3*R*,4*S*,5*S*,6*R*)-3,4,5-trihydroxy-6-[[(2*R*,3*R*,4*S*,5*S*,6*R*)-3,4,5-trihydroxy-6-(hydroxymethyl)oxan-2-yl]oxymethyl]oxan-2-yl]oxyhexadeca-2,4,6,8,10,12,14-heptaenoic acid |
| Crocetin mono-glucosyl ester | Crocin V | Crocin 1 | 1-*O*-methyl 16-*O*-[3,4,5-trihydroxy-6-(hydroxymethyl)oxan-2-yl] (2*Z*,4*E*,6*E*,8*E*,10*Z*,12*E*,14*E*)-2,6,11,15-tetramethylhexadeca-2,4,6,8,10,12,14-heptaenedioate |

Additional file 2: Table S18. UPLC-DAD-MS or NMR characteristics of apocarotenoids and carotenoids analyzed in this study.

| Compound | Retention time (min) | λmax (nm) | *m/z* values | MassBank of North America (MoNA) ID |
| --- | --- | --- | --- | --- |
| crocin I | 4.22 | 262.3, 440.3, 463.8 | [M+Na]^+^: 999.3699 | 011440 |
| crocin II | 4.56 | 262.0, 439.6, 463.2 | [M+Na]^+^:837.3136 | 011441 |
| crocin III | 4.96 | 261.7, 438.9, 462.3 | [M+Na]^+^: 675.2620 | 011443 |
| crocin IV | 5.75 | 259.6, 434.3, 458.0 | [M+Na]^+^: 675.2610 | 011444 |
| crocin V | 6.30 | 259.3, 433.0, 457.1 | [M+Na]^+^: 513.2090 | 011442 |
| crocetin | 7.84 | 256.1, 426.6, 451.5 | [M+H]^+^: 327.1605 | 011436 |
| crocetin semialdehyde | 8.68 | 226.0, 420.0, 444.1 | [M+H]^+^: 311.1686 | 011437 |
| crocetin dialdehyde | 9.25 | 265.3, 443.5, 467.7 | [M+H]^+^: 297.1939 | 011438 |
| zeaxanthin | 11.70 | 277.0, 451.3, 476.3 | [M+H]^+^: 569.4366 | 011439 |
| 8′-apo-β-carotenal | 13.50 | 269.3, 335.2, 452.4 | NMR |  |
| β-carotene | 25.52 | 275.6, 449.2, 474.0 | [M+H]^+^: 537.4364 | 011435 |
