# Supplementary material for: Tandem gene duplications drive divergent evolution of caffeine and crocin biosynthetic pathways in plants
Source: BMC Biol. 2020 Jun 18;18:63. doi: 10.1186/s12915-020-00795-3 (PMC7302004; doi:10.1186/s12915-020-00795-3)
Supplement: Supplementary file 3 — Additional file 3. Detailed methods and results for crocin biosynthesis. [file 12915_2020_795_MOESM3_ESM.docx]

**Additional file 3: Detailed methods and results for crocin biosynthesis**

*Identification of gene families related to crocin biosynthesis.* Protein sequences of the CCD, ALDH, and UGT family members in *A. thaliana* were downloaded from the TAIR database, then were used as queries in BLASTP searches against the *G. jasminoides* protein sequences to identify homologous sequences. Full-length protein sequences were corrected and aligned with ClustalW2 ([Larkin et al., 2007](#_ENREF_28)). Phylogenetic trees were constructed using the Maximum Likelihood method with the Jones-Taylor-Thornton (JTT) model and 1,000 Bootstrap replicates ([Kumar et al., 2018](#_ENREF_27)). Further analyses incorporated blast searches (using Gardenia proteins as queries) of a number of other genomes to identify more CCD, ALDH, and UGT genes. For NMTs, the Coffea canephora XMT protein was used as a query (NCBI accession ABD90685.1). Species considered were *Gardenia jasminoides* (CoGe genome ID 53980), *Coffea canephora* (CoGe genome ID 19443), *Arabidopsis thaliana* (CoGe genome ID 16911), *Calotropis gigantea* (CoGe genome ID 36623), *Catharanthus roseus* (CoGe genome ID 36703), *Vitis vinifera* (CoGe genome ID 19990), *Gelsemium sempervirens* (CoGe genome ID 53941), and *Solanum lycopersicum* (CoGe genome ID 12289). Gene model IDs from the respective CoGe-uploaded genomes were retained as leaf IDs for phylogenetic analysis, with the exception that “:” when it appeared in a gene model ID was replaced by “_”. Several additional anchoring protein sequences from NCBI were incorporated in the NMT tree (MTL,_AFV60456.1; DXMT,_ABD90686.1; MXMT,_AFV60445.1; XMT,_ABD90685.1). Searches were run on the CoGe platform using default parameters and saving 100 Blast HSPs per species. Unique translated sequences were then downloaded, duplicates were excluded using BBedit, sequences with internal stop codons were excluded, and then trees were run using PASTA ([Mirarab et al., 2015](#_ENREF_35)) with MAFFT ([Katoh et al., 2002](#_ENREF_24)) to align the protein sequences, and FastTree ([Price et al., 2010](#_ENREF_43)) to create an approximately-maximum-likelihood tree. Trees were visualized and edited using FigTree (<http://tree.bio.ed.ac.uk/software/figtree/>) (Fig. S27-30). To interpret the supplemental figures, pink branches represent gentianalean clades, green branches represent Rubiaceae clades, and orange gene model IDs represent Gardenia genes. Coffee-specific clades are shown in red. In the NMT supplemental tree (Fig. S27), the anchoring protein sequences are shown in red.

*Enzymatic activity assays* *and LC/LC-MS analyses.* The cDNAs of candidate genes from the *CCD*, *ALDH* and *UGT* families were synthesized and cloned into expression vectors via digestion and ligation (Fig. S25). The GjALDH2C3, GjUGT94E13 and GjUGT74F8 proteins were purified from *E. coli* using affinity chromatography to a purity >95% (Fig. S15, S16, S19). The *in bacterio* and *in vitro* activity assays and detailed reaction mixtures are described in the supplemental information.

Samples were analyzed using a Thermo Ultimate 3000 system equipped with an Acquity UPLC® BEH C18 column (1.7 μm, 100 × 2.1 mm). A gradient elution procedure was applied, using the mobile phases acetonitrile containing 0.1% formic acid (A) and water containing 0.1% formic acid (B). The following gradient elution program was used at a flow rate 0.3 mL/min: 0-5 min, 10% A linearly increased to 50% A; 5-8 min, 50% A linearly increased to 90% A; 8-10 min, 90% A linearly increased to 100% A, and sustained for 20 min; 30-31 min, back to 10% A.

Qualitative analysis of each compound was carried out using liquid chromatography-mass spectrometry (Agilent Technologies 1290 Infinity Ⅱ and 6545 Q-TOF, with Dual Agilent Jet Stream Electrospray Ionization sources). The drying gas was set at 325°C with a flow rate of 6 L/min, and the sheath gas was set at 350°C, with a flow rate of 12.0 L/min. The nebulizer was set at 45 psig, and the VCap was set at 4000 V. The data were analyzed using MassHunter (version B.07.00). The detailed information of mentioned compounds was listed in Table S17, S18.

Nuclear magnetic resonance (NMR) experiments were performed on Bruker AV III 600 NMR spectrometer (600 MHz for ^1^H NMR and 150 MHz for ^13^C NMR) in CDCl_3_ (Sigma–Aldrich, USA), and the chemical shifts were given in *δ* (ppm) with TMS as the internal standard.

*Detection of crocins/crocetin* *and isolation of crocins*. Detection of crocins: Seven different *G. jasminoides* tissues (roots, stems, leaves, flowers, fruitlets, green fruits, and red fruits) were collected and analyzed for determination of the crocin content. The tissues were freeze-dried, ground into powder, and extracted with 50% methanol in an ultrasonic bath for 30 min. Three different extraction were performed for each tissue. The extracts were subjected to UPLC analysis (ACQUITY UPLC BEH C18 1.7 μm, 2.1 × 100 mm; Waters) using the mobile phases water and acetonitrile (ACN) at a total at 0.25 mL/min flow rate. The separation of metabolites was achieved using the following gradient program: 0-5 min, 10-40% ACN; 5-10 min, 40% ACN; 10-11 min, 40-100% ACN; and 11-13 min, 100% ACN. The relative contents of different crocins were calculated based on the peak areas (Fig. S5, S6). This analysis revealed a significant accumulation of crocins in green and red fruit with red sarcocarps.

Detection of crocetin: dried gardenia fruits and flowers were ground to a coarse powder and extracted with 50% methanol as above described. The extract was dried in a vacuum concentrator, resuspended in water, extracted twice with ethyl acetate and subjected to UPLC analysis as above described. Three different extraction were performed for each tissue. The analysis revealed that crocetin accumulates in fruits but not in flowers (Fig. S7).

Isolation of crocins: dried gardenia fruits were ground to a coarse powder and extracted with 40% ethanol three times. The alcoholic extract was dried in a vacuum concentrator, resuspended in water, and then added with ethyl acetate. The water layer was subjected to HP-100 macroporous absorptive resin and eluted with water containing increasing amounts (0%, 25%, 60%) of ethanol. The 60% ethanol fractions were combined, dried in a vacuum concentrator, separated by silica gel column chromatography, and eluted with ethyl acetate containing increasing amounts of methanol–water (16:13). The eluted samples were further purified to yield crocins on a preparative ODS column (250×10 mm, 5 μm) using 45% methanol as the mobile phase at a flow rate of 2 mL/min.

*Identification of candidate genes involved in crocin biosynthesis in G. jasminoides*. Fourteen *CCD* genes were identified (four *CCD1*, four *CCD4*, one *CCD7*, three *CCD8*, and two *NCED* genes) (Table S10). In *Crocus sativus*, *CsCCD2* has been shown to catalyze the conversion of zeaxanthin to crocetin dialdehyde; however, no *CCD2* genes were found in *G. jasminoides*. The *GjCCD4* and *GjCCD8* gene families have been subjected to expansion; four *CCD4* genes (*GjCCD4a*, *GjCCD4b*, *GjCCD4c* and *GjCCD4d*) were found in tandem gene clusters <100 kb long (Fig. 6, Fig. S8). Three *CCD8* genes (*GjCCD8a*, *GjCCD8b*, and *GjCCD8c*) were also identified in a gene cluster, with three unknown genes localized in the intergenic region between *GjCCD8b* and *GjCCD8c*. *GjCCD4a* was highly expressed in flower, green and red fruits and mature sarcocarp (Fig. 3, Table S11). *GjCCD4a*, *GjCCD4c* and *GjCCD4d* were also highly expressed in flowers. None or very low expression (FPKM <1) was observed for all *CCD8* genes in all the tissues analyzed.

Eighteen *ALDH* genes were identified and classified into ten distinct subfamilies, comprising six *ALDH2*, two *ALDH3*, one *ALDH5*, three *ALDH6*, one *ALDH7*, one *ALDH10*, one *ALDH11*, one *ALDH12*, one *ALDH18*, and one *ALDH22* genes (Fig. S9). The numbers and subgroups of the ALDH family in *G. jasminoides* are similar to those in *A. thaliana* and *O. sativa*. Two pairs of *GjALDH2* family genes, *GjALDH2C1* (Gj6A297T86.1) *- GjALDH2C4* (Gj6A297T87.1) and *GjALDH2C2* (Gj9A24T69.1) *- GjALDH2C3* (Gj9A24T70.1), are located next to each other less than 4 kb apart in the genome according to the GFF information. The different *GjALDH* genes have different expression profiles (Fig. 3). *GjALDH2C2*, *GjALDH2C3* and *GjALDH2C4* are significantly co-expressed with *GjCCD4a* with Pearson’s correlation coefficients of *r* = 0.80, 0.78, and 0.89, respectively (Table S12). *GjALDH2C3* is the most highly expressed of all GjALDH genes, with an expression pattern comparable to that of *GjCCD4a* (high expression observed in green and red fruits and flowers, Fig. 3, Table S12).

We identified 237 *UGT* genes, which were classified into 19 groups (*UGT708*, 71, 72, 73, 74, 75, 76, 78, 79, 80, 81, 84, 85, 87, 88, 89, 90, 92 and 94) based on phylogenetic analysis (Fig. S10). These *UGTs* were differentially expressed in various organs of *G. jasminoides*, and 18.5% of them showed low or no expression in all of the samples analyzed (FPKM < 1) (Table S13). Eleven *UGT* genes were selected to study the biochemical function according to their relatively high expression in matured fruits (Figure 3, Table S13).

*Characterization of GjCCD4a in bacterio*. GjCCD4a CDS was cloned *Kpn* Ⅰ/*EcoR* Ⅰ in the bacterial expression vector pET-32a(+) vector, obtaining the pET32a-GjCCD4a construct. The construct was verified by sequencing. The pET32a-*GjCCD4a* was co-transformed into *E. coli* BL21 (DE3) cells with pACCAR25ΔcrtX (zeaxanthin accumulation plasmid), or pACCRT-EIB (lycopene accumulation plasmid) or pACCAR16Δcrt (β-carotene accumulation plasmid). As negative control, the pET32a empty vector was used instead of pET32a-*GjCCD4a*. The recombinant strains were grown in 40 mL LB (Luria–Bertani) medium with 50 μg mL^-1^ ampicillin and 34 μg mL^-1^ chloramphenicol and protein expression induced with 0.3 mM IPTG (isopropyl-β-D-thiogalactopyranoside) at 15 ℃, 130 rpm for 24 hours.

All the recombinant strains harboring the pET32a-*GjCCD4a* showed a discoloration compared to the cells harboring the pET32a empty vector. For UPLC-MS analysis, cells were harvested by centrifugation at 7830 g for 10 min, and pellets extracted was extracted with acetone until complete pellet discoloration. Extracts were then vacuum-dried and dissolved in 200 μL of methanol. UPLC analysis revealed the appearance of a new peak at 9.25 min that matched the retention time and spectrum of the crocetin dialdehyde standard in all the recombinant clones harboring the pET32a-*GjCCD4a* plasmid. MS positive mode analysis revealed that the *m/z* value of the new peak was 297.1939, that corresponds to the M+H^+^ ion of crocetin dialdehyde. Moreover, the characteristic fragment ions correspond to those of crocetin dialdehyde (Fig. 4, S12). An intermediate product, β-apo-8′-carotenal (NMR data in Fig. S13), derived from the asymmetric cleavage of β-carotene at the 7,8 or 7′8′ positions was also detected. Thus, GjCCD4a symmetrically cleaves the 7,8 and 7′,8′ double bonds in zeaxanthin, β-carotene and lycopene *in bacterio*.

*Protein modeling and docking of GjCCD4a*. The protein structure modeling and docking analysis of GjCCD4a showed high affinities with β-carotene, zeaxanthin and lycopene, in accordance with its catalytic activity (Fig. S14). The 2biw (pdb) model which showed 27.8 % homology (sequence identity) with GjCCD4a was used to construct the protein structure of GjCCD4a with catalytic Fe^2+^ ion. Docking analysis performed with Autodock 4.2.5.1 (http://autodock.scripps.edu/), showed higher affinity of GjCCD4a for β-carotene compared to zeaxanthin and lycopene, in accordance to the results of *in bacterio* GjCCD4a assay (Fig. 4A).

*Characterization of GjALDH2C3 in vitro.* The *GjALDH2C3* CDS was cloned into the pCold I expression vector using *BamH* I and *Sal* I restriction enzymes, and the pCold I - *GjALDH2C3* plasmid was transformed into *E.coli* BL21 cells. Recombinant cells were grown in 40 mL LB medium with 50 μg mL^-1^ ampicillin and protein expression induced by the addition of 0.3 mM IPTG and incubation for 24 hours at 15 ℃, 130 rpm. Cells were then harvested by centrifugation and the GjALDH2C3 protein purified by Ni^2+^-chelating affinity chromatography (GE, USA) (Fig. S15). Protein concentration was evaluated using the BCA protein assay (Takara, Japan). *In vitro* assay was performed at 30℃ for 3 hours in 50 μL of 100 mM Tris-HCl (pH 8.5), containing 50 µg of purified protein, 1 mM NADP^+^, and 40 μM crocetin dialdehyde. The reaction was stopped by the addition of 100 μL of acetone, and metabolites extracted with 300 μL of ethyl acetate. The extract was then vacuum-dried, dissolved in 200 μL of methanol, and subjected to UPLC-MS analysis. Two new chromatographic peaks were detected, and the retention time (RT=7.84 min) and spectrum of the major peak were the same as those of the crocetin standard. We further determined the structure of the new peaks by MS in negative mode. The molecular weight and characteristic fragment ions of the new peak were identical to those of crocetin and crocetin semialdehyde (Fig. 4, S15). Therefore, *in vitro* results provided evidence that GjALDH2C3 is able to catalyze the conversion of crocetin dialdehyde into its carboxylic acid crocetin.

*Glycosylation of crocetin to crocins*. Twelve candidate *UGT* genes (marked with an asterisk in Table S13) were cloned in the pET32a expression vector and introduced into *E. coli* BL21 (DE3) cells. Recombinant cells were grown in LB medium with 50 μg mL^-1^ ampicillin, and protein expression was induced by adding 0.3 mM IPTG and incubating cells for 24 h at 15 ℃, 130 rpm. Cells were then harvested by centrifugation and subjected to the preparation of a crude protein extract using phosphate buffer saline and ultrasonic fragmentation as well as to proteins purification by Ni^2+^-chelating affinity chromatography, as previously described (Fig. S16, 19). *In vitro* assays were performed in reaction mixes (200 μL) containing 100 mM Tris-HCl (pH 8.5), 1 mM TCEP, 5 mM UDP-glucose, 40 μM crocetin/crocins and 50 μL of crude lysate (or 50 μg purified proteins). The reactions were carried out for different time (overnight, 0.5 h, 1 h, or 3 h) at 30℃ and stopped by the addition of 100 μL of methanol. The samples were centrifuged with 15,000 rpm for 10 min, and then then subjected to UPLC-MS analysis.

In the presence of the purified GjUGT94E13 enzyme, using crocetin as substrate, we observed the appearance of two new peaks. The first peak had the same chromatographic mobility (RT= 4.22 min) of the authentic standard crocin I (Fig. S16). The MS analysis of this peak revealed a molecular ion [M+Na]^+^ with *m/z* 999.3699, that corresponds to that of crocin I. In addition, an ion with *m/z* 675.2642 [M+Na]^+^, generated by in source deglycosylation of crocin I (elimination of a terminal gentiobiosyl group) was found (Fig. 4, S16). The second peak (RT= 5.75 min) exhibited a molecular ion [M+Na]^+^ with *m/z* 675.2610, that corresponds to crocin IV. The MS/MS spectra revealed the presence of a [M+Na]^+^ ion fragment at *m/z* 351.1559, corresponding to crocetin (molecular mass of 328) (Fig. 4, S16). Thus, the purified GjUGT94E13 protein is able to glycosylate crocetin into crocin IV and crocin I, carrying a single β-D-gentiobiosyl ester and a symmetrical double β-D-gentiobiosyl ester, respectively. In the presence of the purified GjUGT94E13 enzyme, using crocin II and crocin III as substrates, a new peak, corresponding to crocin I, was detected via UPLC (Fig. S17, 18).

In the presence of the purified GjUGT74F8 enzyme, using crocetin as substrate, we observed the appearance of two new peaks, with RT of 4.96 min and 6.30 min, and *m/z* values of 675.2620 ([M+Na]^+^), and 513.2090 ([M+Na]^+^), corresponding to crocin III and crocin V, respectively (Fig. S19). These results demonstrated that GjUGT74F8 has primary glycosylation activity and can add β-D-glucosyl moieties to the carboxyl groups of crocetin. When we tested the activity of the purified GjUGT74F8 enzyme towards crocin II, we observed the formation of a new product that has the RT and mass spectrum of crocin IV (Fig. S20).

We also characterized the previously identified GjUGT75L6 enzyme (Nagatoshi et al., 2012). Our results confirmed that GjUGT75L6 is able to glycosylate crocetin into crocin V and crocin III, which are single and a symmetrical double β-D-glucosyl esters, respectively (Fig. S21). In fact we observed two new peaks with RT of 4.96 min and 6.30 min and molecular ions [M+Na]^+^: *m/z* 675.2609 and 513.2105 that correspond to those of crocin III and crocin V, respectively. However, the low transcript levels in mature fruit of *GjUGT75L6* indicate that the corresponding enzyme is not the main enzyme involved in the primary glycosylation of crocetin in *G. jasminoides* fruits.
