# Supplementary figures and images for: Tandem gene duplications drive divergent evolution of caffeine and crocin biosynthetic pathways in plants
Source: BMC Biol. 2020 Jun 18;18:63. doi: 10.1186/s12915-020-00795-3 (PMC7302004; doi:10.1186/s12915-020-00795-3)

Additional file 4: Figure S27. Pasta phylogenetic tree of NMTs from different species.

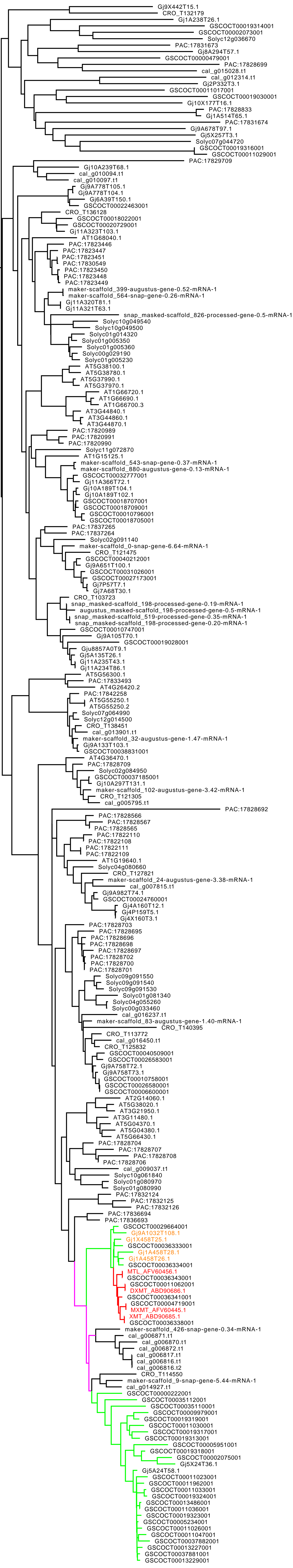

0.5

Supplement: Supplementary file 4 — Additional file 4: Figure S27. Pasta phylogenetic tree of NMTs from different species. [file 12915_2020_795_MOESM4_ESM.pdf]

Additional file 5: Figure S28.  
Pasta phylogenetic tree of  
CCDs from different species.

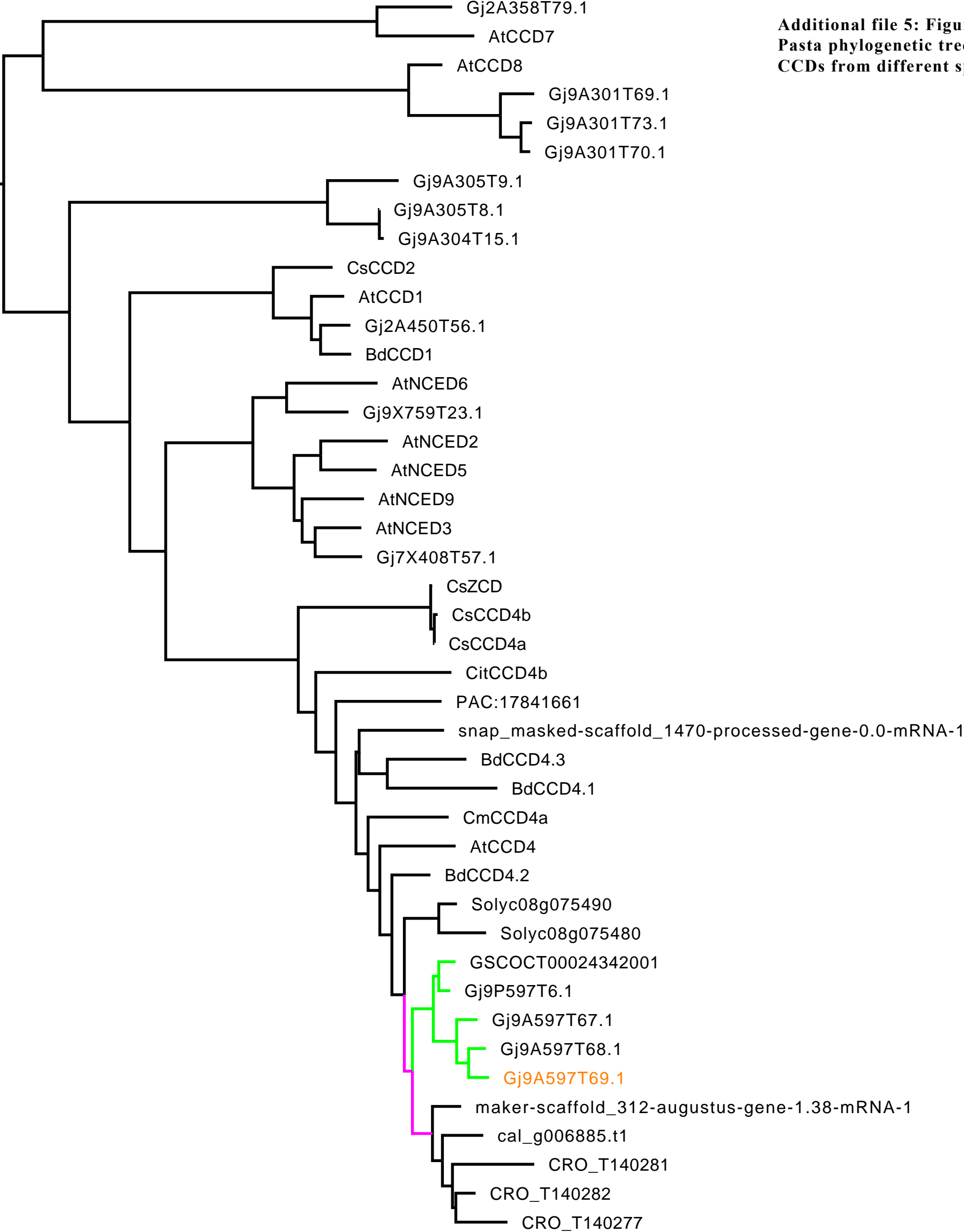

Supplement: Supplementary file 5 — Additional file 5: Figure S28. Pasta phylogenetic tree of CCDs from different species. [file 12915_2020_795_MOESM5_ESM.pdf]

Additional file 6: Figure S29. Pasta phylogenetic tree of UGTs from different species.

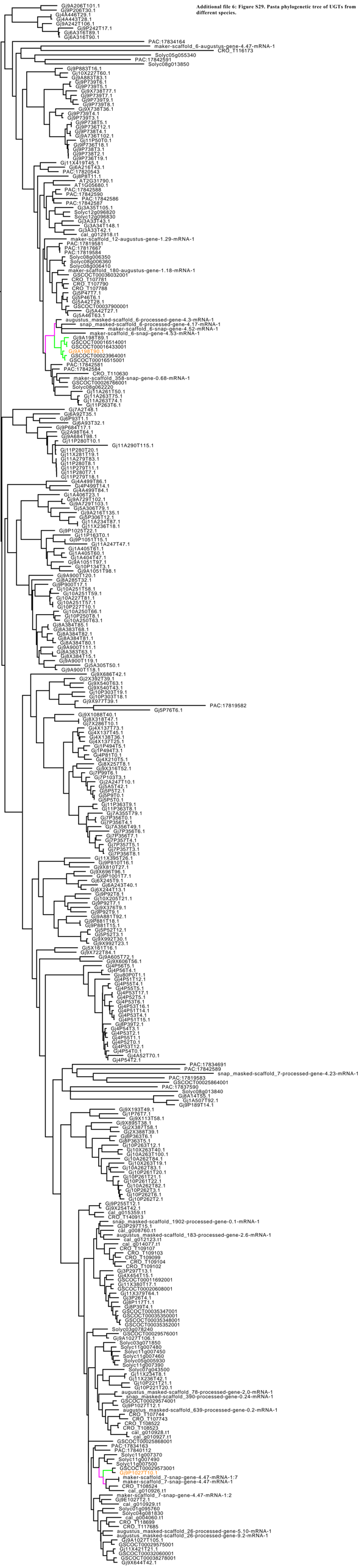

Supplement: Supplementary file 6 — Additional file 6: Figure S29. Pasta phylogenetic tree of UGTs from different species. [file 12915_2020_795_MOESM6_ESM.pdf]

Additional file 7: Figure S30. Pasta phylogenetic tree of ALDHs from different species.

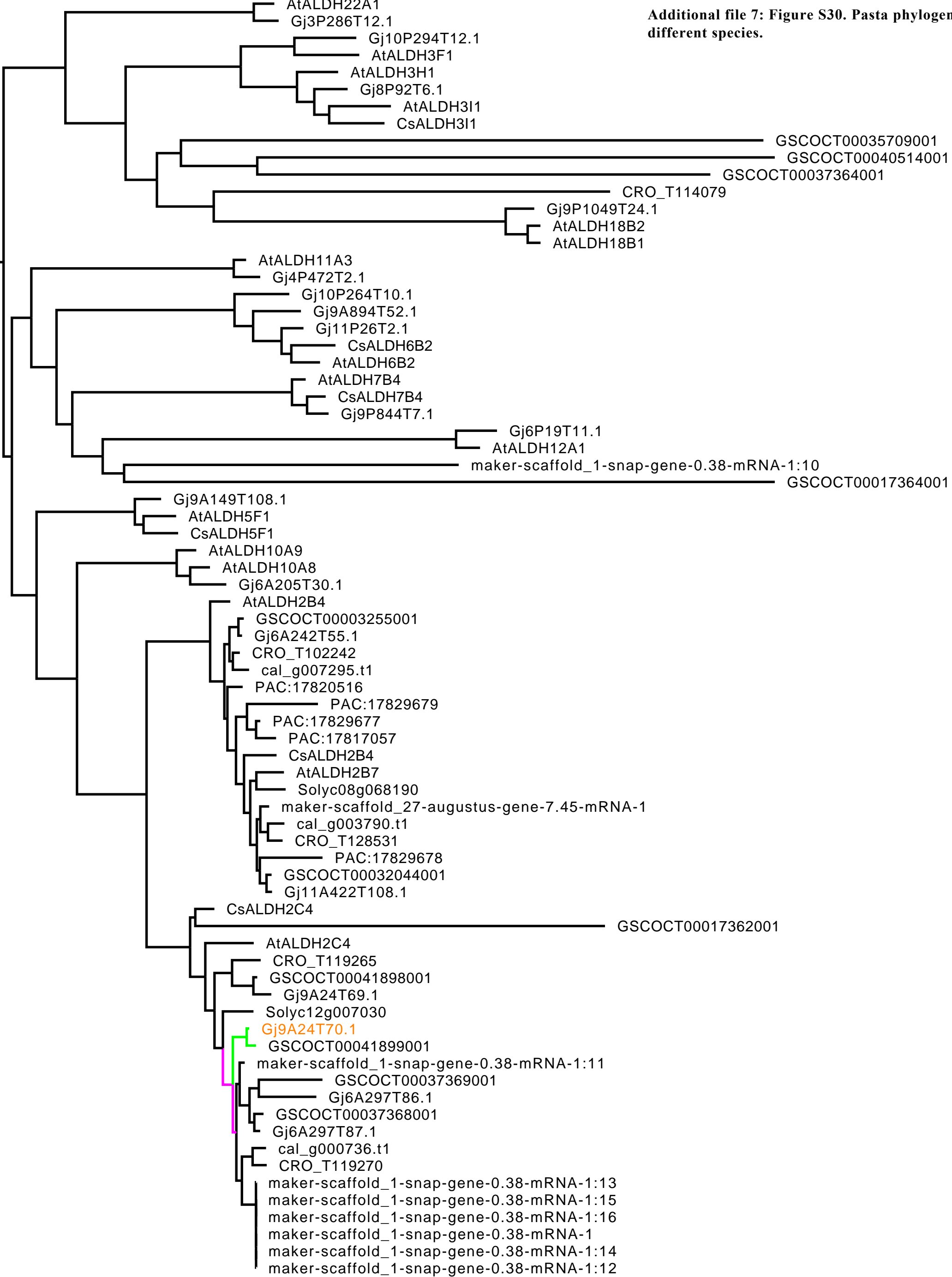

Supplement: Supplementary file 7 — Additional file 7: Figure S30. Pasta phylogenetic tree of ALDHs from different species. [file 12915_2020_795_MOESM7_ESM.pdf]
